# Patient-Clinician Communication Interventions Across Multiple Primary Care Sites: A Cluster Randomized Clinical Trial

**DOI:** 10.1001/jamahealthforum.2024.4436

**Published:** 2024-12-13

**Authors:** Ming Tai-Seale, Michael Cheung, Florin Vaida, Bernice Ruo, Amanda Walker, Rebecca L. Rosen, Michael Hogarth, Kimberly A. Fisher, Sonal Singh, Robert A. Yood, Lawrence Garber, Cassandra Saphirak, Martina Li, Albert S. Chan, Edward E. Yu, Gene Kallenberg, Christopher A. Longhurst, Marlene Millen, Cheryl D. Stults, Kathleen M. Mazor

**Affiliations:** 1 University of California, San Diego, School of Medicine, La Jolla; 2 University of California, San Diego, Herbert Wertheim School of Public Health and Human Longevity Science, La Jolla; 3 University of Massachusetts Chan Medical School, Worcester; 4 Reliant Medical Group, Worcester, Massachusetts; 5 Sutter Health, Palo Alto Medical Foundation, Palo Alto, California

## Abstract

**Importance:**

Despite various attempts to improve patient-clinician communication, there has been limited head-to-head comparison of these efforts.

**Objective:**

To assess whether clinician coaching (mobile application or in-person) is more effective than reminder posters in examination rooms and whether mobile app use is noninferior to in-person coaching.

**Design, Setting, and Participants:**

A cluster randomized clinical trial with 3 arms. A total of 21 primary care clinics participated in 3 health systems in the US; participants were patients and primary care clinicians with clinic visits between August 28, 2019, and December 31, 2021. Data were analyzed from August 4, 2022, to November 10, 2024. Data analysis was based on intention to treat.

**Interventions:**

In-person coaching of clinicians with standardized patient instructor training (high-touch), mobile application–based coaching (high-tech), and posters placed in examination rooms encouraging shared decision-making (AskShareKnow [ASK]). Before visits, patients in the high-touch and high-tech groups were prompted to inform their clinicians of the most important matter for discussion using online check-in.

**Main Outcomes and Measures:**

The primary outcome was a patient engagement measure (CollaboRATE) of patient perceptions of communication and shared decision-making during the visit. Difference-in-differences mixed-effect regression with random intercepts for primary care clinician were used for analyses. Secondary outcomes included the net promoter score (patient’s likelihood of recommending this clinician to others) and patient’s confidence in managing their health.

**Results:**

Participants included 4852 patients with a median age of 54 years (IQR, 39-66 years); 63.6% were women. A total of 114 clinicians (median age range, 40-59 years; 48 were women [42.1%]) participated. The 3 interventions did not differ significantly in probability of CollaboRATE top score (marginal difference, high-tech vs ASK, –0.021; 95% CI, –0.073 to 0.030; high-touch vs ASK, –0.018, 95% CI, –0.069 to 0.033; high-tech vs high-touch, –0.003, 95% CI, –0.057 to 0.052; *P* = .14). Patients in the high-tech group were less likely to recommend their clinician to others than patients in the high-touch group (difference in marginal probability, –0.056; 95% CI, −0.118 to 0.019; *P* = .04). After 3 months, patients in the high-tech group had a significantly lower score than patients in the high-touch group (mean difference, −0.176; 95% CI, −0.341 to −0.011; *P* = .04) in confidence in managing their health.

**Conclusions and Relevance:**

This cluster randomized clinical trial found no evidence of intervention effects, although there were differences across systems. Some secondary outcomes suggested positive effect of clinicians receiving in-person coaching. Alternative outcome measures of patient engagement may be needed.

**Trial Registration:**

ClinicalTrials.gov Identifier: NCT03385512

## Key Points

**Question:**

Can interventions developed in a pilot study be modified and implemented to improve patient engagement in multiple health systems?

**Findings:**

In this cluster randomized trial of 21 health systems including 4852 patients, patient engagement showed no evidence of effects of the interventions. Secondary outcomes showed some evidence of patient likelihood to recommend their clinicians and patient confidence in managing their health.

**Meaning:**

The results of this study suggest that implementation fidelity and alternative outcome measures for patient engagement are needed.

## Teaser

This cluster randomized clinical trial assesses whether clinician coaching (mobile application or in-person) is more effective than reminder posters in examination rooms in improving patient-clinician communication.

## Introduction

How clinicians communicate with patients during clinical encounters can affect patients’ health^1,2^ and the quality of care.^3,4,5^ It has been well documented that clinicians often do not know patients’ reason for visits before they meet face to face with each other.^6,7^ Many patients cannot recall details of the decisions made during their visits.^8,9,10^

While many efforts to improve patient-clinician communication have been attempted,^11,12,13,14,15^ their effectiveness has rarely been compared head to head. This study builds on a previous pilot study^13^ that created 2 interventions aimed at enhancing patient-clinician communication that was highly accessible to users. We chose the primary care setting for 3 reasons: it is essential for health care,^16^ most shared decision-making research is in specialty care, and the pilot study in primary care showed potential utility. This study aimed to replicate and expand those findings in diverse settings.

## Methods

### Study Design and Participants

This cluster randomized trial was conducted in 3 health systems (UC San Diego Health, Reliant Medical Group, and Sutter Health) anonymized as HS1, HS2, and HS3. Primary care clinicians were invited to join. Eligible patients (aged ≥18 years, electronic patient portal [MyChart; Epic] users, English speaking, with a scheduled appointment in 2-14 days) received email or electronic patient portal invitations and provided informed consent. The Consolidated Standards of Reporting Trials extension (CONSORT Extension) to cluster randomized trials reporting guideline was followed. The trial protocol is available in Supplement 1.

The study had 2 phases: baseline and postintervention. At baseline, patient surveys assessed clinicians’ communication. After the intervention, different patients completed surveys within 7 days of their visit and again at 3 months. The same clinicians participated in both phases.

Data for sociodemographic and outcomes were obtained from surveys (eg, race and ethnicity, patient-reported outcomes) or the electronic health record (EHR) (eg, social vulnerability index, encounter types). The institutional review board of each system approved the study. Although all 3 institutional review boards concluded that the study posed minimal risk to patients, potential concerns remained regarding privacy. These involved analysts linking survey and health data, as well as health systems sharing limited datasets externally for analysis, necessitating informed consent.

### Randomization

The study team engaged clinic leadership to secure participation, presented the study at meetings to inform clinicians, and obtained informed consent from those interested. Clinics were randomly assigned to the 3 interventions (1:1:1), stratified by health system, to (1) in-person coaching of clinicians with standardized patient instructors (SPI) (high-touch), (2) mobile application–based coaching of clinicians (high-tech), and (3) posters in examination rooms encouraging shared decision-making (AskShareKnow [ASK]), by the study statistician (F.V.). Clinics were chosen as the randomization unit to minimize cross-contamination of interventions (Figure).

**Figure.**
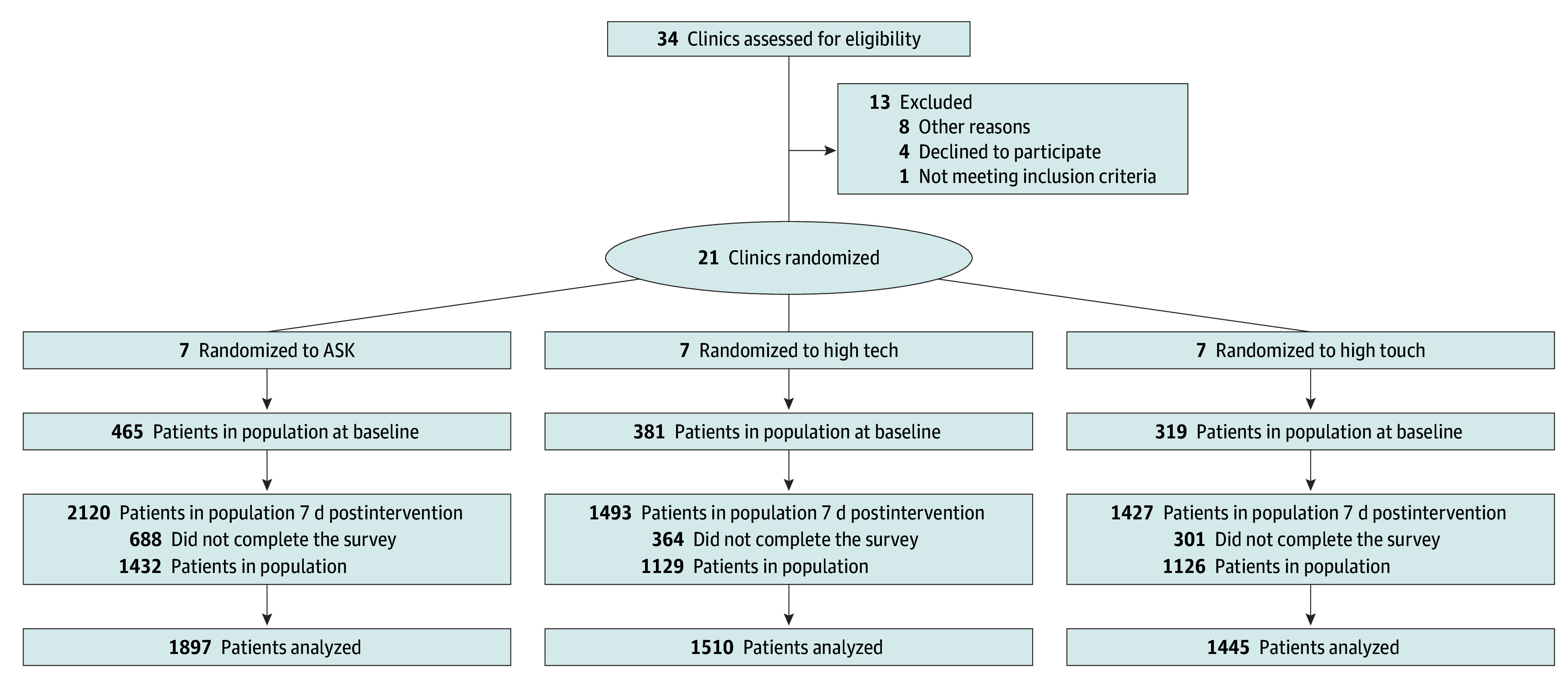
Participant Flow Through the Trial Patients who had participated in baseline were not eligible for the postintervention phase. Clinicians were clustered within the 21 clinics with an average of 5 clinicians per clinic. A total of 49 clinicians were allocated to ASK (AskShareKnow), 38 to high-tech, and 32 to high-touch; 5 clinicians dropped out prior to baseline, resulting in 46 ASK, 37 high-tech, and 31 high-touch clinicians. Nine clinicians who had no postintervention visits were considered lost to follow-up: 4 in ASK and 5 in high-tech.

Patients of participating clinicians were invited via email to provide electronic consent after clinics were randomized to treatment arms in the postintervention phase. Due to the nature of the interventions, neither patients nor clinics were blinded to the randomization (Supplement 1).

### Interventions

The interventions are multilevel, targeting patients, care teams (clinicians, nurses, medical assistants), and systems.^17,18^ The high-touch and high-tech arms included a 3-minute animated video promoting joint agenda setting, question asking, shared decision-making, and ensuring patient understanding of next steps. Patients prepared for their visits by indicating their most important topics via online check-in through the patient portal.^12^ Clinicians were nudged to review these priorities, acknowledge them at the visit’s start, set agendas collaboratively, engage in shared decision-making, confirm patient understanding through teach-back, and document the care plan in the after visit summary. As in the pilot study,^17^ SPIs coached high-touch clinicians in two 30-minute sessions, while a mobile app provided high-tech clinicians with similar content (eTable 1 in Supplement 3).

In the ASK arm,^19^ posters with these questions were placed in examination rooms: (1) What are my options? (2) What are the possible benefits and risks of each option? and (3) How likely are the benefits and risks of each option to occur to me? The poster is reproduced as the eFigure in Supplement 3.

### Outcomes Assessment

Patient-reported outcomes were gathered using a link to the online postvisit questionnaire sent to patients within 1 business day of their visits; responses were included in the analyses if completed within 7 days. The primary outcome was a validated 3-item patient-reported outcome measure, known as CollaboRATE,^20^ which consisted of 3 questions about how much effort was made to (1) help you understand your health issues; (2) listen to the things that matter most to you about your health issues; and (3) include what matters most to you in choosing what to do next. A scale of 0 (definitely disagree) to 9 (definitely agree) was used. The secondary outcomes included (1) likelihood of recommending this clinician to others, known as the net promoter score (NPS),^21,22^ measured on a Likert scale (range, 1-5) with 5 coded as promoter, 4 as neutral, and 1 to 3 as detractor^23^; and (2) patients’ confidence in managing their health, adopted from the Health Information National Trends Survey,^24^ was measured by their overall confidence in their ability to take good care of their health using a 5-point Likert scale with 5 indicating completely confident and 1 indicating not confident at all.

### Sample Size and Power

The sample size calculations were first performed at study design, based on the primary binary end point of 3 top scores for CollaboRATE.^20^ The CollaboRATE top score was 1 if all 3 items of CollaboRATE received the top score. Otherwise, the top score was 0. We assumed that the patient outcomes were clustered within a clinician, and that the clinicians would be clustered within the 21 clinics. We planned to recruit 105 clinicians, with an average of 5 clinicians per clinic and a coefficient of variation of 0.3. Based on the pilot study,^13^ we assumed a clinician-related intraclass correlation coefficient of 0 but a clinic-related intraclass correlation of 0.001 or 0.002, which provided 81% or 78% power to detect a difference of 5% (77% vs 72%) in the primary comparison between the high-tech and ASK arms. This required that we enroll 10 patients per clinician during baseline and 40 patients per clinician postintervention.

### Missing Data

Patients provided informed consent before the previsit intervention and were asked to complete the postvisit survey within 7 days. Those who did not complete the survey in that time frame were considered lost to follow-up (LTFU).

### Statistical Analysis

The primary outcome of CollaboRATE top score was analyzed using mixed-effects logistic regression. Based on data from both baseline and intervention phases, the model included a term for phase, treatment arm, and their interaction. A significant phase-by-treatment interaction in each comparison between arms indicated a difference in intervention effects between arms. This hierarchical clustering model included a random effect for clinician and a random effect for clinic, to account for within-clinician and within-clinic correlations. The primary outcome comparison was of high-tech vs ASK, performed at the α = .05 level, as a superiority test. The secondary comparisons were of high-touch vs ASK and high-tech vs high-touch. The high-tech vs high-touch comparison was a noninferiority comparison with a noninferiority margin of 5% (chosen based on prior research^13^ and clinically meaningful differences endorsed by key stakeholders), performed as a secondary analysis at level α = .025 1-sided. The high-touch vs ASK comparison was a separate secondary analysis performed as a superiority comparison. No overall 3-arm comparison was planned. Intention-to-treat analysis was used, analyzing all data based on patients’ assigned intervention arms. Treatment effect sizes are presented as differences between treatment arms of change from baseline in population-level (marginal) probability of CollaboRATE top score. Since baseline preintervention probabilities are assumed equal for the 3 groups, these differences in differences of probabilities are equivalent to differences in probabilities between intervention groups. CIs for these effect sizes were computed via bootstrap using 10 000 resamples. The high-tech vs ASK comparison was prioritized due to lower cost and greater scalability of the high-tech intervention compared with the high-touch. While the interventions are similar, they differ mainly in education delivery: in-person vs mobile app. Although high-tech may not outperform high-touch, its cost and scalability make it preferable if its effectiveness is not inferior, justifying the noninferiority comparison.

Adjusted analysis of the CollaboRATE top score outcome controlled for patient age, sex, race and ethnicity, educational level, and social vulnerability index^25^; encounter type (in-person or telehealth visit) and visit reason (acute or nonacute). The COVID-19 pandemic prompted a design update to assess its impact, classifying visits as before or after March 13, 2020, when COVID-19 was declared a national emergency. Telehealth visit encounters were added in all 3 health systems, but only in-person visits in ASK were analyzed. Patient information was mainly sourced from the EHR, while data on race and ethnicity and educational level were obtained from surveys. Clinician surveys collected data on age, sex, race and ethnicity, medical specialty, and time since residency.

The NPS was analyzed using longitudinal ordinal logistic regression, with a time-by-treatment interaction and random effect for clinician, and effect size reported in marginal effects (differences in changes from baseline of probabilities associated with higher vs lower NPS, equivalent to differences between groups in probabilities of higher vs lower NPS). CIs were computed via 10 000 bootstrap resamples.^23^ Unadjusted and adjusted analyses of NPS were conducted and reported, similar to the CollaboRATE top score analysis. Patient confidence in managing their health was assessed after the index visit and 3 months later using a linear mixed-effects model with covariate adjustments. Pairwise comparisons prioritized high-tech vs ASK, followed by high-touch vs ASK and high-tech vs high-touch, all testing the null hypothesis of equal group means. Comparisons between health systems were Bonferroni-corrected. A similar approach was used for patients’ confidence in managing their health.

All analysis models and covariates were outlined in the statistical analysis plan finalized prior to the analyses (Supplement 2). Since the outcomes are of individual interest, multiple comparison adjustments were deemed unnecessary. All analyses used R version 4.2.2 (R Core Team) and regression models were implemented with the lme4 version 1.1-31 package. Data were analyzed from August 4, 2022, to November 10, 2024.

## Results

A total of 21 primary care clinics participated in 3 health systems in the US; participants were 4852 patients (median age, 54 years [IQR, 39-66 years]); 3086 [63.6%] were female, 1673 male [34.5%], and 93 other or missing sex [1.9%]) and 114 primary care clinicians (median age range, 40-49 years; 48 were female [42.1%], 26 male [22.8%], and 40 other or missing [35.1%]) with clinic visits between August 28, 2019, and December 31, 2021. Patient enrollment began at HS1 on August 28, 2019, at HS2 on December 20, 2019, and at HS3 on April 17, 2019. To establish a baseline of patient-reported experience for each participating clinician, we conducted patient surveys from January to July 2019 before implementing any clinician training in the randomly assigned clinics. Patients who had participated in baseline were not eligible for the postintervention phase. After the baseline patient survey, clinicians in high-touch and high-tech received training in 2019. ASK posters were placed in examination rooms from July 2019 through February 2021 after baseline surveys had been completed for all participating clinicians in the ASK clinics. We collected postintervention visit surveys from September 2019 to August 2021 after clinicians in high-touch or high-tech had completed communications training. A total of 4852 patients and 114 primary care clinicians in 21 clinics met the intention-to-treat eligibility criteria. The flow of patients and clinic and the number LTFU are depicted in the Figure. Patient participation (consent) rate among those invited to participate was 7%. Survey return rate within 7 days of the visit among the consented patients was 73%.

Patient characteristics (age, sex, self-reported race and ethnicity, and educational level) and clinician characteristics (age, sex, self-reported race and ethnicity, specialty, and time since residency) are reported by treatment arm in Table 1. eTable 2 in Supplement 3 provides details on patient demographic characteristics in baseline and postintervention phases.

**Table 1.** Patient and Clinician Characteristics by Study Arm

Characteristic	Study arm, No. (%)			Total, No. (%)
	ASK	High-tech	High-touch	
**Patient characteristics**				
No. of patients	1897	1510	1445	4852
Age, median (IQR), y	58.0 (42-68)	53.0 (39-64.9)	52.0 (37-64)	54.0 (39-66)
Sex				
Female	1137 (59.9)	1023 (67.7)	926 (64.1)	3086 (63.6)
Male	727 (38.3)	485 (32.1)	461 (31.9)	1673 (34.5)
Other or missing	33 (1.7)	2 (0.1)	58 (4.0)	93 (1.9)
Race and ethnicity^a^				
American Indian or Alaska Native	7 (0.4)	5 (0.3)	8 (0.6)	20 (0.4)
Asian	179 (9.4)	186 (12.3)	193 (13.4)	558 (11.5)
Black or African American	37 (2.0)	49 (3.2)	25 (1.7)	111 (2.3)
More than 1 race	33 (1.7)	29 (1.9)	35 (2.4)	97 (2.0)
Native Hawaiian or Other Pacific Islander	10 (0.5)	16 (1.1)	9 (0.6)	35 (0.7)
White	1537 (81.0)	1133 (75.0)	1100 (76.1)	3770 (77.7)
Missing	94 (5.0)	92 (6.1)	75 (5.2)	261 (5.4)
Hispanic or Latino ethnicity	129 (6.8)	151 (10.0)	109 (7.5)	389 (8.0)
**Clinician characteristics**				
No. of clinicians	46	37	31	114
Age, median range, y	40-49	40-49	50-59	40-49
Sex				
Female	18 (39.1)	18 (48.6)	12 (38.7)	48 (42.1)
Male	12 (26.1)	7 (18.9)	7 (22.6)	26 (22.8)
Other or missing	16 (34.8)	12 (32.4)	12 (38.7)	40 (35.1)
Race and ethnicity^a^				
Asian	14 (30.4)	9 (25.0)	4 (12.9)	27 (23.7)
Black or African American	0 (0.0)	0 (0.0)	1 (3.2)	1 (0.9)
More than 1 race	0 (0.0)	0 (0.0)	2 (6.5)	2 (1.8)
White	14 (30.4)	14 (38.9)	11 (35.5)	39 (34.2)
Missing	18 (39.1)	13 (36.1)	13 (41.9)	45 (39.5)
Hispanic or Latino ethnicity	1 (2.2)	1 (2.8)	2 (6.5)	4 (3.5)
Specialty				
Family medicine	17 (37.0)	23 (62.2)	8 (25.8)	48 (42.1)
Internal medicine	27 (58.7)	12 (32.4)	23 (74.2)	62 (54.4)
Physician assistant or nurse practitioner	2 (4.3)	1 (2.7)	0 (0.0)	3 (2.6)
Missing	0 (0)	1 (2.7)	0 (0)	1 (0.9)
Time since residency, median (IQR), y	10.0 (4.75-19.3)	16.0 (7.00-22.0)	13.0 (7.25-23.0)	14.0 (5.00-21.0)

Abbreviation, ASK, AskShareKnow.

^a^ Race and ethnicity were self-reported.

At baseline phase, 465 patients were consented in ASK, 381 in high-tech, and 319 in high-touch. At postintervention phase, the numbers of consented patients were 2120 for ASK, 1493 for high-tech, and 1427 for high-touch. The LTFU across the 3 arms were 688 (32.5%), 364 (24.4%), and 301 (21.1%); 193 patients in ASK (9.1%), 153 in high-tech (10.2%), and 143 in high-touch (10.0%) did not complete the 3-month postintervention survey.

A total of 49 clinicians were allocated to ASK, 38 to high-tech, and 32 to high-touch. Five clinicians dropped out prior to baseline, resulting in 46 ASK, 37 high-tech, and 31 high-touch clinicians. Nine clinicians who had no postintervention visits were considered LTFU: 4 in ASK and 5 in high-tech.

### Primary and Secondary Outcomes

For the primary outcome of CollaboRATE,^20^ the number and percentage of patients who chose the top score for all 3 CollaboRATE items for ASK at the baseline phase was 295 (63.9%), for the high-tech arm was 266 (70.2%), and for the high-touch arm was 216 (67.9%). At the intervention phase, the number and percentage of patients who chose the top score for all 3 CollaboRATE items for ASK was 964 (68.2%), for the high-tech arm was 752 (67.4%), and for the high-touch arm was 749 (67.0%) (Table 2). There were no statistically significant differences in unadjusted analyses between high-tech and ASK (difference in probabilities, –0.021; 95% CI, –0.073 to 0.030; *P* = .42) or high-touch and ASK (difference in probabilities, –0.018; 95% CI, –0.069 to 0.033; *P* = .48). However, high-tech was noninferior to high-touch (difference in probabilities, –0.003; 95% CI, –0.057 to 0.052; *P* = .02) (Table 3; eTable 3 in Supplement 3).

**Table 2.** Primary and Secondary Outcomes in 1165 Patients in the Baseline Phase

Outcome	Baseline phase, No. (%)		
	ASK (n = 465)	High-tech (n = 381)	High-touch (n = 319)
**Primary outcome**			
CollaboRATE top score^a^^,^^b^			
Intention-to-treat population	462 (39.9)	379 (32.7)	318 (27.4)
Top score, unadjusted	295 (63.9)	266 (70.2)	216 (67.9)
Top score, adjusted, %^c^	61.3	65.4	63.4
**Secondary outcome**			
“Likelihood of recommending this care clinician to others” as NPS^d^^,^^e^			
Unadjusted			
Promoter	391 (84.6)	319 (83.7)	272 (85.8)
Neutral	55 (11.9)	48 (12.6)	30 (9.5)
Detractor	16 (3.5)	14 (3.7)	15 (4.7)
Adjusted, %			
Promoter	76.8	72.6	77.1
Neutral	15.7	19.1	16.4
Detractor	7.5	8.3	6.4
Patients’ confidence in managing their health			
Unadjusted baseline score, mean (SD)	8.06 (1.35)	8.11 (1.13)	8.2 (1.04)
Adjusted baseline score, mean	7.86	7.89	7.92

Abbreviations: ASK, AskShareKnow; NPS, net promoter score.

^a^ If all 3 items of CollaboRATE received the top score, the binary CollaboRATE is top score. If any one item of CollaboRATE did not receive the top score, the binary CollaboRATE is not top score.

^b^ Mixed-effects logistic regression models were used to estimate the arm comparisons for CollaboRATE top score. The models included fixed effects for study phase, interaction statistical analysis between phase and intervention, health system, and random effects for primary care professionals (PCPs) with an additional random effect for health system 2 PCPs. The adjusted model also included terms for patient and PCP characteristics, visit type, and impact of COVID-19 pandemic (by classifying the visits as occurring up to March 13, 2020, or after this date, when COVID-19 was declared a national emergency).

^c^ Adjusted percentages and adjusted means were calculated by marginal standardization.

^d^ If the response to the item was a score of 5, the NPS was 1 (promoter). If the response to the item was a score of 4, the NPS was 0 (neutral). If the response to the item was a score of 1 to 3, the NPS was −1 (detractor).

^e^ A mixed-effects linear regression model was used to estimate the arm comparisons for NPS. The covariate specification was identical to that of the adjusted primary outcome model, excluding the random effect for health system 2 PCPs.

**Table 3.** Primary and Secondary Outcomes in 3687 Patients in the Postintervention Phase

Outcome	Postintervention phase, No. (%)		
	ASK (n = 1432)	High-tech (n = 1129)	High-touch (n = 1126)
**Primary outcome**			
CollaboRATE, top score^a^^,^^b^			
Intent-to-treat population	1414 (38.8)	1115 (30.6)	1117 (30.6)
Top score, unadjusted	964 (68.2)	752 (67.4)	749 (67.0)
Top score, adjusted, %^c^	67.0	69.4	69.9
**Secondary outcome**			
NPS^d^			
Unadjusted			
Promoter	1244 (87.0)	933 (83.0)	974 (86.8)
Neutral	136 (9.5)	144 (12.8)	116 (10.3)
Detractor	50 (3.5)	47 (4.2)	32 (2.9)
Adjusted, %			
Promoter	85.6	84.5	88.1
Neutral	10.8	11.5	8.9
Detractor	3.6	4.1	3.0
Patients’ confidence in managing their health			
Unadjusted postintervention score, mean (SD)	8.2 (1.08)	8.22 (1.09)	8.23 (1.06)
Unadjusted 3 mo postintervention score, mean (SD)	7.86 (1.27)	7.88 (1.28)	7.93 (1.18)
Adjusted postintervention score, mean^c^	8.20	8.23	8.23
Adjusted 3 mo postintervention, mean^c^	7.86	7.85	8.00

Abbreviations: ASK, AskShareKnow; NPS, net promoter score.

^a^ If all 3 items of CollaboRATE received the top score, the binary CollaboRATE is top score. If any one item of CollaboRATE did not receive the top score, the binary CollaboRATE is not top score.

^b^ Mixed-effects logistic regression models were used to estimate the arm comparisons for CollaboRATE top score. The models included fixed effects for study phase, interaction between phase and intervention, health system, and random effects for primary care professionals (PCPs) with an additional random effect for health system 2 PCPs. The adjusted model also included terms for patient and PCP characteristics, visit type, and impact of COVID-19 pandemic (by classifying the visits as occurring up to March 13, 2020, or after this date, when COVID-19 was declared a national emergency).

^c^ Adjusted percentages and adjusted means were calculated by marginal standardization.

^d^ If the response to the item was a score of 5, the NPS was 1 (promoter). If the response to the item was a score of 4, the NPS was 0 (neutral). If the response to the item was a score of 1 to 3, the NPS was −1 (detractor).

We compared the proportion of CollaboRATE top scores between arms, adjusting for covariates. Older patients and patients with higher confidence in their ability to manage their health had a significantly higher chance of giving top scores on all CollaboRATE items. Patients who were not female, patients who had telehealth visits (as compared with in-person), patients of clinicians with minority race and ethnicity, and patients in HS2 (compared with patients in HS1) had a significantly lower chance of giving top scores on all CollaboRATE items (eTable 4 in Supplement 3).

### Net Promoter Score

The proportions of promoters in the 3 arms postintervention were high-tech 83.0, high-touch 86.8%, and ASK 87.0%. There were no significant differences between high-tech and ASK, marginal difference in probability of being a promoter –0.024; 95% CI, −0.098 to 0.054; *P* = .42, or high-touch vs ASK, marginal difference in probability of being a promoter, 0.032; 95% CI, −0.045 to 0.103; *P* = .22. For the high-tech vs high-touch comparison, the marginal difference in probability of being a promoter was –0.056; 95% CI, −0.118 to 0.019; *P* = .04 (Table 4). While the significant P value, based on asymptotic statistical methods, indicates a higher probability of being a promoter for high-touch, the bootstrap CI includes the value 0, meaning that the finding is not statistically robust.

**Table 4.** Outcomes and Measure From the Arm Comparisons in 4852 Patients

Outcome and measure	Arm comparison (95% CI)^a^		
	High-tech vs ASK	High-touch vs ASK	High-tech vs high-touch
**Primary outcome**			
CollaboRATE^b^			
Average marginal effect			
Unadjusted top score	−0.021 (−0.073 to 0.030)	−0.018 (−0.069 to 0.033)	−0.003^c^ (−0.057 to 0.052)
Adjusted top score	0.002 (−0.067 to 0.072)	0.016 (−0.054 to 0.085)	−0.013^c^ (−0.082 0.055)
**Secondary outcome**			
NPS^d^			
Average marginal effect, adjusted			
Promoter	−0.024 (−0.098 to 0.054)	0.032 (−0.045 to 0.103)	−0.056 (−0.118 to 0.019)
Detractor	0.007 (−0.018 to 0.032)	−0.009 (−0.032 to 0.014)	0.017 (−0.006 to 0.038)
Patients’ confidence in managing their health			
Average marginal effect, adjusted^e^			
Postintervention score	−0.025 (−0.177 to 0.127)	0.024 (−0.130 to 0.177)	−0.048 (−0.192 to 0.096)
3 mo Postintervention	−0.059 (−0.235 to 0.117)	0.117 (−0.062 to 0.295)	−0.176 (−0.341 to −0.011)

Abbreviations: ASK, AskShareKnow; NPS, net promoter score.

^a^ Arm comparisons expressed as average marginal effects (postintervention vs baseline). The effects are based on the statistical models described for each outcome, and averaged over the entire population using a counterfactual paradigm. For NPS the difference in probabilities compares promoter vs nonpromoter (neutral or detractor) and detractor vs nondetractor (neutral or promoter).

^b^ Mixed-effects logistic regression models were used to estimate the arm comparisons for CollaboRATE top score. The models included fixed effects for study phase, interaction between phase and intervention, health system, and random effects for primary care professionals (PCPs) with an additional random effect for health system 2 PCPs. The adjusted model also included terms for patient and PCP characteristics, visit type, and impact of COVID-19 pandemic (by classifying the visits as occurring up to March 13, 2020, or after this date, when COVID-19 was declared a national emergency). 95% CI computed via 10 000 bootstrap resamples.

^c^ The non-inferiority comparison of hi-tech vs hi-touch was performed at level α = .025 1-sided, with a noninferiority margin of 5%, corresponding approximately to an average marginal effect of 0.05.

^d^ A mixed-effects linear regression model was used to estimate the arm comparisons for NPS. The covariate specification was identical to that of the adjusted primary outcome model, excluding the random effect for health system 2 PCPs. 95% CI computed via 10 000 bootstrap resamples.

^e^ Mixed-effects linear regression models were used to estimate the arm comparisons for confidence. The covariate specification for the postintervention confidence model was identical to that of the adjusted primary outcome model, excluding the covariate for confidence and the random effect for health system 2 PCPs. The model for 3 months postintervention confidence was specified identically to that of the adjusted primary outcome model, excluding the covariate for confidence, the follow-up indicator, the follow-up and arm interaction, and the random effect for health system 2 PCPs.

In the adjusted analysis, better NPS was associated with older age, better patient confidence to take care of own health, visits for acute problems, and visits within family medicine. Furthermore, HS3 had lower NPS than HS1 (eTable 5 in Supplement 3).

### Patient Confidence in Managing Their Health

Patients’ mean (SD) ratings of their confidence in managing their health after the index visit were 8.06 (1.35) for ASK, 8.11 (1.13) for high-tech, and 8.20 (1.04) for high-touch at baseline, and 8.20 (1.08) for ASK, 8.22 (1.09) for high-tech, and 8.23 (1.06) for high-touch at postintervention. Mean (SD) scores decreased for all arms at the 3-month survey to 7.86 (1.27) for ASK, 7.88 (1.28) for high-tech, and 7.93 (1.18) for high-touch (Table 3). After the index visit, no differences were observed between arms. HS2 had significantly lower scores than HS1, with an adjusted mean difference of −0.320 (95% CI, −0.524 to −0.115; *P* < .001). At 3 months, high-tech had a significantly lower score than high-touch (mean difference, −0.176; 95% CI, −0.341 to −0.011; *P* = .04), while no differences were found between high-tech and ASK (mean difference, −0.059; 95% CI, −0.235 to 0.117; *P* = .51) or between high-touch and ASK (mean difference, 0.117; 95% CI, −0.062 to 0.295; *P* = .21) (Table 3; eTable 6 in Supplement 3).

## Discussion

Following a pilot trial that found signals of intervention effect of high-touch compared with usual care,^13^ this trial found no evidence of effectiveness across the 3 arms in the primary outcome. A notable difference between the pilot^13^ and the current study was the use of a professionally designed visit companion booklet mailed to patients rather than an electronic previsit survey. The pilot participants wrote their priorities by hand and handed the booklet to the rooming staff to share with the clinician. Participants in the current study typed their priorities into the EHR patient portal, which were then transmitted automatically to their clinicians in the progress note.^12^ It is plausible that shifting from handwriting important discussion points in a well-designed booklet to typing them could have lessened the depth of cognitive engagement,^26^ thereby reducing the benefits of the high-touch intervention. Nevertheless, after data collection at HS1 ended, the system implemented the previsit questionnaire systemwide based on positive feedback from participants.^12^

Secondary outcomes indicated that high-touch led to higher NPS and greater confidence compared with high-tech. This could be due to the high-touch approach being more intensive and comprehensive, which may have had a stronger impact on NPS than on the patient engagement experience as measured by CollaboRATE. However, the finding regarding NPS is not statistically robust, since the bootstrap CI for the marginal difference in probabilities included the value 0, inconsistent with the significant P value based on asymptotic statistical methods.

Characteristics of patient (age, sex, confidence in managing their own health), clinician (race), visit (telehealth visit vs in-person, before vs after COVID-19), and system were associated with both primary and secondary outcomes, highlighting the importance of adjusting for these potential confounders when evaluating the treatment effects. The importance of local health system implementations must also be considered.

Given numerous constraints in the clinical environment, overcoming these challenges may be difficult. The transition from the pilot study, which showed preliminary results, to this study, which found no evidence of differences across intervention arms in the primary outcome, cautions against assuming that successful pilot interventions will scale effectively in broader practices.

### Limitations

The study’s first limitation may be the primary outcome being not sensitive enough to measure the interventions’ effects. Additional measures for patient-centered communication are needed.^27^ Second, the varying length of time between intervention (eg, SPI practices or use of the mobile app) and index visits could reduce the likelihood of continued adoption of elements of the intervention (eg, joint agenda setting if a patient had multiple issues). Fatigue might have resulted in uneven implementation.^25,28^ Third, potential selection bias may exist regarding who decided to participate in a research project, although such a bias would affect all interventions equally. If specific informed consent is waived for this type of low-risk quality improvement project,^29^ it could reduce selection bias. Furthermore, the inclusion criterion of having appointments scheduled 3 or more days in advance excluded patients with last-minute appointments perhaps for more acute issues. A more targeted delivery of these interventions could have affected outcomes. Specialty settings, where consultations about options for acute conditions are more frequent,^15^ could benefit more from these interventions. Lastly, this study addressed only patient-reported outcomes. Clinician-reported outcomes will be addressed in a future study.

## Conclusions

Our multisite study found no evidence of intervention effects on the primary outcome. Implementing successful pilot interventions into diverse clinical practices is challenging, particularly when minimizing disruptions to workflows. The shift from the harder-to-scale visit companion booklet to the more easily scalable electronic entry of priorities in the patient portal may have contributed to the lack of differential effects. However, the prompt for patients to convey their most important visit topic remains a valuable outcome of the study and is still used at HS1. To better align outcome measures with interventions, alternative patient engagement metrics should be considered. Lastly, health systems could implement these interventions as quality improvement efforts, waiving informed consent to promote universal participation and monitor impact using metrics such as the net promoter score.

## Notes

Supplement 1.
Trial Protocol

Supplement 2.
Statistical Analysis Plan

Supplement 3. **eTable 1.** Key Aspects of 3 Interventions **eFigure.** ASK Arm Poster **eTable 2.** Patient Characteristics by Treatment Arm in Baseline and Post-Intervention Phases **eTable 3.** Unadjusted Comparisons of CollaboRATE Outcomes Between Treatment Arms **eTable 4.** Comparison of CollaboRATE Between Treatment Arms, Adjusting for Health System and Patient and Clinician Characteristics **eTable 5.** Comparisons of Net Promoter Scores (NPS) by Treatment Arm, Adjusting for Covariates **eTable 6.** Comparison of Patient Confidence by Treatment Arm, Adjusting for Health System and for Patient and Clinician Characteristics

Supplement 4.
Data Sharing Statement
