# Supplementary material for: Patient-Clinician Communication Interventions Across Multiple Primary Care Sites: A Cluster Randomized Clinical Trial
Source: JAMA Health Forum. 2024 Dec 13;5(12):e244436. doi: 10.1001/jamahealthforum.2024.4436 (PMC11645648; doi:10.1001/jamahealthforum.2024.4436)
Supplement: Supplement 1. — Trial Protocol [file jamahealthforum-e244436-s001.pdf]

Improving Patient-Centered Communication in Primary Care: A Cluster Randomized Controlled Trial of the Comparative Effectiveness of 3 Interventions (Open and Ask)

National Clinical Trial (NCT) Identified Number: NCT03385512

Principal Investigator: Tai-Seale, Ming

<IND/IDE>Sponsor: PCORI [IHS-1608-35689-1C]

Sponsor means an individual or pharmaceutical or medical device company, governmental agency, academic institution, private organization, or other organization who takes responsibility for and initiates a clinical investigation.

Version Number: v.1.8

<26 June 2018>

All versions should have a version number and a date. Use the international date format (day month year) and write out the month (e.g., 23 June 2015).

Table of Contents

STATEMENT OF COMPLIANCE 1

1 PROTOCOL SUMMARY 1

1.1 Synopsis 1

1.2 Schema 2

2 INTRODUCTION 5

2.1 Study Rationale 5

2.2 Background 5

2.3 Risk/Benefit Assessment 6

2.3.1 Known Potential Risks 6

2.3.2 Known Potential Benefits (Pilot study information) 6

2.3.3 Assessment of Potential Risks and Benefits 7

3 OBJECTIVES AND ENDPOINTS 7

4 STUDY DESIGN 8

4.1 Overall Design 8

4.2 Scientific Rationale for Study Design 10

4.3 Justification for Exposure to Training 10

4.4 End of Study Definition 10

5 STUDY POPULATION 10

5.1 Inclusion Criteria 11

5.2 Exclusion Criteria 11

5.3 Lifestyle Considerations 12

5.4 Screen Failures 12

5.5 Strategies for Recruitment and Retention 12

6 STUDY INTERVENTION 13

6.1 Study Intervention(s) Administration 13

6.1.1 Study Intervention Description 13

6.1.2 Administration of Interventions 16

6.2 Preparation 16

6.2.1 Preparation 16

6.3 Measures to Minimize Bias: Randomization and Blinding 16

6.4 Study Intervention Compliance 17

6.5 Prior Training 17

6.6 Schedule of Activities (SoA) 17

7 STUDY INTERVENTION DISCONTINUATION AND PARTICIPANT DISCONTINUATION/WITHDRAWAL 18

7.1 Discontinuation of Study Intervention 18

7.2 Participant Discontinuation/Withdrawal from the Study 18

7.3 Lost to Follow-Up 18

8 STUDY ASSESSMENTS AND PROCEDURES 18
8.1 Efficacy Assessments 18
8.2 Safety and Other Assessments 21
8.3 Unanticipated Problems 22
8.3.1 Definition of Unanticipated Problems (UP) 22
8.3.2 Unanticipated Problem Reporting 22
8.3.3 Reporting Unanticipated Problems to Participants 22
9 Statistical Considerations 22
9.1 Statistical Hypotheses 22
9.2 Sample Size Determination 2
9.3 Populations for Analyses 5
9.4 Statistical Analyses 5
9.4.1 General Approach 5
Data Preparation Prior to analysis, the data files will be checked for out of range, otherwise invalid values, and outliers. Following standard research practice, we will perform preliminary analyses required for variable construction, checking for the validity of missing at random assumption and perform multiple imputations of missing data, as needed. We will also assess psychometric properties of study variables (e.g., calculate Cronbach's alpha) before analyses of intervention effects. Data set documentation will then be developed and distributed to the study team. This documentation will include descriptions of variables, algorithms for calculated variables and variable distributions and relevant references. Non-response rates will be calculated and monitored at each stage of data collection. 5
9.4.2 Analysis of the Primary Endpoint(s) 6
9.4.3 Analysis of the Secondary Endpoint(s) 8
9.4.4 Baseline Descriptive Statistics 10
9.4.5 Sub-Group Analyses 10
9.4.6 Tabulation of Individual participant Data 10
9.4.7 Exploratory Analyses 10
10 SUPPORTING DOCUMENTATION AND OPERATIONAL CONSIDERATIONS 11
10.1 Regulatory, Ethical, and Study Oversight Considerations 11
10.1.1 Informed Consent Process 11
10.1.2 Study Discontinuation and Closure 12
10.1.3 Confidentiality and Privacy 12
10.1.4 Future Use of Stored Data 13
10.1.5 Key Roles and Study Governance 14
10.1.6 Safety Oversight 21
10.1.7 Clinical Monitoring 22
10.1.8 Quality Assurance and Quality Control 22
10.1.9 Data Handling and Record Keeping 22
10.1.10 Protocol Deviations 22
10.1.11 Publication and Data Sharing Policy 23
10.1.12 Conflict of Interest Policy 24

10.2 Additional Considerations 24
10.3 Abbreviations 25
10.4 Protocol Amendment History 26
11 REFERENCES 27

STATEMENT OF COMPLIANCE

The trial is carried out in accordance with International Conference on Harmonisation Good Clinical Practice (ICH GCP) and the following:

- United States (US) Code of Federal Regulations (CFR) applicable to clinical studies (45 CFR Part 46, 21 CFR Part 50, 21 CFR Part 56, 21 CFR Part 312, and/or 21 CFR Part 812)

National Institutes of Health (NIH)-funded investigators and clinical trial site staff who are responsible for the conduct, management, or oversight of NIH-funded clinical trials have completed Human Subjects Protection and ICH GCP Training.

The protocol, informed consent form(s), recruitment materials, and all participant materials have been submitted to the Institutional Review Board (IRB) for review and approval. Approval of both the protocol and the consent form has been obtained. Any amendment to the protocol will require review and approval by the IRB before the changes are implemented to the study. In addition, all changes to the consent form will be IRB-approved; a determination will be made regarding whether a new consent needs to be obtained from participants who provided consent, using a previously approved consent form.

1 PROTOCOL SUMMARY

No text is to be entered in this section; rather it should be included under the relevant subheadings below.

1.1 SYNOPSIS

Title: Improving Patient-Centered Communication Primary Care: A Cluster Randomized Controlled Trial of the Comparative Effectiveness of Three Interventions

Study Description: This is a large scale multi-center cluster randomized controlled trial (RCT) to assess the comparative effectiveness of three interventions in diverse settings and populations. Findings will help healthcare systems decide which approach to adopt to empower patients and enable providers to engage in patient centered communication.

Objectives:

1) Engage with patients and healthcare providers who have used the tools in the pilot study at the Sutter Health Palo Alto Medical Foundation (PAMF), as well as new stakeholders at University of California San Diego Health System and University of Massachusetts and Reliant Medical Group, to further refine and adapt these patient-centered interventions to be integrated into real world primary care clinics.
2) Conduct a large scale cluster RCT with three arms, to evaluate the comparative effectiveness of three interventions: OPEN with in-person SPI training (High Touch), OPEN with mobile application training (High Tech), and ASK. Primary outcomes will include patient perceptions of how well their PCPs have engaged them in

patient centered communication. We will also measure patients' confidence in managing their health, their intention to follow through with care plans, and downstream behaviors in following through with care plans, use of services such as phone calls, secure messaging, and additional visits.

3) Identify the strategy that has the most potential for sustained impact and replication within and across healthcare systems. We will analyze the fidelity to the intervention protocols, including consistency of delivery as intended and the time/effort involved in implementing the interventions. We will also assess the extent to which the programs become institutionalized.

Endpoints:

1) Patient reported experience with care
2) Patient reported confidence and intention to adhere to care plan
3) Clinical indicators
4) Post-visit service use and other system impact measures
5) physician and clinic staff experience with care
6) Time and effort involved in implementing the interventions

Study Population: *A sample of 21 cluster randomized clinics across three sites (UC San Diego Health, Reliant Medical Group, Sutter Health) in which about 5 primary care providers will be recruited from each clinic (n = 105). For each physician 10 patients will complete baseline surveys (n = 1050) and then after the intervention is administered 40 patients per physician will be surveyed (n = 4200).*

Description of Sites/Facilities Enrolling Participants: *The three sites that will enroll participating clinics/ physicians/patients will be UC San Diego Health in San Diego, Sutter Health in San Francisco Bay Area and Reliant Medical Group in Worcester, Massachusetts. There will be no recruitment from outside the United States.*

Description of Study Intervention: *Open High Touch, Open High Tech and AskShareNow (ASK) are the three interventions. Open High Touch and Open High Tech include the same information through different delivery mechanism. Physicians will either practice patient centered communication skills with a Standardized Patient Instructor or through an application (APP) on their mobile phones. The ASK arm uses a poster placed on the wall of the exam room to encourage patients to ask their physicians three questions.*

Study Duration: *Sept 2017 -November 2021 .*

Participant Duration: *Patient baseline survey participants will respond to one survey. Intervention phase patient participants will have a survey at their initial visit and then a follow-up surveys at 3 months post-visit.*

1.2 SCHEMA

Figure 1. Overview of Cluster Randomized Trial

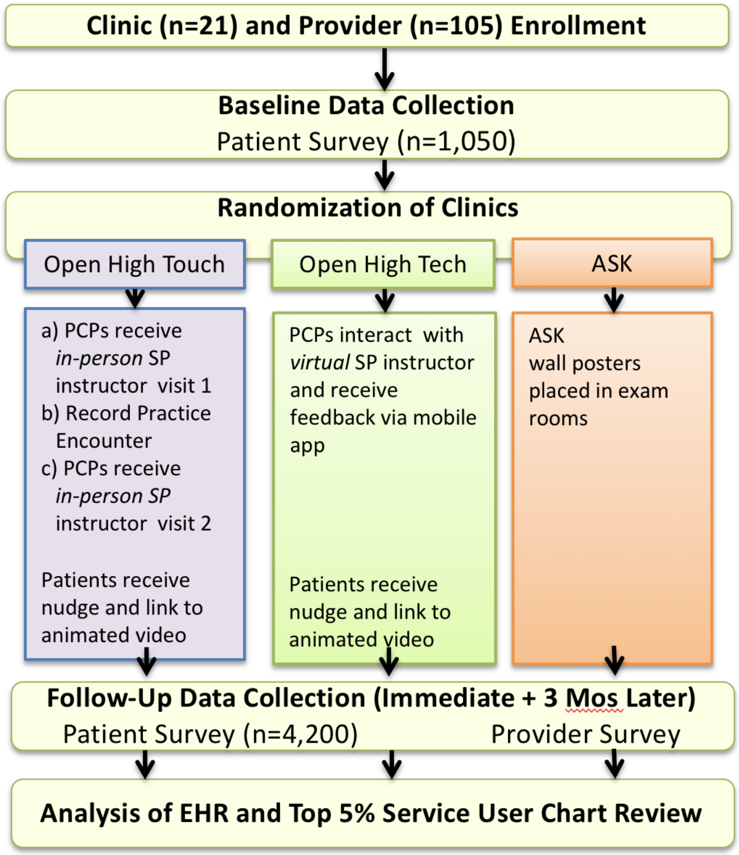

Note: Open: Open Communication Intervention, ASK: AskShareKnow,

SP: Standardized Patient,

PCP: primary care provider, include nurse practitioner, physician's assistant

2 INTRODUCTION

The following subsections should include relevant background information and rationale for the clinical trial. This should be a brief overview (e.g., approximately 3-7 pages). Referring to the Investigator's Brochure (IB) for more detail is also appropriate.

2.1 STUDY RATIONALE

Evidence from research on clinical consultations indicates that patients are frequently neither heard nor informed by their healthcare providers of the full range of clinical assessment or treatment options available and the potential associated outcomes.[1,2] Previous studies have shown that patients are often afraid to ask questions or disagree with a recommendation from a healthcare provider for fear that they would be viewed as "difficult" and as a result would receive lower quality care in the future.[3,4]

Patient's encounters with their providers are at the center of their healthcare. The way in which clinicians communicate with patients can affect patients' understanding of their health condition[5], health status[6,7], and quality of care[8-10]. Patient-centered communication and shared decision making are also ethically the right things to do. Systematic reviews of the preconditions for improving healthcare delivery have emphasized the importance of patient-physician communication as a mediator and moderator of healthcare quality.[11.] It has been well documented, however, that patients often hesitate in being completely open about their concerns and preferences during clinical encounters out of fear of being labeled "difficult"[3,12] by their physicians. In addition, even when patients do ask questions, physicians' answers vary in quality.[13] When patients are activated to ask questions and be more engaged in their visits, some are met with discouraging or dismissive reactions from unprepared clinicians.[14]

Numerous efforts have been extended to improve patient-provider communication. Rarely has there been a direct comparison of multiple efforts, however, that aims at assessing the comparative effectiveness of different interventions. In the wake of the Affordable Care Act, healthcare systems are looking for an efficient, scalable approach to empowering patients to speak up and preparing physicians to respond effectively when they do.

2.2 BACKGROUND

We developed an intervention with stakeholders and conducted a pilot study. Specifically, our pilot study followed the best practice of iteratively co-designing an intervention with stakeholders and developed a multidimensional intervention which we call "OPEN" (for open communication) and compared it with the existing AskShareKnow(ASK)[16] intervention in cluster randomized controlled trial (RT).

Healthcare is moving toward more patient centered models. Financial and organizational incentives are being more aligned with patient reported outcomes. Patients care a great deal about being able to communicate openly with their providers. Yet, our work found that patients still often feel reluctant to express their preferences, for fear of being labeled difficult.[3,4] This was the problem we set out to solve with our PCORI-funded pilot study, "Creating a Zone of Openness to Increase Patient-Centered Care" (1IP2PI000055), (*https://www.youtube.com/watch?v=16lvLkO14bo*.)

A manuscript from the study has been accepted for publication in the April 2016 issue of Health Affairs.[15]

2.3 RISK/BENEFIT ASSESSMENT

2.3.1 KNOWN POTENTIAL RISKS

There is a slight risk that research data files might be compromised or obtained or viewed by unauthorized persons. Our procedures for protecting against such risks are described in “Methods of Data Analysis” section.

There is also a risk that the patient may feel uncomfortable answering some of the questions in the surveys. We will inform all patients in the informed consent that they do not have to answer any questions that they do not want to.

There is a potential risk of boredom or fatigue associated with completing surveys. We will design the surveys to only include essential questions and to use user-friendly survey designs in RedCap.

There is risk associated with those patients who will have their visit audio-recorded in the Open High Touch arm. These visits will be recorded on encrypted audio recorders and then stored in a secure folder on the UCSD server. The 2 standardized patient instructors will access the recordings in our office space. They will be trained to not use any protected health information (PHI) in their feedback/discussion with the physician about the visit. A study ID will be assigned to each of these participants to be used as a reference for correspondence. In addition, we will also convey in the informed consent document that the patient participant has the right to withdraw at any time and that they may also chose to withdraw their recording immediately following their visit if any sensitive issues arise during their visit that they do not feel comfortable sharing.

2.3.2 KNOWN POTENTIAL BENEFITS (PILOT STUDY INFORMATION)

In the pilot study which showed great promise, OPEN and ASK both showed efficacy in producing better patient-reported experience (measured by the CollaboRATE scores[23] and the Doctor Facilitation subscale of the Perceived Involvement in Care scale)[24] than usual care. (Figure 3)

The strengths of our evaluation approach included asking patients about their experience with specific aspects of patient-physician interaction – the aspect of care for which patient-reported measures are most credible – and collecting the information immediately after their visits, therefore avoiding recall bias.[25]

Narrative feedback from patient and provider participants suggested that while the OPEN intervention led to greater patient and provider engagement, the ASK intervention was minimally disruptive of clinical workflow. Because both proved to be efficacious in this small pilot, more information from a greater variety of practice settings is needed to determine which intervention is most practical and also scalable for healthcare systems.

Figure 3. % Of Patients Gave All Top Scores (Unadjusted)

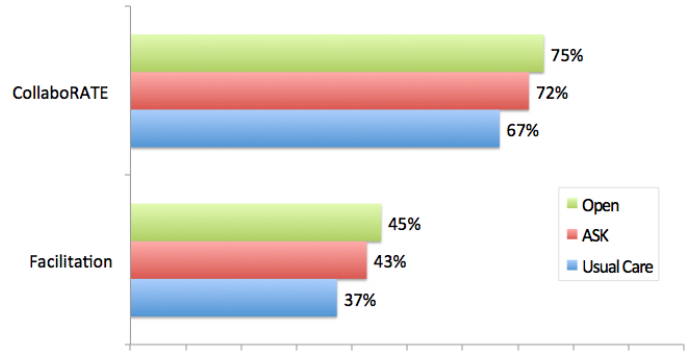

The improvement of this study upon the previous is that there will be direct comparison between the arms as well as introducing the Open High Tech arm in an effort to increase scalability of the intervention.

2.3.3 ASSESSMENT OF POTENTIAL RISKS AND BENEFITS

The potential benefits outweigh the risks.

3 OBJECTIVES AND ENDPOINTS

OBJECTIVES	ENDPOINTS	JUSTIFICATION FOR ENDPOINTS
Primary		
Evaluate the comparative effectiveness of three interventions: OPEN High Touch, Open High Tech and ASK at improving patient perceptions of PCPs engagement in patient-centered communication.	Proportion of patients in each arm on CollaboRATE survey giving the maximum score compared between arms using the hierarchical approach to testing found in section 9.<br><br>Facilitate scores will be compared using the proportion of patients giving the maximum score using the hierarchical approach to testing the arms.	Allows for testing for non-inferiority between Open High Tech and Open High Touch as well as superiority compared to ASK arm when testing patient experience with care.
Secondary		

OBJECTIVES	ENDPOINTS	JUSTIFICATION FOR ENDPOINTS
Test confidence of patients in their ability to adhere to the plan that they and their provider decided upon immediately post appointment versus 3 months after<br><br>Identify the strategy that has the most potential for sustainable impact and replication within and across healthcare systems. Also, the fidelity to the intervention protocols.	Does the confidence question mean change over time differently for the three arms.<br><br>Time effort involved in implementing the interventions	
Tertiary/Exploratory		
Sensitivity Analysis		

4 STUDY DESIGN

4.1 OVERALL DESIGN

Phase 1: Phase 1 includes intervention and survey development work to take place prior to the RCT. The tasks include development of the following 6 components which we describe in further detail below: (1) OPEN High Touch; (2) OPEN High Tech; (3) ASK posters; (4) Online messaging content and system; (5) survey instruments; (6) English to Spanish translation of our documents which will take place at UCSD.

OPEN High Touch intervention - The **High Touch** intervention will be modeled after the Open Communication intervention developed in the pilot which contained three components: (a) a one question pre-visit survey delivered through the patient portal of the EHR, asking patients what they most want to discuss with their physician in the upcoming visit; (b) an animated video for patients providing coaching on how to best prepare for their upcoming visits and get the most from the visits; (See http://bcove.me/fevffx4w for the version used in the pilot; and (c) Standardized Patient Instructor (SPI) providing communication coaching for physicians on how to incorporate what matter most to patients in the visit, with empathy, and clarity. We have adapted the (1) the existing paper version of the Visit Companion Booklet to be a one item questionnaire (see attached) and (2) the existing in-person SPI training of physicians and medical assistants to reflect revised workflow with the previsit questionnaire and after visit

summary (AVS), and (3) updated the video explaining the OPEN intervention to participants to illustrate these new workflows. We have also revised the standardized patient instructor manual developed in the pilot study.

OPEN High Tech intervention - For the **High Tech** arm, the patient components of the intervention will be identical to the patient components of the High Touch arm (i.e., the pre-visit survey and patient coaching video). The difference will be in the PCP training: we will replace the in-person SPI with a mobile app with embedded audio and video vignettes demonstrating the communication challenges (e.g., patient with a big list of issues, patients who resist physician recommendations, and patients who disagree with physician) and recommended strategies.[13] A mobile app offers several advantages, including being accessible at a convenient time for busy providers, being easily disseminated, and easily updated. The app will be interactive, posing questions to learners in association with video vignettes and asking learners to answer how they would handle the situation. We will start with the idea of building a set of short mobile modules that mirror the High Touch approach, honing skills on acknowledging patient's agenda, negotiate a joint agenda, invite patient to teachback and incorporate it in the After Visit Summary in the EHR. This is currently under development at UCSD.

ASK intervention - The **ASK** intervention is intended to activate patients by encouraging them to ask three questions during their primary care visit: (1) What are my options? (2) What are the possible benefits and risks of each option? (3) How likely are each of the benefits and risks to happen to me? These questions are printed on two types of posters with identical text but different graphics and placed in exam rooms used by providers in clinics randomized to the ASK arm of the trial.

Phase 2: Phase 2 covers the trial recruitment, and three waves of data collection. The diagram in Figure 2 entitled "Summary of Patient-Centric Outcome Measures" (uploaded with this protocol) illustrates the overall structure of patient data collection. Prior to the start of the RCT we will collect baseline (T0) data to allow measurement of primary care provider (PCP) performance prior to the trial. Patients participating in the T0 phase will provide only post-visit ratings of their encounters; we will not collect other outcome data or clinical indicators for these patients. For all patients in the intervention phase, we will be collecting information at two time points: 1) immediately post-encounter (T1); and 2) three months post-encounter (T2). We will further sample the top 5% high users of services after the intervention and review their medical records including the indexed visit and subsequent services that had occurred within four weeks after the indexed visit. The chart review will enable us to decipher the reasons for high volume of services after the indexed visit.

It should also be noted that the sub group of patients that have diabetes or hypertension will be studied in more detail in sub group analyses described further in Section 9.4.7

4.2 SCIENTIFIC RATIONALE FOR STUDY DESIGN

This is a multi-center randomized study that is randomized at the clinic level and has clusters of physicians in clinics and patients per physician. As rationale for the sample size, analyses of the pilot study data revealed that the estimated within-PCP intra-class correlation (ICC) between baseline and follow-up outcomes was negligible (ICC=0.000004 for CollaboRATE=9, ICC=0.00000005 for Facilitate=9). In the absence of within-PCP ICC, the baseline samples do not help reduce variability, making the statistical analysis just as effective as the direct comparison of the follow-up time points. In other words, the baseline samples have no statistical value if ICC is again negligible. This is confirmed in our statistical simulations. However, if in our study it turns out that the within-PCP ICC is positive (but small), the baseline samples will improve power. For this reason, we use 10 baseline and 40 post-intervention patients.

4.3 JUSTIFICATION FOR EXPOSURE TO TRAINING

Every effort was made to make the exposure to training for the providers the same in both Open arms. The exposure to training is similar however the feedback will be more thorough for the Open High Touch arm in which the SPIs give real-time in-person and written feedback to the providers at the end of the visit. In the app for Open High Tech, there will be no such feedback from a person. Providers assess their own performance in the app after they complete each module.

This difference in exposure and training between the two Open arms is made to examine if the app offers an non-inferior intervention. It would be more scalable than the High Touch intervention.

The Ask arm will not have feedback as the intervention is just the poster in the exam room. For this reason, the exposure to training is only contained in the poster which prompts providers and patients to engage in shared decision making.

4.4 END OF STUDY DEFINITION

A participant is considered to have completed the study if he or she has completed all phases of the study, the initial physician visit and survey follow-ups at 3 months after the visit.

5 STUDY POPULATION

5.1 INCLUSION CRITERIA

In order to be eligible to participate in this study, a patient must meet all of the following criteria:

1. Provision of signed and dated informed consent form;
2. Stated willingness to comply with all study procedures and availability for the duration of the study;
3. Male or female, aged 18 years or older;
4. Patients must see a PCP at a participating clinic site (UC San Diego Health, Palo Alto Medical Foundation, or Reliant Medical Group);
5. Patients must have an upcoming primary care appointment and provide online consent 3-7 days before the appointment if assigned to the Open High Touch or High Tech arms, or within 3 days of the indexed visit if assigned to the Ask arm;
6. Must speak and read English or Spanish;
7. Patients must have an activated patient portal account (eg. MyUCSDChart at UCSD, MyHealthOnline at PAMF, and MyChart at Reliant);
8. Patients with all health conditions may participate in this study;
9. Return the post-visit survey within 7 days of the indexed visit.

In order to be eligible to participate in this study, a Primary Care Provider must meet the following criteria:

1. An internal medicine or family medicine PCP at a participating clinic site;
2. Must have scheduled appointment during the study period;
3. Consent to participate in the study.

In order to be eligible to participate in this study, a Medical Assistant (MA) or nurse must meet the following criteria:

1. Any medical assistant or nurse working with a participating PCP at a participating clinic site
2. Consent to participate in the study.

5.2 EXCLUSION CRITERIA

Providers will be excluded from the study if they are:

1. Stakeholders and Research Team members

This study aims to be as inclusive as possible there are only 3 excluded groups. An individual who meets any of the following criteria will be excluded from participation in this study:

1. A patient does not speak English or Spanish
2. Patients without activated patient portal accounts
3. Patient does not consent at least 3 days in advance of appointment if assigned to the Open arms or within 7 days of the indexed visit if assigned to the Ask arm

5.3 LIFESTYLE CONSIDERATIONS

Not Applicable.

5.4 SCREEN FAILURES

A participant can only fail to screen if they have an appointment within the next three days from informed consent.

5.5 STRATEGIES FOR RECRUITMENT AND RETENTION

The researchers are embedded in these healthcare systems with cooperative relationships with the host systems. We have excellent track record in enrolling large number of subjects in studies.

Across the three recruitment sites, PCPs see an average of 6 patients each day who meet our eligibility criteria. Using our intended sample of 110 PCPs that means across all sites 660 patients/day will meet our eligibility criteria. These numbers were determined through retrieval of EHR data for patients/appointments meeting these criteria during the 2015 calendar year. We will screen patients via data extraction from EHR to make sure they meet all criteria (are 18 years or older, have upcoming appointments, speak English or Spanish, with an activated MyChart account.) The eligible population, number expected to be screened, and total expected to be eligible after screening will be constant. The only attrition we expect to see after the initial invitation sent to patients via the patient portal will be based on patient availability and willingness to participate in our study. We expect a participation rate of approximately 20%. This rate was approximated by taking into consideration a 10% participation rate achieved during the pilot study which required much more time and effort placed on patients. For example, in the pilot study patients needed to speak via phone with a research team member prior to their appointment, and meet the researcher at the clinic at least 20 minutes prior to their appointment time. Given the reduced burden placed on patient in this proposed project, and the transfer of materials to the online/messaging format, we estimate a 20% participation rate. Using the 20% participation rate we calculated the number needed to screen in order achieve our target enrollment of 5,250 (1,050 patients taking the baseline survey and 4,200 patients taking the post-RCT follow-up survey).

We plan to stagger recruitment in two different waves. Baseline patient surveys will be collected over 8 months and can happen simultaneously at all clinics (months 13-20). Post-intervention follow-up patient surveys will be collected in the subsequent 12 months (months 21-33). We also plan to stagger data collection between clinics (some clinics may begin earlier and end earlier) and within clinics (we will collect data on certain days of the week each month to maximize variability in responses). With this approach we estimate we will recruit a total of 262.5 patients per month (across all three sites).

We do expect some challenges to enrollment. Patients may be unlikely to read or respond to emails that do not directly pertain to their individual medical care. They may not read the initial message asking them to prepare for their upcoming visit (and for patients in the Open Communication

arm – to view a video online). They may be unable, unwilling, or forget to take the survey after receiving a message about it following their appointment. We have taken these expected obstacles into account in our 20% participation rate estimate. There may be patients who partially complete the survey, but we intend to keep the survey as brief as possible to minimize this risk. We will also be able to use partial survey data in certain instances (e.g., if they fully complete one of the measures).

6 STUDY INTERVENTION

6.1 STUDY INTERVENTION(S) ADMINISTRATION

6.1.1 STUDY INTERVENTION DESCRIPTION

As shown in Figure 1 above, two Open Communication intervention options (High Touch and High Tech) and ASK will be compared in this 3-arm comparative effectiveness study. This is a multi-level, system-based clustered RCT. Seven clinics will be randomized into each arm. We will use baseline survey data to ensure balanced assignment of clinics.

1) OPEN High Touch – This arm will be modeled after the Open intervention developed in the pilot which contained three components: (a) a pre-visit survey delivered through the patient portal of the EHR, asking patients what they most want to discuss with their physician in the upcoming visit; (b) an animated video for patients providing coaching on how to best prepare for their upcoming visits and get the most from the visits; (See http://bcove.me/fevffx4w for the version used in the pilot study); and (c) Standardized Patient Instructor (SPI) providing communication coaching for physicians on how to incorporate what matter most to patients in the visit, with empathy, and clarity. (Figure 3 earlier.)

2) OPEN High Tech – In order to enhance scalability, we will develop this additional arm. The patient components of the intervention will be identical to the patient components of the High Touch arm (i.e., the pre-visit survey and patient coaching video). The difference will be in the PCP training: we will replace the in-person SPI with a mobile app program with embedded audio and video vignettes demonstrating the communication challenges (e.g., patient with a big list of issues, patients who resist physician recommendations, and patients who disagree with physician) and recommended strategies.[31] A mobile app program offers several advantages, including being accessible at a convenient time for busy providers, being easily disseminated, and easily updated. The mobile app will be highly interactive, posing questions to learners in association with video vignettes and asking learners to type or speak into the app their answers and exactly what they would say to a patient in a given situation. We will start with the idea of building several 10-minute modules that mirror the High Touch approach, honing skills on acknowledging patient’s agenda, negotiating a joint agenda, showing empathy, discussing options, engaging in shared decision making, inviting patient to teachback and incorporate it in the After Visit Summary in the EHR. We have chosen a technology company in the San Francisco Bay Area that had worked with us to build an online tool to help seniors find the best Medicare

Part D coverage plans. That study is funded by PCORI as well. The details of the program will be developed fully with iterative input from stakeholders during the first year of the study.

We plan to work with our stakeholders from American Board of Internal Medicine (ABIM) and American Academy of Family Physicians (AAFP) to ensure that the content in our intervention will be consistent with their respective Maintenance of Certification requirements.

We will need to do usability testing and refine the program before we can launch it. We have the support of the Chief Medical Officer, Dr. Mat Hernandez at the Palo Alto Foundation Medical Group who is teaching a communication class for all physicians at Sutter Health). We are engaging stakeholders at UCSD and Reliant as well so that the program will be acceptable to clinicians in all three systems. We are confident that we will have a product that people will use. It will be necessary to use the first year to put this program online, perform usability testing, refine it, and iterate until we have a minimally viable product. Then launch it at UCSD, Sutter Health/PAMF, and Reliant.

3) ASK – The ASK arm is intended to activate patients by encouraging them to ask three questions during their primary care visit: (1) What are my options? (2) What are the possible benefits and risks of each option? (3) How likely are the benefits and risks of each option to occur to me? The intervention involves placing posters with these questions in all exam rooms used by providers in clinics randomized to the ASK arm of the trial. Note that because of our use of a cluster randomized design, we will avoid "contamination", that is, providers in the HIGH TOUCH and HIGH TECH arms will not see patients in the clinics assigned to the ASK arm.

Figure 3 summarizes the key aspects of the 3-arm CER proposed. Merged cells in the table illustrate similar content whereas individual cells demonstrate what is unique to each study arm.

Figure 4. Key Aspects of 3 Interventions

Open HIGH TOUCH	Open HIGH TECH	ASK
**Patient**		
**EHR Patient Portal-Based Appointment Reminder to Patient**<br>• Link to single item questionnaire: "What do you most want to discuss at your upcoming visit?"<br>• Invitation to participate in study<br>• Link to online informed consent and enrollment portal		• Invitation to participate in study<br>• Link to online informed consent and enrollment portal
• Link to Animated Video for Patient to Watch before Visit- encourages patients to:<br>• Share concerns and indicate which is most important<br>• Teachback next steps to physician		Patient sees poster with 3 ASK questions in the exam room. (See

<table>
<tr><td colspan="2"></td><td>Section B1.b for exact wording of the questions.)</td></tr>
<tr><td colspan="3">Portal-based Post-Visit Questionnaire to Patient
• Items assessing patient’s perception of the encounter
• Items assessing patient’s understanding of next steps and intent to complete next steps</td></tr>
<tr><td colspan="3">Medical Assistant (MA)/Nurse</td></tr>
<tr><td colspan="2">MA/Nurse Engages Patient at Visit
• Reviews most important agenda item, annotates if needed
• Ensures prominence of item for PCP review</td><td>MA/Nurse Performs Standard Rooming
May/may not highlight patient’s most important issue</td></tr>
<tr><td colspan="3">Primary Care Provider (PCP)</td></tr>
<tr><td>In–Person Standardized Patient Instructor (SPI) for PCP
Visit 1: 30-minute session with SPI for Introduction, Instruction, and Roleplay to:
- Understand the patient’s top concern
- Acknowledge Patient Agenda
- Negotiate Joint Agenda
- Shared decision making
- Teachback from Patient
- Incorporate next steps into After Visit Summary
PCP records actual “practice patient” encounter – gives to SPI
Visit 2: 30-minute session with SPI for feedback on practice patient, further instruction on skills and role play</td><td>Mobile App Standardized Patient Instructor (SPI) Training for PCP
Multiple brief mobile virtual coaching sessions to teach and practice:
- Understand the patient’s top concern
- Acknowledge Patient Agenda
- Negotiate Joint Agenda
- Shared decision making
- Teachback from Patient
- Incorporate next steps into After Visit Summary</td><td>PCP sees poster with 3 ASK questions in exam room. (See Section B1.b for exact wording of the questions.)</td></tr>
<tr><td colspan="3">Patient and PCP Encounter</td></tr>
<tr><td colspan="2">• PCP reviews most important agenda item
• Patient &PCP discuss most important item
• Patient and PCP discuss additional issues
• Patient & PCP engage in Shared Decision Making (SDM) and jointly agree on next steps
• PCP prompts patient to “teachback” to check agreement and understanding
• After-visit summary (AVS) that documents the care plan is created in EHR</td><td>PCP may / may not review most important agenda item
• Patient and PCP discuss issues
• Patient or PCP may/may not pose ASK questions to facilitate SDM</td></tr>
</table>

• Reiterate next steps in AVS before it is handed to patient	• After-visit summary is created in EHR<br>• Next steps in AVS may or may not be reiterated before it is handed to patient
**Research Team**	
**Electronic Data Extraction**<br><br>EHR data extracted: number and timing of post-visit telephone calls, emails and follow up visits	
**In-depth EHR Review**<br><br>Chart review on subsample of high utilization patients<br><br>Research nurse reviews EHR to determine reasons for calls, emails, visits, and link between utilization and most important concern	

6.1.2 ADMINISTRATION OF INTERVENTIONS

Information on the administration of the interventions is included in Figure 4. Specifically, for the Open arms, the physician and MA will receive training prior to the visit of any patients either through SPIs or (standardized patient instructor) or through mobile virtual coaching through an application. For the ASK arm physicians will only view the ASK poster with 3 ASK questions in the exam room and the MA will perform standard rooming.

6.2 PREPARATION

6.2.1 PREPARATION

SPIs are being trained by an SPI trainer via telephone sessions, a two-day training session, and a manual that was developed by the research team.

The bilingual posters were created by the research team and translated by a certified translator.

The high-tech app is being built by developers and the content for the app was developed by the research team and informed through stakeholder meetings.

6.3 MEASURES TO MINIMIZE BIAS: RANDOMIZATION AND BLINDING

This trial will be randomized at the clinic level to not bias any results by having two intervention present within any single clinic.

There are two types of blinding that help minimize the bias present in this study. First, the recruiters that are attempting to recruit providers from clinics will not be aware of which arm each clinic was randomized to. Potential providers will not aware of which intervention they are in until after they have consented into the trial.

6.4 STUDY INTERVENTION COMPLIANCE

We will measure patient and PCP use of our interventions to the extent they can be captured in the EHR, to inform the exposure level of interventions. They include patient responses to previsit patient survey that serves as a nudge about informing providers about what is most important for the visit to address, access to the animated video, PCP visits with in-person SPI, PCP access to virtual SPI in the two OPEN arms. We will extract data on access to AVS in the EHR made by all patients of participating PCPs in each arm. This information can offer contextual information that can help us interpret the intent to treat analysis results.

6.5 PRIOR TRAINING

If a provider has received prior communication training or is currently enrolled in separate training, they will be asked to disclose that in the end of study physician survey with the question, “Have you had any training in patient communication or similar topics in the past year?”. This will be used as control in any models during analysis.

6.6 SCHEDULE OF ACTIVITIES (SOA)

Figure 2: Summary of Patient-Centric Outcome Measures for Comparative Effectiveness Evaluation

Outcome Type	Measure	T1 Immediate post-encounter	T2 3 months post-encounter	T3 12 months post-encounter	Data Source
Patient reported experience with care	CollaboRATE[23]	X			Patient survey
	Facilitation[24]	X			Patient survey
Action plan, patient reported confidence/intention to adhere and adherence	Action plan<br>CONFIDENCE[39]<br>INTENTION[34,35] to adhere<br>Adherence to action plans	X<br>X<br>X	 X<br>X<br>X		Patient survey
Clinical indicators	VR12<br>Blood pressure<br>HbA1c<br>LDL	X<br>X<br>X<br>X	X<br>X<br>X<br>X	 X<br>X<br>X	Patient survey<br>EHR<br>EHR<br>EHR

Service use, impact on healthcare system	Patient-initiated calls, e-messages, office visits after indexed visits			X	EHR – structured fields, access log, Physician Efficiency Profile, and chart review of top 5% users in the 12 months after the indexed visit.

7 STUDY INTERVENTION DISCONTINUATION AND PARTICIPANT DISCONTINUATION/WITHDRAWAL

7.1 DISCONTINUATION OF STUDY INTERVENTION

A physician can discontinue the study intervention by not completing the High Tech or High Touch training. A patient can discontinue the study intervention by not completing the post-visit or 3 month follow-up survey.

7.2 PARTICIPANT DISCONTINUATION/WITHDRAWAL FROM THE STUDY

Participants are free to withdraw from participation in the study at any time upon request or by not completing the post-visit or 3 month follow-up survey. This makes the record of reasons for such withdrawal more difficult. Therefore, a reason for discontinuation or withdrawal may not be recorded.

7.3 LOST TO FOLLOW-UP

A participant will be considered lost to follow-up if he or she fails to complete the follow-up survey 3 months after visit.

8 STUDY ASSESSMENTS AND PROCEDURES

8.1 EFFICACY ASSESSMENTS

A. Outcomes that people representing the population of interest notice and care about (RQ-6)

Most of the data collection instruments used in this study have been developed and tested, although some modifications will be made for this study, based on input from new stakeholder panels and team members for the proposed study. In Figure 4, we summarize the outcomes we will examine and the corresponding data sources. Outcomes in A1-A3 below are for **Aim 2**, whereas those covered in A4 are for **Aim 3.**

Patients participating in the baseline phase will provide only post-visit ratings of their encounters; we will not collect other outcome data or clinical indicators for these patients. For all patients in the intervention phase, we will be collecting information at three time points: 1) immediately post-encounter; 2) three months post-encounter; and 3) 12 months post-encounter. Further details on the data to be collected at each time point are provided in Figure 4.

A1 Patient reported experience with care: two measures that were selected with patient stakeholder input in the pilot study will be used. These validated scales are patient-reported outcomes (PRO) when patients are the best source of information. (PC-3) (IR-4) The first is CollaboRATE,[23] a validated 3-item patient engagement measure that captures patient perceptions of communication and decision-making during the appointment and is measured by the percentage of patient participants who gave the highest possible score. The 3 questions are: "How much effort was made to help you understand your health issues?" "How much effort was made to listen to the things that matter most to you about your health issues?" "How much effort was made to include what matters most to you in choosing what to do next?" The second PRO is the Physician Facilitation subscale of the Perceptions of Involvement in Care questionnaire.[24] The Facilitation subscale is a 5-item validated and reliable measure of patient perceptions of how well the physician facilitated their involvement in decision making. The questions are: my doctor (1) asked me whether I agree with his/her decisions; (2) gave me a complete explanation for my medical symptoms or treatment; (3) asked me what I believe is causing my medical symptoms; (4) encouraged me to talk about personal concerns related to my medical symptoms; (5) encouraged me to give my opinion about my medical treatment. A scale of 0 "Definitely Disagree" to 9 "Definitely Agree" will be used.

A2 Patient reported confidence and intention to adhere to care plan*:* Patient reported confidence will be measured by the question "Overall, how confident are you about your ability to take good care of your health?" using a 5-point Likert scale: 5=Completely confident, 4=very confident, 3=somewhat confident, 2=a little confident, 1=not confident at all." This is a vetted question used in the Health Information National Trends Survey (HINTS), a biennial, cross-sectional survey of a nationally-representative sample of American adults, developed and used by the Center for Disease Control and Prevention and National Cancer Institute (*https://www.healthypeople.gov/2020/data-source/health-information-national-trends-survey*). In an ongoing study at PAMF, we fielded this question (CONFIDENCE) along with the Patient Activation Measure (PAM)[32] to examine the correlation between the two approaches. Confirmatory factor analysis of PAM yielded a Cronbach's alpha of 0.90. The correlation between the PAM factor score and CONFIDENCE is 0.71. Since CONFIDENCE is in the public domain therefore more scalable, is highly correlated with PAM, and is much easier to measure (a single item), we will use it to measure patient's confidence in managing their health.

We will use the theory of planned behavior[33] as a foundation for assessing patient intention to follow through with the next steps or care plan discussed with their PCP. We have developed measures based on the recommendation of Azjen et al[34] and Chisholm et al's immunosuppressant therapy questionnaire.[35] As part of the post-visit survey patients will be asked three questions about their intention to follow through and their past history of following through on recommendations made by a healthcare professional. Patients will rate their response to the question: "I intend to follow through on the next steps (care plan) I discussed with my doctor during my appointment." On a 5 point scale from "extremely unlikely" to "extremely likely." They will also be asked, "Over the last three months, how often did you do what your doctors (or other healthcare professionals) recommended?" and "Looking back over your entire life, how often did you do what your doctors (or other healthcare professionals)

recommended?" With answers ranging from "never" to "always." We will examine the psychometric property of these measures and expect that they will enable us to form a factor that measures the construct of intention to adhere to care plans. We call this measure INTENTION. These measures will be collected in the short survey containing CollaboRATE and Facilitation, mention above.

Upon considering the comments from reviewers and PCORI program officers, we have decided to add another survey of patients 3 months after the indexed visit. We will work with our stakeholders and adapt the adherence measure developed and tested in the Medical Outcomes Study (MOS).[36] The MOS general adherence survey instrument has 5 questions:

(a) I had a hard time doing what the doctor suggested I do;
(b) I found it easy to do the things my doctor suggested I do;
(c) I was unable to do what was necessary to follow my doctor's treatment plans;
(d) I followed my doctor's suggestions exactly; and
(e) Generally speaking, how often during the past 4 weeks were you able to do what the doctor told you?

Response options for each item range from *none of the time* (1) to *all of the time* (6). Responses to the 5 items will be averaged and then transformed linearly to a 0-100 distribution.

With these items as a starting point, we will work with our advisory panels to adapt them to 1) provide a more patient-centered assessment (e.g., item 2 might be reworded to: "I found it easy to do the things my doctor and I agreed I would do". In addition, we will develop and test lead in wording that appropriately references the plan developed at the index visit, and will explore the feasibility and acceptability of piping in the patient's description of the plan (as captured on the immediate post visit survey) as a reminder.

From the Medical Outcomes Study, we assume this general adherence outcome to be normally distributed with a mean of 70, and an SD of 11.[36] Assuming 50% of our patients will have chronic conditions, we will have 80% power to detect a difference of 2.6 points in adherence scores between the groups (ICC=0.02, and alpha=0.05).

A3. Clinical indicators: A range of clinical indicators will be explored to examine the linkages between intervention and patient's health, including patient reported general health status (SF12[37]) for all patients, and blood pressure (BP), hemoglobin A1c (A1c), Low Density Lipoprotein (LDL), for relevant patient subgroups. For example, for patients with hypertension, we will compare their BP over time; for patients with diabetes, their A1c over time; for patients with hyperlipidemia, their LDL over time; based on biometric data in the EHR. SF12 will be obtained in the post-visit survey.

A4. Post-visit service use and other system impact measures: to provide healthcare system leaders with information on impact of the interventions on workflow and resource use, we will examine patient-initiated calls, e-messaging, and office visit after the index visit. This is an exploratory outcome using data from the EHR. We have learned since the launch of the study that UCSD and PAMF use the Physician Efficiency Profile data provided by EpicCare to monitor physician efficiency in using EpicCare.

A5. Chart review: Based on the volume of use of these services, we will further sample the top 5% high users of post-intervention patients and review their medical records including the indexed visit and subsequent services that had occurred within four weeks after the indexed visit. In our previous

research on clinical encounters between patients with chronic conditions, e.g., hypertension, we have observed that physicians asked patients to call or email after a medication was changed to let them know how blood pressure home monitoring was going. The chart review will enable us to decipher the reasons for high volume of services after the indexed visit. We anticipate that some of them could be consistent with patient's goals that were made clearer due to more patient centered communication. One trained nurse chart reviewer will be hired for each site to extract detailed information on use of services such as patient-initiated portal e-messaging, telephone calls, subsequent office visits, and the reasons for using these services. A determination will be made as to how they relate to patient's priority for the indexed visit; PCP's documentation of the visit, and the AVS. The following two cases illustrate what may emerge.

Case 1. Patient 1 wanted to discuss sleep trouble the most. Open communication revealed untreated depression and anxiety disorders for which the PCP prescribed an anti-depressant medication. The patient had questions about the medication side effects after he took them for a few days that he raised in several patient portal e-messages. There could also be a referral for sleep specialist and a sleep lab study, followed by more patient-initiated e-messages. This example would suggest that even though the volume of services was high, the patient was served appropriately according to evidence-based practices. All of these services would be documented in the EHR and AVS for follow-up care.

Case 2. Patient 2 came in for an ear infection following a cold and then slipped on ice and fell two weeks after the visit. The high volume of post-visit services were for urgent care, x-rays, MRI, and physical therapy. These orthopedic and physical therapy services were not related to the most important issue for the visit (ear infection).

<u>A6. Other healthcare system outcomes:</u> We will consider examining the scores of Clinic Group Consumer Assessment of Healthcare Providers Services (CG-CAHPS).[38] CAHPS is a federal initiative to support the assessment of consumers' experiences with health care. It is plausible that the interventions may differentially affect consumer experience across the intervention arms, and in comparison to PCPs or clinics that opted out of the study. Both Sutter and Reliant collect CG-CAHPS data that can be used in this study.

8.2 SAFETY AND OTHER ASSESSMENTS

Not Applicable

8.3 UNANTICIPATED PROBLEMS

8.3.1 DEFINITION OF UNANTICIPATED PROBLEMS (UP)

While we do not anticipate problems, given the nature of the interventions, we will record unanticipated problems if they were to occur.

8.3.2 UNANTICIPATED PROBLEM REPORTING

We will report unanticipated problems to the IRB and the data and safety monitoring board.

8.3.3 REPORTING UNANTICIPATED PROBLEMS TO PARTICIPANTS

We will report such problems to participants if the IRB and the data and safety monitoring board advise us to do so.

9 STATISTICAL CONSIDERATIONS

9.1 STATISTICAL HYPOTHESES

For the primary endpoint, the primary comparison is that of OPEN-High Tech vs. ASK, and it will be performed at the α=0.05 level, as a superiority test. The secondary comparisons are of OPEN-High Touch vs. ASK and OPEN-High Tech vs. OPEN-High Touch. The High Tech vs. High Touch comparison will be a non-inferiority comparison with a non-inferiority margin corresponding to a health-system-level odds ratio of 1.30 between arms, performed as a secondary analysis at level α=0.025 one-sided. (The non-inferiority margin of OR=1.30 corresponds to a difference-of-proportions non-inferiority margin of δ=5% when the null hypothesis of non-inferiority is expressed as H_0: $p_1=p_2-\delta$ and p_1=0.77 [77%]). The OPEN-HIGH Touch vs. ASK comparison will be a separate secondary analysis performed as a superiority comparison trying to detect a 5% difference (77% vs 72%), at level α=0.05 two-sided. No overall, 3-arm comparison will be done. The OPEN-High Tech vs. ASK comparison was chosen as the primary comparison because OPEN-High Tech is a potentially more scalable intervention, with lower costs and great potential for implementation in real world practices.

Primary Endpoint Hypothesis
Proportion of patients in each group on CollaboRATE survey giving the maximum score compared between arms using the hierarchical approach to testing.
δ = 0.05
CollaboRATE proportions
1 (non-inferiority): H_0: $P_{high\ tech} = P_{high\ touch} - \delta$
2 (superiority) :H_0: $P_{high\ tech} = P_{ask}$
3 (superiority): H_0: $P_{high\ touch} = P_{ask}$

Similarly, the Facilitate scores will be compared using the proportion of patients giving the maximum score using the hierarchical approach to testing the arms.

Facilitate proportions:
1 (non-inferiority): H_0: $P_{high\ tech} = P_{high\ touch} - \delta$
2 (superiority) :H_0: $P_{high\ tech} = P_{ask}$
3 (superiority): H_0: $P_{high\ touch} = P_{ask}$

Secondary endpoint:

1. Confidence, test whether arm significantly effects confidence scores

2. Intention is not longitudinal it is a factor so studying this would be a simple mixed effects model possibly, looking for input

3. Clinical Indicators (HbA1c, Blood Pressure, LDL)
4. CollaboRATE scores of 1 and 2, similar to the hierarchical testing from primary endpoints
   1 (non-inferiority): H_0: $P_{high\ tech} = P_{high\ touch} - \delta$
   2 (superiority) :H_0: $P_{high\ tech} = P_{ask}$
   3 (superiority): H_0: $P_{high\ touch} = P_{ask}$

9.2 SAMPLE SIZE DETERMINATION

UCSD has about 89 PCPs with an average panel size of 1,700 patients. PAMF has about 500 PCPs. Reliant has approximately 150 PCPs. The average patient panel size is about 2,000 patients per PCP in both Sutter and Reliant systems.

Analyses of the pilot study data revealed that the estimated within-PCP intra-class correlation (ICC) between baseline and follow-up outcomes was negligible (ICC=0.000004 for CollaboRATE=9, ICC=0.00000005 for Facilitate=9). In the absence of within-PCP ICC, the baseline samples do not help reduce variability, making the statistical analysis just as effective as the direct comparison of the follow-up time points. In other words, the baseline samples have no statistical value if ICC is again negligible. This is confirmed in our statistical simulations. However, if in our study it turns out that the within-PCP ICC is positive (but small), the baseline samples will improve power. For this reason, we proposed to use 10 baseline + 40 trial patients.

The power of the primary and of the two additional pairwise comparisons is included in Tables 1, 2, and 3. The 10+40 distribution will provide enough power (.78 to .84) to detect a 5% difference. Primary comparison is done at α=0.05, with 10 baseline+40 follow-up participants per PCP.

The tables below show the power of the study to detect a difference in the proportion of respondents assigning *CollaboRATE* = 9 between the study arms, as follows: Table 1: Primary comparison, High Tech vs. ASK, superiority testing; Table 2: Secondary comparison, High Tech vs. High

Touch, non-inferiority testing; Table 3: Additional secondary comparison, High Touch vs. ASK, superiority testing. In each case, five scenarios were considered: 1) study design as originally proposed (1-way ANOVA with Bonferroni-corrected post-hoc comparisons, 25+25 participants per PCP, difference of differences analysis); 2) comparison done at α=0.05, 25+25 participants per PCP, difference of differences analysis); 3) comparison done at α=0.05, 25+25 participants per PCP, compare follow-up time points only – this is in effect equivalent to a 0+25 participants per PCP; and primary comparison done at α=0.05, 10 baseline+40 follow-up participants per PCP, compared using 4) baseline and follow-up data, and 5) follow-up data only – the latter is in effect equivalent to a 0+40 participants per PCP.

Within each type of comparison, three scenarios for within-site ICC were considered, ICC_{Site}=0 (optimistic), ICC_{Site}=0.001 (realistic) and ICC_{Site}=0.002 (conservative). In all simulations we assume 7 sites per arm, and an average 5 PCP's per site, with a site-to-site coefficient of variation of 0.3 for the number of PCP's. Each PCP recruits 50 patients, for a total of 1,750 patients for each arm.

The within-PCP ICC is assumed ICC_{PCP}=0, as found in the pilot study (Tai-Seale et al., 2016). Due to this independence of patient ratings for the same physician, the "difference of differences" analysis achieves the same power as the follow-up only analyses (scenarios 2 vs. 3, and 4 vs. 5).

The power calculations were done using a custom statistical simulation program in R for all scenarios.

Table 1. Statistical power for the primary comparison of detecting differences of positive patient ratings between High Tech and ASK groups of 10%, 5%, and 3%, under five different scenarios. The scenarios differ in the type of analysis (DoD = difference of differences, adjusting for baseline; Fup = comparison of follow-up ratings only) and distribution of patients at baseline and follow-up (25+25 = 25 baseline + 25 follow-up; 10+40 = 10 baseline + 40 follow-up). Each arm recruits 7 sites, with 5 PCP per site on average (CV=0.3) and 50 patients per PCP. The within-PCP ICC=0. Within-site ICC=0-0.002.

Primary comparison: High Tech vs. ASK	Power to detect **10%** difference (70% vs 80%)			Power to detect **5%** difference (72% vs 77%)			Power to detect **3%** difference (72% vs 75%)		
Study Design ICC_{PCP}=0.000	ICC_{Site} =0.000	ICC_{Site} =0.001	ICC_{Site} =0.002	ICC_{Site} =0.000	ICC_{Site} =0.001	ICC_{Site} =0.002	ICC_{Site} =0.000	ICC_{Site} =0.001	ICC_{Site} =0.002
1. 25+25 DoD α=0.05/3	>0.99	0.99	0.98	0.49	0.45	0.43	0.18	0.17	0.17
2. 25+25 DoD α=0.05	>0.99	>0.99	>0.99	0.66	0.63	0.60	0.32	0.31	0.30
3. 25+25 Fup α=0.05	>0.99	>0.99	>0.99	0.66	0.63	0.60	0.32	0.31	0.30
4. 10+40 DoD α=0.05	1.0	1.0	1.0	0.84	0.81	0.78	0.43	0.40	0.37
5. 10+40 Fup α=0.05	1.0	1.0	1.0	0.84	0.81	0.78	0.43	0.40	0.37

Table 2. Statistical power for the secondary comparison of non-inferiority evaluating the proportion of positive patient ratings between High Tech and High Touch groups of 10%, 5%, and 3%, under five different scenarios. . The non-inferiority margin of OR=1.30 corresponds to a difference-of-proportions non-inferiority margin of δ=5% when the null hypothesis of non-inferiority is expressed as H_0: $p_1=p_2-\delta$ and p_1=0.77 [77%]. The scenarios differ in the type of analysis (DoD = difference of differences, adjusting for baseline; Fup = comparison of follow-up ratings only) and distribution of patients at baseline and follow-up (25+25 = 25 baseline + 25 follow-up; 10+40 = 10 baseline + 40 follow-up). Each

arm recruits 7 sites, with 5 PCP per site on average (CV=0.3) and 50 patients per PCP. The within-PCP ICC=0. Within-site ICC=0-0.002.

Primary comparison: High Tech vs. ASK	Power to detect **10%** non-inferiority margin (alternative 70% vs 80%)			Power to detect **5%** non-inferiority margin (alternative 72% vs 77%)			Power to detect **3%** non-inferiority margin (alternative 72% vs 75%)		
Study Design ICC_{PCP}=0.000	ICC_{Site} =0.000	ICC_{Site} =0.001	ICC_{Site} =0.002	ICC_{Site} =0.000	ICC_{Site} =0.001	ICC_{Site} =0.002	ICC_{Site} =0.000	ICC_{Site} =0.001	ICC_{Site} =0.002
1. 25+25 DoD α=0.025/3	>0.99	0.99	0.98	0.49	0.46	0.44	0.18	0.17	0.17
2. 25+25 DoD α=0.025[(1)]	>0.99	>0.99	>0.99	0.66	0.64	0.61	0.32	0.32	0.31
3. 25+25 Fup α=0.025[(1)]	>0.99	>0.99	>0.99	0.66	0.64	0.61	0.32	0.32	0.31
4. 10+40 DoD α=0.025[(1)]	1.0	1.0	1.0	0.84	0.82	0.79	0.43	0.41	0.38
5. 10+40 Fup α=0.025[(1)]	1.0	1.0	1.0	0.84	0.82	0.79	0.43	0.41	0.38

(1) Note: All tests are done one-sided, with the alternative showing that the High Tech arm is not inferior to the High Touch arm.

Table 3. Statistical power for the additional secondary comparison of detecting differences of positive patient ratings between High Touch and ASK groups of 10%, 5%, and 3%, under five different scenarios. The scenarios differ in the type of analysis (DoD = difference of differences, adjusting for baseline; Fup = comparison of follow-up ratings only) and distribution of patients at baseline and follow-up (25+25 = 25 baseline + 25 follow-up; 10+40 = 10 baseline + 40 follow-up). Each arm recruits 7 sites, with 5 PCP per site on average (CV=0.3) and 50 patients per PCP. The within-PCP ICC=0. Within-site ICC=0-0.002.

Primary comparison: High Tech vs. ASK	Power to detect **10%** difference (70% vs 80%)			Power to detect **5%** difference (72% vs 77%)			Power to detect **3%** difference (72% vs 75%)		
Study Design ICC_{PCP}=0.000	ICC_{Site} =0.000	ICC_{Site} =0.001	ICC_{Site} =0.002	ICC_{Site} =0.000	ICC_{Site} =0.001	ICC_{Site} =0.002	ICC_{Site} =0.000	ICC_{Site} =0.001	ICC_{Site} =0.002
1. 25+25 DoD α=0.05/3	>0.99	0.99	0.98	0.49	0.45	0.43	0.18	0.17	0.17
2. 25+25 DoD α=0.05	>0.99	>0.99	>0.99	0.66	0.63	0.60	0.32	0.31	0.30
3. 25+25 Fup α=0.05	>0.99	>0.99	>0.99	0.66	0.63	0.60	0.32	0.31	0.30
4. 10+40 DoD α=0.05	1.0	1.0	1.0	0.84	0.81	0.78	0.43	0.40	0.37
5. 10+40 Fup α=0.05	1.0	1.0	1.0	0.84	0.81	0.78	0.43	0.40	0.37

Regarding ICC: the ICCs from our pilot study for the model clustering within providers was 0.007 and for the model clustering for clustering within clinics it was 7.21e-08. Because this is a different cohort, and we are including some different outcome measures, we decided to take a conservative approach and used an ICC of 0.02 for our power calculations. The consequence of overestimating the ICC is that we will have greater power.

9.3 POPULATIONS FOR ANALYSES

Intent to treat analysis will be employed such that all data will be analyzed based on the intervention arm in which patients are located without regard to whether the intervention was fully carried out on them or not. As with the pilot study, we will use two approaches to examine the main outcomes, patient reported experience with care, top score (for primary analysis) and actual score (for sensitivity analysis described below in section C5.5), to ensure robustness of evidence. The top score is whether or not the patient gave the top score (i.e., 9) on all three questions of CollaboRATE, or 9s for all five questions of Facilitation. The top score method gives more variation than mean score and accounts for some patients viewing these kinds of surveys as dichotomous, i.e. 9 is good, anything less is not good.[15,40] The dependent variable will be 1 if the patient gave a score of 9 to all items of CollaboRATE or Facilitation and 0 otherwise. We will use multiple imputation methods to address missing data. (More on missing data below.)

Fidelity - We will measure patient and PCP use of our interventions to the extent they can be captured in the EHR, to inform the exposure level of interventions. They include patient responses to previsit patient survey that serves as a nudge about informing providers about what is most important for the visit to address, access to the animated video, PCP visits with in-person SPI, PCP access to virtual SPI in the two OPEN arms. We will extract data on access to AVS in the EHR made by all patients of participating PCPs in each arm. This information can offer contextual information that can help us interpret the intent to treat analysis results.

We have published a paper in Health Affairs on our innovative approach to use EPIC's user access log to study clinicians' work effort.[41] This approach will enable us to measure fidelity.

At the same time, we will send out "stay in touch" monthly emails, share study results, and leverage the reach of the stakeholders among health system leaders and clinicians to keep clinicians involved. We will also ask about fidelity in the post-intervention provider and medical assistant survey. We understand that Maintenance of Certification (MOC) is an important incentive to physicians. We will work with ABIM and AAFP to enhance the importance of the project to clinicians.

9.4 STATISTICAL ANALYSES

9.4.1 GENERAL APPROACH

Data Preparation Prior to analysis, the data files will be checked for out of range, otherwise invalid values, and outliers. Following standard research practice, we will perform preliminary analyses required for variable construction, checking for the validity of missing at random assumption and perform multiple imputations of missing data, as needed. We will also assess psychometric properties of study variables (e.g., calculate Cronbach's alpha) before analyses of intervention effects. Data set documentation will then be developed and distributed to the study team. This documentation will include descriptions of variables, algorithms for calculated variables and variable distributions and relevant references. Non-response rates will be calculated and monitored at each stage of data collection.

To ensure that the randomization will achieve the goal of having balanced clinics and PCPs within them, we will analyze administrative data on clinics and PCPs at the baseline, before randomization. The data will include the number of PCPs per clinic, and PCP age, sex, and race/ethnicity. Participating PCPs' patient panel characteristics at the baseline will also be compared to account for their social demographics, and their experience with care at the baseline. Before carrying out analyses addressing a particular research question, an analysis plan will be formally presented to the study team, critiqued and revised as needed. Data management and analyses will be carried out in SAS and R.

Data-Linkage Plans (IR-2) – UCSD, PAMF, and Reliant Health all use EpicCare EHR. We have had many years of experience using EHR data and survey data for research. Patient and PCP surveys will be administered in RedCap, a secure web-based survey software housed behind the firewalls. A study ID will be assigned to each participant with which to link data from various sources. We will establish a Data Use Agreement to facilitate HIPAA compliant transfer of de-identified and cleaned data from PAMF and Reliant to UCSD after each wave of surveys has been completed.

Mixed effects logistic regression will be used to analyze the data following descriptive analyses. For the main patient reported outcomes, we will examine the association between the patient's assignment in an intervention group and giving the highest possible CollaboRATE or Facilitation score. The dependent variable is 1 if the patient gave a score of 9 to all 3 items of CollaboRATE (or all 5 items of Facilitation) and a 0 otherwise. Because multiple patients of each PCP will be participating (i,e., 40 patients/PCP), the models will account for clustering of patients within PCP by using cluster robust standard errors. We will also account for clustering at the clinic level as we plan to recruit 5 PCPs per clinic. The models will also control for patient level demographic variables including age, sex, race/ethnicity, and education. Furthermore, we will control for PCP variables such as age, sex, race/ethnicity, prior communication training and length of relationship with the patient. The approach will generally be similar for other outcomes with appropriate adjustments made after we examine the descriptive analyses results.

9.4.2 ANALYSIS OF THE PRIMARY ENDPOINT(S)

The pilot study showed a difference of 8% on our primary endpoint (CollaboRATE=9) between High Touch and Usual care (74.7% vs 66.7%) but of approximately 3% between High Touch and ASK (74.7% vs 72.0%), see Table 2 in Tai-Seale et al. (2016). Upon review, the 10% difference in the original proposal between High Tech and ASK, and between High Touch and ASK may be optimistic, even though not impossible, given that the confidence interval of findings from the pilot study included 10% and the potentially greater diversity in clinical practice patterns across 3 health systems. A 5% anticipated difference may be moderately realistic, while a 3% anticipated difference is conservative. Our calculations show that with the proposed changes in study design we have enough power to detect a difference of 5% between High Tech and ASK.

As a further justification for the 5% point difference, our observations of physician performance evaluations associated with patient satisfaction scores in real world practices suggest that sometimes even a 1% difference could determine whether a physician would receive a performance reward or not.

If a patient satisfaction metric is set at 80%, someone with a score of 79% will not receive a bonus pay, whereas someone with a 84% score would. The bonus pay could be 5% of one's salary and that is a non-trivial amount.

Tai-Seale has contributed to the research literature that suggests that patients' choice of physicians is influenced by report cards on patients' experience with physician practices. We have also heard from some of the health system stakeholders on our study that they are eager to know even a 1% difference in a particular patient reported experience measure, i.e., the intent to recommend the physician they saw to family and friends. Our study will include this intent to recommend measure and other patient reported experience measures. We are confident that our study will produce salient information that will be meaningful to patients, physicians, and health care systems in decision making.

To analyze our primary endpoint, we will use a logistic mixed effects approach to data analysis, comparing the relevant outcome variables between the arms using the fixed effects (X) and random effects (Z) on the relevant outcomes (Y) with normal error (ε) using the following model:

$$\text{logit}(Y|\gamma) = X\beta + Z\gamma + \varepsilon$$

We will obtain information on covariates (controls) in our model from both administrative and survey data. From EHR data, we can control for patient age, sex and the presence of health conditions and clinical indicators e.g., BP, A1c, and LDL. We will also control for the baseline rating of the doctor. We also have information on patient access of pre-visit survey of what matters most to them, their viewing of the animated video, and whether they access the After Visit Summary (AVS) from the "digital trail" in the access log data in EHR.[28]

<u>Addressing Missing Data</u>: Missing data (MD) could come in three forms: missing patient reported outcomes, missing PCP reported outcomes, or missing EHR data. Missing data on these outcomes will be due to one of three situations: (1) non-response to patient survey; (2) non-response to PCP survey; and (3) returned incomplete questionnaire, in the event that some respondents leave some items in surveys unanswered.

We plan to analyze the non-responders according to EHR and administrative data on patients and PCPs, where available, carefully considering any related changes in interpretability of our estimated effects. To assure maximal participation, we will collect survey data via the web, with financial incentives for completing the surveys. With our survey tracking system, we will identify subjects who are "out of window" for their return of their survey and all respondents who only partially complete a returned survey. In either case, we will attempt to contact them to obtain the information via the patient portal again. After three attempts, we will treat their survey as incomplete or missing, record it as such, and a

judgment will be made as to whether the completed questions provide enough information to impute an overall score. (MD-1) If so, we will employ methods of multiple imputation to fill in the missing data for partial surveys. (MD-2, 3)

We do not anticipate that missing survey data will be informative with respect to the treatments (Open High Touch, Open High Tech, ASK), and will have comparative EHR clinical or administrative data for them to determine whether selection bias is relevant to the evaluation of the change in patient reported outcomes. If necessary, we will profile the respondent subgroup and restrict interpretation of patient-reported outcome results to this population. (MD-5) We will prepare an annual report of all missing data including an assessment of bias. (MD-4)

Regarding the possibility of missing EHR-sourced data, while we do not anticipate a significant issue of missingness in outcome measurements, some missing data in baseline and demographic characteristics is likely. We will conduct a by-variable evaluation of missing data in this setting and determine in each case how missing data should be treated in our analyses. (MD-5)

9.4.3 ANALYSIS OF THE SECONDARY ENDPOINT(S)

Patient Survey 3 Months After Indexed Visit

The survey of patients 3 months after the indexed visit is to measure change over time of secondary outcomes, including clinical and utilization outcomes, and adherence to treatment plans. Repeated measures will enable us to determine whether there is a lasting effect of the intervention beyond the initial improvement in communication. In addition, it will allow us to measure whether there are meaningful changes in utilization and clinical outcomes after intervention.

To measure utilization, we will focus on Diabetes. We estimate that 6.3% of our primary care patients have diabetes, and 75% of these patients will not have had their hemoglobin A1c tested within 6 months. If we can reduce this percentage to 54%, we will have sufficient power to detect the change (power=0.8, alpha=0.05, one-sided test). For this estimate, two-level clustering (at the provider and clinic level) with ICCs of 0.02 was assumed, and approximate sample sizes within each arm were used. Because we expect less than 25% of people with diabetes to have A1c measurements, we will not have sufficient power to detect changes in A1c between time periods. However, we will record these and look for trends.

Our main clinical outcome is controlled hypertension. From preliminary data from a random sample of primary care patients, we estimate that approximately 25% of our patients will have hypertension, and of those, 30% will be uncontrolled. Using a one-sided test, we expect to be able to detect an 11 percentage-point decrease (30% to 19%) from visit 1 to 3-month follow-up in the percentage of patients with uncontrolled hypertension (power=0.8, alpha=0.05).

We also plan to measure quality of life using the VR-12 with a standardized scale. Assuming a mean of 50 and an SD of 10, we will have 80% power to detect a difference of 1.7 units, and alpha=0.05, ICC=0.02.

Confidence

The patients' confidence in their ability to follow the treatment plan decided upon in collaboration with their doctor is measured both at the indexed visit in the immediate post-visit survey as well as in the 3 month follow-up survey. This allows for the analysis of this confidence using a longitudinal analysis with only two time points. We will use a general linear model for longitudinal data:

$$Y = X \beta + \varepsilon$$

This will allow us to determine whether the Arm a patient is in informs the trajectory of confidence scores over those two time points. The specific test will be whether the coefficient for time point by arm is significant in the model. In this model we will also control for site and doctor to explain some of the variation in response.

Intention

Intention is a factor created from the scores of 3 likert scale questions. We will use the average score given on intention to follow through with the plan set forth by a patient and PCP. This average will be used as an outcome in a linear mixed effects model, controlling for both PCP, clinic and site.

Clinical Indicators

Another secondary endpoint to consider in our analysis will be those clinical indicators collected from EHR at point of visit (T1), 3 months post visit (T2) and 12-month post visit (T3). We will compare these time points pairwise using mixed effects longitudinal models. These models will have random effects for system, clinic and PCP.

For each clinical indicator we will compare T2 between the arms controlling for the baseline value (T1). Similarly, compare T3 between the arms controlling for the baseline value (T1).

For the purpose of analysis, we will transform some of the clinical indicator variables. Blood pressure does not need to be transformed. As HbA1c is right skewed it will be $\log_e$ transformed for statistical analysis. We will also log transform LDL cholesterol. VR12 has a normalized scoring rubric which will be used and therefore no transformation will be necessary. We will perform this model predicting for the ratio between LDL and total cholesterol as that outcome is of more importance clinically.

These models will only control for age, since there are so many participants there is no need to control for other confounders.

ColloboRATE Low Scores:

Similarly to primary endpoint analysis, we will use a logistic mixed effects approach to data analysis, comparing the proportion of patient that gave a score of 1-2 on all collaborate questions between the

arms using the fixed effects (X) and random effects (Z) on the relevant outcomes (Y) with normal error (ε) using the following model:

$$\text{logit}(Y|\gamma) = X\beta + Z\gamma + \varepsilon$$

We will obtain information on covariates (controls) in our model from both administrative and survey data. From EHR data, we can control for patient age, sex and the presence of health conditions and clinical indicators e.g., BP, A1c, and LDL. We will also control for the baseline rating of the doctor. We also have information on patient access of pre-visit survey of what matters most to them, their viewing of the animated video, and whether they access the After Visit Summary (AVS) from the "digital trail" in the access log data in EHR.[28] For continuity, we will use an odds ratio margin of 1.3.

9.4.4 BASELINE DESCRIPTIVE STATISTICS

We will begin by obtaining descriptive statistics on relevant outcome measures across patients, PCPs, and clinics according to their randomized assignments in the RCT. We will examine and report the absolute differences between PCPs and within PCPs over time (i.e., baseline vs. post-intervention) in patient reported experience with care and additional outcome measures.

9.4.5 SUB-GROUP ANALYSES

N/A

9.4.6 TABULATION OF INDIVIDUAL PARTICIPANT DATA

We do not have any such tabulations

9.4.7 EXPLORATORY ANALYSES

Planned Sensitivity Analyses (*IR-5*) As a sensitivity analysis, a structural equations method (SEM) employing the actual scores given by patients will also be applied. We will also use an alternative specification which treats CollaboRATE and Facilitation as latent variables which are predicted by observed variables reflected by the actual score given by patients. A special case of SEM, known as a Multiple Indicators Multiple Causes (MIMIC) model,[42] will be used to examine the effect of the intervention on latent variables CollaboRATE or Facilitation. Prior to estimating the MIMIC model, we will conduct a confirmatory factor analysis to validate the structure of CollaboRATE and Doctor Facilitation. In the pilot study, the one-factor CFA had 3 items loading onto the single factor CollaboRATE, with the Cronbach's alpha=0.96. The one-factor CFA had 5 items loading onto the single factor Facilitation, with the Cronbach's alpha=0.85. This model and the subsequent MIMIC model will account for clustering of patients within physicians and physicians within clinics by permitting the errors

to be correlated within clusters. This and the subsequent MIMIC model will be estimated using full information maximum likelihood (FIML) estimation which allows all available data to be used.

Following the CFA, we will estimate the MIMIC model which consists of the measurement model discussed in the previous paragraph and a structural part, where the latent variable CollaboRATE or Facilitation will be predicted by observed variables on intervention groups and patient demographics. The models will control for patient level demographic variables including age, sex, race/ethnicity, and education, as well PCP variables such as age, sex, race/ethnicity, and length of relationship with the patient.

Use of Resources Patients who experience worse communication in their doctor's visit can use resources such as email, phone and additional office visits to get information sorted out for how they are supposed to proceed with their care plan. We will analyze the use of resources from patients in the study after the indexed visit. We have a count of how many times a patient emails or calls the doctor's office for 12 months after the initial visit. We will use a Poisson model to see whether the arm of treatment affects how much a patient uses the resources at their disposal. We will control for whether the patient had an office visit for another reason that is not related to the our study in the time between initial visit and 12 months.

10 SUPPORTING DOCUMENTATION AND OPERATIONAL CONSIDERATIONS

10.1 REGULATORY, ETHICAL, AND STUDY OVERSIGHT CONSIDERATIONS

10.1.1 INFORMED CONSENT PROCESS

10.1.1.1 CONSENT/ASSENT AND OTHER INFORMATIONAL DOCUMENTS PROVIDED TO PARTICIPANTS

Patients in the OPEN arms will receive a notification via their patient portal, MyChart, 3-21 days prior to their visit. The notification will contain a brief description of the study (see attached draft) and a link to an online informed consent and HIPAA authorization document in REDCap on UCSD's secure server. After consenting, the patient will receive a one question survey through MyChart asking them to think about what they want to discuss at their upcoming scheduled appointment along with a link to a brief animated online video which is aimed at helping to prepare them for their appointment with the PCP. Within one week following their completed appointment, patients will receive an email notification from the research team through their study email (opencommunication@ucsd.org) and will be directed to an online survey platform (REDCap) hosted by UCSD to answer questions about their recent appointment.

Patients in the ASK arms will receive a notification via MyChart, 3-21 days prior to their visit. The MyChart notification will contain a brief description of the study and a link to an online informed consent and HIPAA authorization document in REDCap hosted by UCSD. Within two weeks following their completed appointment the patients will receive an email notification to the email they provided in the consent process and will be directed to an online survey platform (REDCap) hosted by UCSD to answer questions about their recent appointment.

We will provide the patient service representatives (PSRs) and volunteer greeters at each clinic some instructions on how the study (see attached) for giving out an information sheet for patients (see attached) providing some basic information about the study and study team contact information (phone number and email address). That way, the PSRs will not have to spend time explaining the study but can direct patients to some more information. These patient information sheets will also be given to the Mas/LVNs and physicians so that they can give to participants with questions and not take away time from the visit.

All enrolled patients will be asked to complete a follow-up survey 3 months after the indexed visit. This follow-up survey will enable us to measure adherence to PCP recommendations among all enrolled patients. These surveys will also be sent out by REDCap.

10.1.1.2 CONSENT PROCEDURES AND DOCUMENTATION

Informed consent is a process that is initiated prior to the individual's agreeing to participate in the study and continues throughout the individual's study participation. Consent forms will be Institutional Review Board (IRB)-approved and the participant will be asked to read and review the document. The investigator will explain the research study to the participant and answer any questions that may arise. A verbal explanation will be provided in terms suited to the participant's comprehension of the purposes, procedures, and potential risks of the study and their rights as research participants. The participant will sign the informed consent document prior to any procedures being done specifically for the study. Participants must be informed that participation is voluntary and that they may withdraw from the study at any time, without prejudice. A copy of the informed consent document will be given to the participants for their records. The informed consent process will be conducted and documented in the source document (including the date), and the form signed, before the participant undergoes any study-specific procedures. The rights and welfare of the participants will be protected by emphasizing to them that the quality of their medical care will not be adversely affected if they decline to participate in this study.

10.1.2 STUDY DISCONTINUATION AND CLOSURE

Not Applicable

10.1.3 CONFIDENTIALITY AND PRIVACY

Participant confidentiality and privacy is strictly held in trust by the participating investigators, their staff, and the sponsor(s) and their interventions. This confidentiality is extended to all electronic health record information extracted. Therefore, the study protocol, documentation, data, and all other information generated will be held in strict confidence. No information concerning the study or the data will be released to any unauthorized third party without prior written approval of the sponsor.

All research activities will be conducted in as private a setting as possible.

Federal government regulatory agencies and the UC San Diego Health Institutional Review Board (a committee that reviews and approves research studies) may inspect and copy records pertaining to this research.

The study participant's contact information will be securely stored at each clinical site for internal use during the study. At the end of the study, all records will continue to be kept in a secure location for as long a period as dictated by the reviewing IRB, Institutional policies, or sponsor requirements.

Study participant research data, which is for purposes of statistical analysis and scientific reporting, will be transmitted to and stored at the UCSD ACTRI Virtual Data Center. This will not include the participant's contact or identifying information. Rather, individual participants and their research data will be identified by a unique study identification number. The study data entry and study management systems used by clinical sites and by Shuxiang Liu at UCSD will be secured and password protected. At the end of the study, all study databases will be de-identified and archived at the UCSD ACTRI Virtual Data Center.

Certificate of Confidentiality (if applicable) – Not applicable.

10.1.4 FUTURE USE OF STORED DATA

Data collected for this study will be analyzed and stored at UCSD ACTRI Virtual Data Center. After the study is completed, the de-identified, archived data will be transmitted to and stored at the UCSD Medical Library, for use by other researchers including those outside of the study. Permission to transmit data to the UCSD ACTRI Virtual Data Center and the UCSD Medical Library will be included in the informed consent.

When the study is completed, access to study will be provided through the University of California San Diego Medical Library.

Any information that could identify participants will be kept on a secure computer that is stored inside the UC San Diego Health firewall. We will keep participation in this research study confidential to the extent permitted by law. Federal government regulatory agencies and the UC San Diego Health Institutional Review Board (a committee that reviews and approves research studies) may inspect and copy records pertaining to this research. Some of these records could contain information that personally identifies participants. If we write a report or article about this study, we will describe the study results in a deidentified way. However, it is possible that other people may become aware of participants in this study.
A description of this clinical trial will be available on http://www.clinicaltrials.gov, as required

by U.S. Law. This web site will not include information that can identify participants. At most, the Web site will include a summary of the results.

10.1.5 KEY ROLES AND STUDY GOVERNANCE

Provide the name and contact information of the Principal Investigator and the Medical Monitor.

**Principal Investigator**
Ming Tai-Seale, MPH PhD, Professor
University of California San Diego
9500 Gilman Dr #0725<br>*La Jolla, CA 92093*
858-246-1939
mtaiseale@ucsd.edu

The Executive Committee consists of the PI, the two Site-PIs, and a co-investigator on the research team who is the Director of the Palo Alto Medical Foundation Research Institute. The Steering Committee consists of stakeholders from each study site that represent health care system leaders, patients, and support staff. The Manual of Procedure (MOP) entails the following roles and responsibilities of those involved in the conduct, management, or oversight of the trial.

E1. Outstanding Research Team

We have assembled an outstanding research team comprised of seasoned researchers and research partners from three operating healthcare systems: UCSD Health, Palo Alto Medical Foundation, and Reliant Medical Group. Our researchers are based at UCSD, Palo Alto Medical Foundation Research Institute, University of Massachusetts, Meyers Primary Care Institute and Reliant Medical Group. We will be working with several consultants lending expertise in targeted areas.

E1.a. UCSD: Principal Investigator, **Ming Tai-Seale**, **PhD, MPH**, is Professor at UCSD School of Medicine, Department of Family Medicine and Public Health, and Director of Outcomes Analysis in the UCSD Health Information Service Department. She was Associate Director and Senior Scientist at PAMF Research Institute (PAMFRI). She is a Consulting Investigator at PAMFRI. She has been leading externally funded patient centered healthcare system improvement research at PAMFRI for the past eight years. She has collaborated extensively with PAMF and Sutter Health clinical leaders and with leading researchers in the HCSRN (e.g., Dr. Kathy Mazor). As PI, she led the pilot study from post-award pre-launch to its successful conclusion. She has assembled the outstanding research team that designed this study. Since relocating to San Diego in early September, she has also been able to establish collaborative relationships with her new colleagues at UC San Diego, from frontline primary care physicians, to health information services leaders, and clinical operations leaders. **Christopher Longhurst, MD**, **MS,** co-investigator, is a pediatrician and the Chief Information Officer at UC San Diego Health System, where he has been leading the organization's health information technology strategy since 2015. He has been nationally recognized for developing innovative EMR tools, including physician decision-support tools

and family-centered portal adaptations, to improve care for children. He is in a unique position to provide specific project guidance along with a knowledgeable view of where specific projects fit in the larger health information technology landscape. He has been unequivocal in his support of the study. He will serve on the guidance team for the study, attend quarterly stakeholder meetings, and participate in writing papers. He leads the weekly GEMBA walks (a rounding for operational leaders in the IS department which is also attended by Dr. Ming Tai-Seale). He and Dr. Tai-Seale often meets after the GEMBA walk to touch base and problem solve on study related issues. **Marlen Millen, MD**, co-investigator, is the Chief Medical Information Officer for Ambulatory Care at UCSD Health System, and the Director of Clinical Service for the Division of Internal Medicine at UCSD School of Medicine. She will oversee and manage operations on UCSD's patient portal, MyUCSDChart, to push reminders to patients regarding their upcoming appointments, invite them to inform their providers on the most important matter, and administer the post-visit surveys. She will also work with the research team to determine how best to integrate the interventions with the EHR system. She will ensure a smooth integration of the study workflow with the clinic workflow to maximize participation by her physician colleagues and their patients. She meets weekly with Dr. Tai-Seale to address emergent and ongoing study-related issues. **Gene Kallenberg, MD**, co-investigator, Professor and Chair of UC San Diego Family Medicine Division. He has already been instrumental introducing Dr. Tai-Seale and the study to faculty members in the Family Medicine clinics. He has already secured commitment from six physicians in Family Medicine to participate in the study. He will serve on the guidance team, attend stakeholder meetings, invite his colleagues to participate in the study at faculty meetings, and encourage his own patients to participate. As a member of the faculty in the department of Family Medicine and Public Health, Dr. Tai-Seale attends the monthly faculty meeting at the Family Medicine division chaired by Dr. Kallenberg. **Florin Vaida, PhD**, co-investigator, is a Professor of Biostatistics at UC San Diego School of Medicine, Department of Family Medicine and Public Health, Division of Biostatics. Dr. Vaida will work closely with Dr. Tai-Seale in developing the study protocol, analytical plan, supervise analyses, and co-author papers. **Bernice Ruo, MD, MAS**, co-investigator, an internist and health services researcher in the Internal Medicine department at UCSD. She will be the champion of the study in the Internal Medicine Department, in addition to serving on the research team, and the Open Communication High Tech work group in particular. **Shuxiang Liu,** a seasoned program analyst at the UCSD Family Medicine division, will serve as the program analyst for the research project. HIPAA-certified, she will be responsible for extracting EHR data, operational data, and constructing data tables according to specifications defined by Dr. Vaida. A to be names senior member of faculty from the UCSD School of Medicine's **Standardized Patient Program** will serve as a member of the research team, strengthen the SPI component of the project, and train the SPIs. Dr. Tai-Seale has been in consultation with the **Center on Health Design in the UCSD Design Lab**. She will be able to recruit a faculty member to the study from among several that have expressed interests. The focus of that center is on patient-centered design of healthcare systems, making its faculty ideally suited to contribute to this project.

Patient Stakeholders: **Sally Cho**, a long-time patient at UCSD Health, has joined the team as a patient stakeholder. Two more patients are being sought from among UCSD patients.

Consultants: We are happy to also be working with some great consultants from across the country to provide their expertise with patient advocacy, SPI training, and patient-reported outcomes measurement. **Libby Hoy** is the founder and CEO of Patient and Family Centered Care (PFCC) Partners and will be a consultant on the project. She will provide guidance throughout the project on how best to improve the quality and experience of healthcare for patients. She will be particularly instrumental in the dissemination phase by spreading the study results throughout PFCC and to other advocacy organizations she works with like Family Voices CA and Partnership for Patients, Patient & Family Engagement Network. **Alison Venuti** is a Physician Communication consultant and was one of the SPI trainers for the initial pilot project. She will be the lead SPI trainer and will assist in training all the SPIs, with her primary focus being on the SPIs from Reliant. **Glyn Elwyn, MD, MSc, FRCGP, PhD** is a Professor and Senior Scientist at the Dartmouth Institute for Health Policy and Clinical Practice, Dartmouth College, and is world renowned for his work in shared decision making. He consulted on the initial pilot project and will continue to provide his feedback and expertise on utilizing CollaboRATE. **Gerald Arnold**, a senior statistician for the American Board of Internal Medicine (ABIM), will be a stakeholder for the project. He will collaborate with **Dr. Rebecca Lipner**, Senior Vice-President of Assessment and Research of ABIM, to provide the perspective how PCPs could benefit from feedback on how to improve communication. They will inform the study team regarding ABIM's evolving Maintenance of Certification requirements so that the interventions developed in this study can facilitate physicians attaining Maintenance of Certification.

E1.b. PAMFRI: **Cheryl Stults, PhD,** site-PI, is a medical sociologist who focuses on qualitative research. She co-managed the pilot study and will have primary responsibility of project management and coordination of research and stakeholders. She will also supervise research staff and stakeholder engagement and participate in analyzing the qualitative survey responses. **Dominick Frosch, PhD**, PAMF's Chief Care Delivery Evaluation Officer (CCDEO), led the initial study[3] that provided the basis for the PCORI pilot grant. He also led the effort to apply for and was awarded funding for the pilot. Dr. Frosch subsequently went to the Gordon and Betty Moore Foundation and at that time transitioned the project to Dr. Tai-Seale's leadership. Dr. Frosch was an active participant in the project at every step of the way and recently returned to PAMF to take on this newly created role as CCDEO. In this capacity Dr. Frosch is responsible for helping the PAMF executive leadership identify and subsequently spread interventions to improve patient care, leaving him exceptionally well-positioned to support this project. **Albert S. Chan, MD, MS, FAAFP**, Vice President, Chief of Digital Patient Experience at SutterHealth, will be a co-investigator. He is a nationally known expert in health IT and 2014 winner of EpicCare's PACAcademy Award. He has over 10 years of experience in the implementation of the electronic health record (EHR) and is part of the team that launched PAMFOnline (now called MyHealthOnline), a personal health record (PHR) co-developed with Epic Systems Corporation. He and a few members of his staff will develop the capacities in MyHealthOnline to send messages and surveys to patients. In addition, his institutional knowledge and leadership capacity will support the implementation of the project. **Ed Yu, M.D**, Medical Director of Quality at PAMF is a co-investigator and will bring his knowledge of quality improvement and patient safety activities for the PAMF and the best ways to

integrate them with minimal disruption on workflow. He will also help with recruiting 9 primary care clinics and PCPs within those clinics to participate. **Ellen Uhrbrock**, a longtime PAMF patient and a patient stakeholder for the Open Communication pilot project, will be a co-investigator for the study. She will bring her vast experiences as a patient with both PAMF and the pilot project to ensure that the best interests of the patient study participants are being met. **Tony Chen**, Data Analyst, has extensive expertise in secure access to PAMF data and will ensure that all regulations and policies protecting patient privacy are followed. His expert analyses of PAMF EHR data have enabled us to carry out multiple PCORI-funded studies.

Stakeholders: The stakeholder members of the research team will be involved in all aspects of research design and interpretation. They bring diverse and important perspectives to the study. We have four patient stakeholders. **Chet Frankenfield** has been a PAMF patient for over 25 years and was a patient stakeholder in the Open Communication pilot project. **Beth Gribbin** is a PAMF Patient Advisory Council Advisor for the past several years and has extensive experience interacting with clinics and departments all over PAMF. She has shadowed PCPs and visits with patients and as such, will be able to provide not only her own perspective, but also that of the many PAMF patients she has come in contact with. **Patricia Alexander** is our third patient stakeholder and is a PAMF patient and a caregiver to her aging mother. She will be able to provide a much needed perspective on how we can better meet the needs of caregivers. Our two physician stakeholders, **Peter Cheng, MD** and **Dominique Quincy, MD** were part of the pilot project so they will continue to share their insights. Dr. Quincy has a new position as leader of the primary care service line and will be invaluable for knowing how to best approach PCPs to participate in the study. We have three clinic staff stakeholders who will be able to comment on how various aspects of the interventions will affect clinic workflow and ways to minimize that impact. **Kelly Reilly, RN** and **Andrea Aguirre, CMA** were also stakeholders for the pilot project and so they will be able to utilize those experiences to help design and implement the interventions. **Jimmy Hu, MD,** President of PAMF Alameda Division, and **Alan Aman**, Chief Operating Officer, are leaders of a current organizational effort entitled PAMFCARES. The goals of PAMFCARES are similar to the proposed project to improve patient experiences and so these two clinic organizational stakeholders will be able to help fully align the study with what has already occurred within PAMF. **Roberta Mori, RN MSN** is the new Director of Patient Engagement for Sutter Health and will be a SutterHealth leadership stakeholder. She will provide her institutional knowledge about the best ways to approach clinics and physicians to participate.

<u>E1.c. Meyers and Reliant</u>: Dr. Kathleen Mazor is Professor of Medicine at the University of Massachusetts Medical School (UMMS), and Associate Director of the Meyers Primary Care Institute. Dr. Mazor is a psychometrician whose research focuses on improving physician-patient communication, with an emphasis on improving patient understanding. She led a multi-site NCI-funded project on health literacy, and has been a leader in emphasizing the importance of spoken information in health literacy[43-47]. Dr. Mazor's current research is focused on understanding the patient's perspective on health and healthcare. She leads an AHRQ-funded demonstration project titled "Detecting, Addressing and Learning from Patient-Perceived Breakdowns in Care" which is testing an intervention designed to encourage

patients to speak up when they have concerns about their care[48]. **Dr. Sarah Cutrona,** Assistant Professor of Medicine and a general internist, brings expertise in studying patient, provider, system and social factors and their influences on primary care, and a practicing physician's perspective. Dr. Cutrona has expertise in patient portal-based interventions, and is leading a study using portal-based messaging to patients to encourage vaccination (with Drs. Mazor and Garber as collaborators[49]). For that project, the team successfully engaged several thousand Reliant patients via Epic's patient portal-based messages (analyses underway). **Dr. Robert Yood** is Medical Director of Reliant Medical Group's Research Department, Director of Reliant Medical Group's Division of Rheumatology, and Clinical Professor of Medicine at the University of Massachusetts Medical School. As a senior clinician investigator, Dr. Yood's research activities have focused on the use of administrative databases and electronic health records to evaluate the incidence and treatment of rheumatic diseases. Dr. Yood has collaborated extensively with both Drs. Mazor and Garber on multiple prior studies. **Dr. Larry Garber** is a board certified internist and clinical informaticist, as well as a Health and Human Services (HHS) Office of the National Coordinator (ONC) Health Information Technology (HIT) Fellow and a member of ONC Policy Committee's Interoperability Workgroup. He has extensive experience and expertise in designing and implementing information technology systems. He led the HIMSS Davies Award-winning implementation of Reliant Medical Group's HIMSS Stage 7 Epic Electronic Health Record (EHR) system.

Stakeholders: The Massachusetts site has a panel of 8 stakeholders: 3 patients, 2 physicians, 2 clinic staff, and 1 for patient experience. **Bonnie Cassidy**, Vice President, Sales and Account Management, Health Data Innovations, is a patient stakeholder and has been a patient of Reliant Medical Group (RMG) from approximately two years. She has almost 20 years of professional experience with health data informatics and is enthusiastic about the opportunity to be part of a study that utilizes health data to improve communication with patients. **Paul Mahon, PhD**, is a patient stakeholder who has been a patient at RMG for over 40 years. He also was a member of the St. Vincent IRB for 38 years; he brings extensive knowledge, and past research experience to the team. **Patrick George** is a patient stakeholder and has been a patient of RMG for over 35 years. He is the owner of a local business, on the Board of Directors of several committees in his town, and is a strong communicator. **Robert Jandl, MD** is the Regional Executive Medical Director and a Physician Champion for Patient Experience at RMG and is a physician stakeholder. Dr. Jandl has been a practicing physician for over 25 years. At Reliant, he directed and coordinated a newly developed organization-wide provider coaching program aimed at improving provider-patient communication skills. He also co-developed teaching programs for staff and providers on patient experience as well as patient experience training programs for managers and supervisors. **Thad Schilling, MD** is Executive Regional Medical Director for RMG and a physician stakeholder. Dr. Schilling has been a practicing physician for 15 years. He is currently Director of 5 adult primary care medical practices that comprised of about 30 physicians and 10 advanced practice clinicians who care for approximately 55,000 patients at Reliant Medical Group. He will be instrumental in the recruitment of clinicians for the study. **Nicholas Kelliher,** Medical Assistant Supervisor at RMG, is a clinical staff stakeholder and has worked at RMG for over 5 years. He is involved with direct patient care, as well as training and supervision of other staff members. He is also currently Chairperson for the Medical Assistant Leadership Council. **Ned Faltas**, Practice Manager of Westboro Internal Medicine, Family Practice and Pediatrics, is a clinical stakeholder and has worked at RMG for over 6 years. He is

responsible for mentoring and coaching department personnel in improvement activities; he has also co-developed and delivered various Lean/continuous improvement training classes at Reliant and so he will be instrumental in ensuring minimal disruption to clinic workflow from the interventions. **Melissa DaCosta**, Director of Patient Experience Operations at RMG, is the patient experience stakeholder. She has worked at RMG for over 10 years and she currently directs daily operations of the Customer Service Department and is responsible for all aspects of patient satisfaction. She is also involved with training and coaching, and assists with orienting new providers in the patient experience culture and will be a great asset in ensuring that the study remains patient centered.

CONSORTIUM CONTRACTUAL ARRANGEMENTS

Describe the proposed research projects that will be performed by subcontracted organizations. Explain the strengths that these partners bring to the overall project.

Subcontract Structure

The proposed project leverages the expertise and leadership of key personnel at UCSD, PAMF, University of Massachusetts Medical School (UMMS)/Meyers Primary Care Institute (MPCI), and Reliant Medical Group (RMG). All organizations are prepared to enter into the necessary inter-organizational agreements, which would be secured through subcontracts from the UCSD.

UMMS/MPCI, an academic institution in Massachusetts, is the other subcontractor with UCSD. Given the study design, system-level research must be carried out at its affiliate practice, RMG. UMMS/MPCI and RMG has its own subcontract for the project.

The subcontract structure would be as follows:

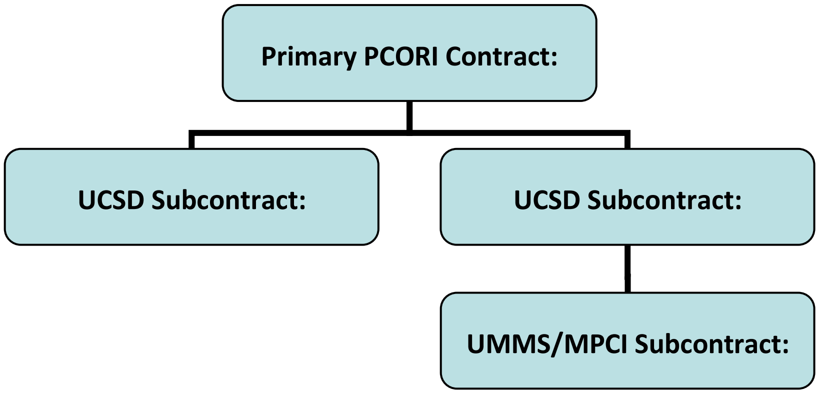

Dr. Tai-Seale had worked at PAMFRI for eight years before moving to UCSD. Dr. Stults was one of the first research team members she had hired during those years. Dr. Tai-Seale has known and collaborated with Dr. Mazor for many years through the HCSRN. Their shared research interest yielded this collaborative project. These strong working institutional relationships, as well as each institution's prior experience in managing federal and non-federal grant awards and contracts, will ensure that all administrative components of the project will occur in a manner that is timely, especially as they are related to project milestones and reporting. Each institution's commitment is provided in their Institutional Letter of Support.

University of Massachusetts Medical School/Meyers Primary Care Institute

The team at UMMS/MPCI will be responsible for patient recruitment-related activities and data-related activities, including patient identification, data collection and management, and regular data monitoring of the clinics, physicians, and participants provided by RMG. Drs. Mazor and Cutrona will coordinate and oversee all aspects of the site, such as managing the local stakeholders, recruiting patients as SPIs, implementing the 3 interventions, and collecting data from RMG.

Dr. Kathleen Mazor, PhD will serve as the RMG Site-PI and will oversee and coordinate the study activities in the Massachusetts site. Dr. Mazor is an experienced psychometrician who has led a multi-site NCI-funded project on health literacy, and is currently leading an AHRQ-funded demonstration project to encourage patients to speak up when they have concerns about their care. She is familiar with multi-site research and will be the primary liaison to interact, communicate, and collaborate with all of the subcontract sites while representing UMMS/MPCI and RMG.

Dr. Sarah Cutrona**,** Assistant Professor of Medicine and a general internist, brings expertise in studying patient, provider, system and social factors and their influences on primary care, and a practicing physician's perspective. Dr. Cutrona has expertise in patient portal-based interventions, and is leading a studies using portal-based messaging to patients to encourage vaccination (with Drs. Mazor and Garber as collaborators) and will be instrumental in knowing how to best administer the aspects of the intervention via the online patient-portal.

Drs. Mazor and Cutrona have well-established working relationships with RMG and will directly collaborate with Drs. Yood and Garber who both hold leadership positions at RMG. They have extensively collaborated with Dr. Garber multiple studies, one of the more relevant on sending portal-based messages to RMG patients.

Reliant Medical Group (RMG)

Reliant Medical Group (RMG) is an integrated healthcare system and the largest private multi-specialty group in Central Massachusetts. RMG operates 25 medical centers and clinics throughout central and eastern Massachusetts staffed by 217 outpatient physicians, including 154 primary care providers (Internal Medicine and Family Practice). RMG provides comprehensive care for more than one million patient visits a year, and serves approximately 320,000 patients. RMG will be the second healthcare system to test the three interventions for the cluster randomized controlled trial.

Dr. Robert Yood is Medical Director of Reliant Medical Group's Research Department, Director of Reliant Medical Group's Division of Rheumatology, and Clinical Professor of Medicine at the University of Massachusetts Medical School. As a senior clinician investigator, Dr. Yood's research activities have focused on the use of administrative databases and electronic health records to evaluate the incidence and treatment of rheumatic diseases. Dr. Yood has collaborated extensively with both Drs. Mazor and Garber on multiple prior studies. He will assist in the recruitment of the clinics and physicians

Dr. Larry Garber is a board certified internist and clinical informaticist, as well as a Health and Human Services (HHS) Office of the National Coordinator (ONC) Health Information Technology (HIT) Fellow and a member of ONC Policy Committee's Interoperability Workgroup. He has extensive experience and expertise in designing and implementing information technology systems. He led the HIMSS Davies Award-winning implementation of Reliant Medical Group's HIMSS Stage 7 Epic Electronic Health Record (EHR) system. Dr. Garber will be instrumental in the technical logistics of integrating the intervention tools into EHR, sending the patient portal messages, and surveys.

10.1.6 SAFETY OVERSIGHT

This trial includes a plan for data and safety monitoring. Drs. Tai-Seale, Stults, and Mazor will conduct data and safety monitoring through monthly data monitoring meetings throughout the study. The results of these data monitoring sessions will be reported to the UCSD, Sutter, and UMass/Reliant IRBs and to the study's project officer at PCORI as part of regular progress reports. Any adverse events will be immediately reported to the institutional IRB.

10.1.7 CLINICAL MONITORING

Not Applicable

10.1.8 QUALITY ASSURANCE AND QUALITY CONTROL

Each clinical site will perform internal quality management of study conduct, data collection, documentation and completion. An individualized quality management plan will be developed to describe a site's quality management.

Quality control (QC) procedures will be implemented beginning with the data entry system and data QC checks that will be run on the database will be generated. Any missing data or data anomalies will be communicated to the site(s) for clarification/resolution.

Following written Standard Operating Procedures (SOPs), the monitors will verify that the clinical trial is conducted and data are generated, documented (recorded), and reported in compliance with the protocol.

10.1.9 DATA HANDLING AND RECORD KEEPING

10.1.9.1 DATA COLLECTION AND MANAGEMENT RESPONSIBILITIES

Data collection is the responsibility of the clinical trial staff at the site under the supervision of the site investigator. All survey data will be collected using online software RedCap. The investigator is responsible for ensuring the accuracy, completeness, and timeliness of the data reported.

10.1.9.2 STUDY RECORDS RETENTION

Study documents should be retained for a minimum of 10 years after the last approval of a marketing application and until there are no pending or contemplated marketing applications in an ICH region or until at least 10 years have elapsed since the formal discontinuation of clinical development of the study intervention. These documents should be retained for a longer period, however, if required by local regulations. No records will be destroyed without the written consent of the sponsor, if applicable. It is the responsibility of the sponsor to inform the investigator when these documents no longer need to be retained.

10.1.10 PROTOCOL DEVIATIONS

A protocol deviation is any noncompliance with the clinical trial protocol, GCP, or MOP requirements. The noncompliance may be either on the part of the patient, MA, PCP, the investigator, or the study site staff. As a result of deviations, corrective actions are to be developed by the site and implemented promptly.

It is the responsibility of the site to use continuous vigilance to identify and report deviations within working days of identification of the protocol deviation, or within working days of the scheduled protocol-required activity. All deviations must be addressed in study source documents. Protocol deviations must be sent to the local IRB per their guidelines. The site PI/study staff is responsible for knowing and adhering to their IRB requirements. Further details about the handling of protocol deviations will be included in the MOP.

10.1.11 PUBLICATION AND DATA SHARING POLICY

A. Describe the ability to reproduce potentially important findings from this research in other data sets and populations.

Reproducibility is a critical component of this study as we are seeking to demonstrate efficacy in a larger number of clinics within UCSD, PAMF, and Reliant Medical Group. As discussed in the dissemination and implementation section, we believe that other organizations with a large patient population, diverse payers and electronic health records may be interested in reproducing the findings from our study. As a result, we will take steps to ensure that researchers have adequate information to test whether our findings are reproducible in other settings beyond participating systems.

B. Describe how you will make available, within nine months of the end of the final year of funding, a complete, cleaned, de-identified copy of the final data set used in conducting the final analyses or your data-sharing plan, including the method by which you will make this data set available, if requested.

We will make a complete, cleaned, de-identified copy of the final data set used in conducting the final analyses available within 9 months after the completion of the study. Researchers interested in replicating our methods and study findings will have full access to the study protocol, samples of intervention prototypes, analytic methods and codebook. We will deliver our final protocol, prototypes, toolkit, codebook documents, and instructions regarding how other researchers can access our study documents to PCORI.

A Data Use Agreement will be implemented for other researchers interested in using our data for replication of research findings or for additional areas of research. We will request that outside investigators discuss their manuscript ideas with the PI (Dr. Tai-Seale) and Site-PIs (Dr. Cheryl Stults and Dr. Kathy Mazor) before proceeding. Furthermore, we will request that manuscripts using data from our project be approved by the PI and Site-PIs prior to submission for publication. Members of this project team may contribute as co-authors when data from this study are used. We will request that manuscripts, abstracts, presentations, and chapters developed by other investigators credit this

study and credit PCORI as the funding source for the data. This process will allow for a central repository and access point for all papers, abstracts, posters, and presentations by any individual or organization using our data.

The study will be registered at www.clinicaltrials.gov and www.socialscienceregistry.org.

C. Propose a budget to cover costs of your data-sharing plan, if requested. *These costs need to be included within the Budget Template.*

None requested in budget.

10.1.12 CONFLICT OF INTEREST POLICY

Actual conflict of interest of persons who have a role in the design, conduct, analysis, publication, or any aspect of this trial will be disclosed and managed. Furthermore, persons who have a perceived conflict of interest will be required to have such conflicts managed in a way that is appropriate to their participation in the design and conduct of this trial. The study leadership in conjunction with PCORI has established policies and procedures for all study group members to disclose all conflicts of interest and will adhere strictly to each institutions Conflict of Interest policy.

10.2 ADDITIONAL CONSIDERATIONS

N/A

10.3 ABBREVIATIONS

The list below includes abbreviations utilized in this template. However, this list should be customized for each protocol (i.e., abbreviations not used should be removed and new abbreviations used should be added to this list).

ANCOVA	Analysis of Covariance
CFR	Code of Federal Regulations
COC	Certificate of Confidentiality
CONSORT	Consolidated Standards of Reporting Trials
CRF	Case Report Form
DCC	Data Coordinating Center
DHHS	Department of Health and Human Services
EC	Ethics Committee
GCP	Good Clinical Practice
HIPAA	Health Insurance Portability and Accountability Act
IB	Investigator's Brochure
ICH	International Conference on Harmonisation
IRB	Institutional Review Board
ISO	International Organization for Standardization
LSMEANS	Least-squares Means
OHRP	Office for Human Research Protections
PCORI	Patient Centered Outcomes Research Institute
PI	Principal Investigator
QA	Quality Assurance
QC	Quality Control
SAP	Statistical Analysis Plan
SOA	Schedule of Activities
SOP	Standard Operating Procedure
UP	Unanticipated Problem
US	United States

10.4 PROTOCOL AMENDMENT HISTORY

The table below is intended to capture changes of IRB-approved versions of the protocol, including a description of the change and rationale. A Summary of Changes table for the current amendment is located in the Protocol Title Page.

**Version**	**Date**	**Description of Change**	**Brief Rationale**

11 REFERENCES

Include a list of relevant literature and citations for all publications referenced in the text of the protocol. Use a consistent, standard, modern format, which might be dependent upon the required format for the anticipated journal for publication (e.g., N Engl J Med, JAMA, etc.). The preferred format is International Committee of Medical Journal Editors (ICMJE). Include citations to product information such as manufacturer's IB, package insert, and device labeling.

Examples:

- ***Journal citation***
  Veronesi U, Maisonneuve P, Decensi A. Tamoxifen: an enduring star. J Natl Cancer Inst. 2007 Feb 21;99(4):258-60.
- ***Whole book citation***
  Belitz HD, Grosch W, Schieberle P. Food chemistry. 3rd rev. ed. Burghagen MM, translator. Berlin: Springer; 2004. 1070 p.
- ***Chapter in a book citation***
  Riffenburgh RH. Statistics in medicine. 2nd ed. Amsterdam (Netherlands): Elsevier Academic Press; c2006. Chapter 24, Regression and correlation methods; p. 447-86.
- ***Web Site citation***
  Complementary/Integrative Medicine [Internet]. Houston: University of Texas, M.D. Anderson Cancer Center; c2007 [cited 2007 Feb 21]. Available from: http://www.manderson.org/departments/CIMER/.
- ***Electronic Mail citation***
  Backus, Joyce. Physician Internet search behavior: detailed study [Internet]. Message to: Karen Patrias. 2007 Mar 27 [cited 2007 Mar 28]. [2 paragraphs]
- ***References to package insert, device labeling or investigational brochure***
  Cite date accessed, version number, and source of product information.

1. Braddock III CH, Edwards KA, Hasenberg NM, Laidley TL, Levinson W. Informed decision making in outpatient practice: time to get back to basics. *Jama.* 1999;282(24):2313-2320.
2. Zikmund-Fisher BJ, Couper MP, Singer E, et al. The DECISIONS study: a nationwide survey of United States adults regarding 9 common medical decisions. *Medical Decision Making.* 2010;30(5 Suppl):20S-34S.
3. Frosch DL, May SG, Rendle KAS, Tietbohl C, Elwyn G. Authoritarian Physicians And Patients' Fear Of Being Labeled 'Difficult' Among Key Obstacles To Shared Decision Making. *Health Affairs.* 2012;31(5):1030-1038.
4. Adams JR, Elwyn G, Légaré F, Frosch DL. Communicating with physicians about medical decisions: a reluctance to disagree. *Arch Intern Med.* 2012;172(15):1184-1186.
5. Stewart MA. What is a successful doctor-patient interview? A study of interactions and outcomes. *Social Science & Medicine.* 1984;19(2):167-175.
6. Stewart M, Brown J, Donner A, et al. The impact of patient-centered care on outcomes. *J Fam Pract.* 2000;49(9):796-804.

7. Bertakis K, Callahan E, Helms L, Azari R, Robbins J, Miller J. Physician practice styles and patient outcomes: differences between family practice and general internal medicine. *Medical Care.* 1998;36(6):879-891.
8. Levinson W. Physician-patient communication: A key to malpractice prevention. *JAMA.* 1994;272(20):1619.
9. Kaplan SH, Greenfield S, Ware Jr JE. Assessing the effects of physician-patient interactions on the outcomes of chronic disease. *Medical Care.* 1989;27(3):S110.
10. Greenfield S, Kaplan SH, Ware Jr JE, Yano EM, Frank HJ. Patients' participation in medical care. *Journal of general internal medicine.* 1988;3(5):448-457.
11. Rao J, Anderson L, Inui T, Frankel R. Communication Interventions Make A Difference in Conversations Between Physicians and Patients: A Systematic Review of the Evidence. *Medical Care.* 2007;45(4):340-349.
12. Adams JR, Elwyn G, Légaré F, Frosch DL. Communicating with physicians about medical decisions: a reluctance to disagree. *Arch. Intern. Med.* 2012;172(15):1184-1186.
13. Tai-Seale M, Foo PK, Stults CD. Patients With Mental Health Needs Are Engaged In Asking Questions, But Physicians' Responses Vary. *Health Affairs.* 2013;32(2):259-267.
14. Alegria M, Polo A, Gao S, et al. Evaluation of a patient activation and empowerment intervention in mental health care. *Medical Care.* 2008;46(3):247-256.
15. Tai-Seale M, Elwyn G, Wilson C, et al. Promoting Shared Decision Making in Primary Care Office Visits: Comparing a Novel Multidimensional Intervention with an Existing Patient Activation Intervention. *Health Affairs.* 2016;in press.
16. Shepherd HL, Barratt A, Jones A, et al. Can consumers learn to ask three questions to improve shared decision making? A feasibility study of the ASK (AskShareKnow) Patient–Clinician Communication Model® intervention in a primary health‐care setting. *Health Expectations.* 2015.
17. Joint Commission on Accreditation of Healthcare Organizations. *What did the doctor say?: Improving health literacy to protect patient safety.* Joint Commission on Accreditation of Healthcare Organizations; 2007.
18. Schillinger D, Keller D. The Other Side of the Coin: Attributes of a Health Literate Health Care Organization. Paper presented at: How Can Health Care Organizations Become More Health Literate?:: Workshop Summary2012.
19. Andersson S, Mattsson B. Features of good consultation in general practice: is time important? *Scand J Prim Health Care.* 1994;12(4):227-232.
20. Marvel MK, Epstein RM, Flowers K, Beckman HB. Soliciting the patient's agenda: have we improved? *Journal of American Medical Association.* 1999;281(3):283-287.
21. Peltenburg M, Fischer J, Bahrs O, Van Dulmen S, van den Brink-Muinen A. The unexpected in primary care: a multicenter study on the emergence of unvoiced patient agenda. *The Annals of Family Medicine.* 2004;2(6):534.
22. White J, Levinson W, Roter D. "Oh, by the way ...": the closing moments of the medical visit. *Journal of General Internal Medicine.* 1994;9(1):24-28.
23. Elwyn G, Barr PJ, Grande SW, Thompson R, Walsh T, Ozanne EM. Developing CollaboRATE: A fast and frugal patient-reported measure of shared decision making in clinical encounters. *Patient Education and Counseling.* 2013.

24. Lerman CE, Brody DS, Caputo GC, Smith DG, Lazaro CG, Wolfson HG. Patients' perceived involvement in care scale. *Journal of General Internal Medicine.* 1990;5(1):29-33.
25. Manary MP, Boulding W, Staelin R, Glickman SW. The Patient Experience and Health Outcomes. *New England Journal of Medicine.* 2013;368(3):201-203.
26. Street RL, Jr., Makoul G, Arora NK, Epstein RM. How does communication heal? Pathways linking clinician-patient communication to health outcomes. *Patient Educ Couns.* 2009;74(3):295-301.
27. Stewart MA. Effective physician-patient communication and health outcomes: a review. *CMAJ: Canadian Medical Association Journal.* 1995;152(9):1423.
28. Tai-Seale M. Physician Management of Demand at the Point of Care. In: Culyer AJe-i-c, ed. *Encyclopedia of Health Economics.* Vol Volume 3: Elseview; 2014:61-67.
29. Kahneman D. *Thinking, Fast and Slow.* Farrar, Straus and Giroux; 2013.
30. Cohn A, Fehr E, Marechal MA. Business culture and dishonesty in the banking industry. *Nature.* 2014;516(7529):86-89.
31. Daetwyler C, Cohen D, Gracely E, Novack D. eLearning to Enhance physician patient communication: A pilot test of "doc.com" and "WebEncounter" in teaching bad news delivery. *Medical Teacher.* 2010;32:e381–e390.
32. Hibbard JH, Stockard J, Mahoney ER, Tusler M. Development of the Patient Activation Measure (PAM): conceptualizing and measuring activation in patients and consumers. *Health Services Research.* 2004;39(4 Pt 1):1005-1026.
33. Ajzen I. *From intentions to actions: A theory of planned behavior.* Berlin, Heidelberg: Springer; 1985.
34. Ajzen I. Constructing a Theory of Planned Behavior Questionnaire. http://www.people.umass.edu/aizen/pdf/tpb.measurement.pdf. Accessed 12 February 2016.
35. Chisholm MA, Williamson GM, Lance CE, Mulloy LL. Predicting adherence to immunosuppressant therapy: a prospective analysis of the theory of planned behaviour. *Nephrology, dialysis, transplantation : official publication of the European Dialysis and Transplant Association - European Renal Association.* 2007;22(8):2339-2348.
36. DiMatteo MR, Sherbourne CD, Hays RD, et al. Physicians' characteristics influence patients' adherence to medical treatment: results from the Medical Outcomes Study. *Health Psychology.* 1993;12(2):93-102.
37. Ware J, Jr., Kosinski M, Keller S. A 12-Item Short-Form Health Survey: construction of scales and preliminary tests of reliability and validity. *Medical Care.* 1996;34(3):220-233.
38. Consumer Assessment of Healthcare Providers and Systems. CAHPS Clinician & Group Survey Adult Primary Care Questionnaire. 2007; https://www.cahps.ahrq.gov/CAHPSkit/files/351a_Adult_Prim_Eng.pdf. Accessed October, 2007.
39. Centers for Disease Control and Prevention. Health Information National Trends Survey. 2016; https://www.healthypeople.gov/2020/data-source/health-information-national-trends-survey. Accessed Febuary 5, 2016.

40. Makoul G, Krupat E, Chang CH. Measuring patient views of physician communication skills: development and testing of the Communication Assessment Tool. *Patient Educ Couns.* 2007;67(3):333-342.
41. Tai-Seale M, Olson CW, Li J, et al. Electronic Health Record Logs Indicate That Physicians Split Time Evenly Between Seeing Patients And Desktop Medicine. *Health Aff (Millwood).* 2017;36(4):655-662.
42. Schumaker RE, Lomax RG. *A Beginner's Guide to Structural Equation Modeling.* Third ed: Routledge; 2010.
43. Mazor KM, Roblin DW, Williams AE, et al. Health literacy and cancer prevention: two new instruments to assess comprehension. *Patient Educ Couns.* 2012;88(1):54-60.
44. Mazor KM, Rogers HJ, Williams AE, et al. The Cancer Message Literacy Tests: psychometric analyses and validity studies. *Patient Educ Couns.* 2012;89(1):69-75.
45. Mazor KM, Williams AE, Roblin DW, et al. Health literacy and pap testing in insured women. *Journal of cancer education : the official journal of the American Association for Cancer Education.* 2014;29(4):698-701.
46. Mazor KM, Calvi J, Cowan R, et al. Media messages about cancer: what do people understand? *J Health Commun.* 2010;15 Suppl 2:126-145.
47. Mazor KM, Rubin DL, Roblin DW, et al. Health literacy-listening skill and patient questions following cancer prevention and screening discussions. *Health Expect.* 2015.
48. Mazor K, Smith K, Fisher K, Gallagher T. "Speak Up!" Addressing the paradox plaguing patient-centered care. *Annals of Internal Medicine.* 2016.
49. Cutrona S, Sreedhara M, Goff S, et al. Improving rates of influenza vaccination through EHR Portal Messages, Interactive Voice Recognition Calls and Patient-Enabled EHR updates: Protocols for a Randomized Control Trial. *JMIR Res Protoc.* forthcoming.
