# Supplementary material for: Patient-Clinician Communication Interventions Across Multiple Primary Care Sites: A Cluster Randomized Clinical Trial
Source: JAMA Health Forum. 2024 Dec 13;5(12):e244436. doi: 10.1001/jamahealthforum.2024.4436 (PMC11645648; doi:10.1001/jamahealthforum.2024.4436)
Supplement: Supplement 2. — Statistical Analysis Plan [file jamahealthforum-e244436-s002.pdf]

Supplement 1

eMethods. Statistical Analysis Plan

1. Summary of Study Design

Phase 1: Phase 1 includes intervention and survey development work to take place prior to the randomized clinical trial (RCT). The tasks include development of the following 6 components which we describe in further detail below: (1) OPEN High Touch; (2) OPEN High Tech; (3) ASK posters; (4) Online messaging content and system; (5) survey instruments; (6) English to Spanish translation of our documents which will take place at UCSD.

OPEN High Touch intervention - The **High Touch** intervention will be modeled after the Open Communication intervention developed in the pilot which contained three components: (a) a one question pre-visit survey delivered through the patient portal of the electronic health record (EHR), asking patients what they most want to discuss with their physician in the upcoming visit; (b) an animated video for patients providing coaching on how to best prepare for their upcoming visits and get the most from the visits; (See http://bcove.me/fevffx4w for the version used in the pilot; and (c) Standardized Patient Instructor (SPI) providing communication coaching for physicians on how to incorporate what matter most to patients in the visit, with empathy, and clarity. We have adapted the (1) the existing paper version of the Visit Companion Booklet to be a one item questionnaire (see attached) and (2) the existing in-person SPI training of physicians and medical assistants to reflect revised workflow with the previsit questionnaire and after visit summary (AVS), and (3) updated the video explaining the OPEN intervention to participants to illustrate these new workflows. We have also revised the standardized patient instructor manual developed in the pilot study.

OPEN High Tech intervention - For the **High Tech** arm, the patient components of the intervention will be identical to the patient components of the High Touch arm (i.e., the pre-visit survey and patient coaching video). The difference will be in the primary care provider (PCP) training: we will replace the in-person SPI with a mobile app with embedded audio and video vignettes demonstrating the communication challenges (e.g., patient with a big list of issues, patients who resist physician recommendations, and patients who disagree with physician) and recommended strategies.[13] A mobile app offers several advantages, including being accessible at a convenient time for busy providers, being easily disseminated, and easily updated. The app will be interactive, posing questions to learners in association with video vignettes and asking learners to answer how they would handle the situation. We will start with the idea of building a set of short mobile modules that mirror the High Touch approach, honing skills on acknowledging patient's agenda, negotiate a joint agenda, invite patient to teachback and incorporate it in the After Visit Summary in the EHR. This is currently under development at UCSD.

ASK intervention - The **ASK** intervention is intended to activate patients by encouraging them to ask three questions during their primary care visit: (1) What are my options? (2) What are the possible benefits and risks of each option? (3) How likely are each of the benefits and risks to happen to me? These questions are printed on two types of posters with identical text but different graphics and placed in exam rooms used by providers in clinics randomized to the ASK arm of the trial.

Phase 2: Phase 2 covers the trial recruitment, and three waves of data collection. Clinics from the 3 systems will be randomized 1:1:1 into our three treatment arms, on average N=5 primary care providers will be recruited per clinic. A total of n = 50 patients will be entered into the study per provider. Prior to the start of the RCT we will collect baseline (T0) data to allow measurement of PCP performance prior to the trial with n = 10 patients surveyed per PCP at baseline. Patients participating in the T0 phase will provide only post-visit ratings of their encounters; we will not collect other outcome data or clinical indicators for these patients. For all patients in the intervention phase (~40/PCP), we will be collecting information at two time points: 1) immediately post-encounter (T1); and 2) three months post-encounter (T2). We will further sample the top 5% high users of services after the intervention and review their medical records including the indexed visit and subsequent services that had occurred within four weeks after the indexed visit. The chart review will enable us to decipher the reasons for high volume of services after the indexed visit.

2. Baseline Characteristics

2.1 PCPs:

Variable or Vector of Variables Definition	Notes	Name (UCSD & Reliant)
Age, (Age Group)	From Post Intervention Survey	
Race/Ethnicity, N (%)	From Post Intervention Survey	
Gender, N(%)	From Post Intervention Survey	
Number of Patient Calls Received*	Unique patients per day	PtCallsReceived
Number of Patient-Initiated Inbasket Messages Received*	Unique patients per day	PtMsgreceived
Minutes Spent in Inbasket*	Per day	inbasketminutes
Number of Quick Actions Created*	Signal data	NQuickActionscreated
Number of Quick Actions Used*	Signal data	NQuickActionsUsed
AVS contains Patient Instruction or not*	HyperSpace	AVShasPatientInstruction
Time in Progress Notes*	Signal data	timeinnotes
% Encounter Same Day Closure*	Signal data	Pctencountersamedayclosure
Length of office visits	Difference between log-in and log-out time	VisitLength
PCP Length of Practice	Length of practice of PCP since residence training (from Post Intervention Survey)	Prov_Length_Work

(*) Indicates window of one year from 11/1/2018 – 10/31/2019

2.2 Medical Assistants/Licensed Vocational Nurses (MAs/LVNs):

Variable or Vector of Variables	Notes
Age, (in Age Group)	From Post Intervention Survey
Race/Ethnicity, N (%)	From Post Intervention Survey
Gender, N(%)	From Post Intervention Survey
MA or LVN	Rooming staff is MA or LVN
MA/LVN Length of Work	Length of work of MA/LVN at current organization

2.3 Patients:

Variable or Vector of Variables	Notes
Age, Mean (SD)<br>Median [IQR]	From EHR
Race/Ethnicity, N (%)	From Post Intervention Survey
Gender, N(%)	From EHR
Socioeconomic Status: Median Income (Census Block)	Patient's zipcode-associated census block average income
Socioeconomic Status: Poverty (Census Block)	Patient's zipcode-associated census block %below federal poverty level
Education (Census Block)	Patient's zipcode-associated census block %high school/college grad
Medications	Class and number of prescription medications in each class
Variable or Vector of Variables	Notes
Diagnoses	ICD9/10 codes
Charlson Comorbidity Index (CCI)	*may be modified*
Number of MyChart/MyHealth Online messages sent to PCP	A proxy measure for the volume of use of medical resources
Number of telephone encounters	A proxy measure for the volume of use of medical resources
Number of outpatient office visits	A proxy measure for the volume of use of medical resources

3. Study Enrollment and Dropout (Participation)

Study enrollment will be reported by health system and study arm, separately for clinics, PCP's, MA/LVN's, and patients.

Dropout is defined as someone who had signed the informed consent to participate in the study but did not participate before the end of the study.

For PCP's, the dropout/loss to follow-up will be reported as N (%), by health system and study arm, together with a detailed list of the individual PCP dropouts, and the reason for dropping out.

PCP/MA/LVN Participation

	**UCSD**				**Sutter**				**Reliant**			
	**All**	**Ask**	**Open High Tech**	**Open High Touch**	**All**	**Ask**	**Open High Tech**	**Open High Touch**	**All**	**Ask**	**Open High Tech**	**Open High Touch**
Clinics, N (ID)	**6**	2 (7,8)	2 (11,12)	2 (9,10)	**9**	3 (17,19,21)	3 (15,16,18)	3 (13,14,20)	**6**	2 (5,6)	2 (2,3)	2 (1,4)
Enrolled MA/LVNs, N (ID)												
Enrolled PC Providers, N (ID)												
PC Providers with ≥ 8 baseline patients, N												

	UCSD				Sutter				Reliant			
	**All**	**Ask**	**Open High Tech**	**Open High Touch**	**All**	**Ask**	**Open High Tech**	**Open High Touch**	**All**	**Ask**	**Open High Tech**	**Open High Touch**
PC Providers with 0 follow-up patients, N												
PC Providers with ≥ 20(?) follow-up patients												

Patient Participation

	Baseline	Post-Intervention
Signed Consent Form		
Completed Visit		
Completed 3-month follow-up	N/A	

Reasons for dropping out will be recorded as they are available.

4. Outcome Measures

4.1 Primary Outcome and Comparisons: CollaboRATE

CollaboRATE is a validated 3-item patient engagement measure that captures patient perceptions of communication and decision-making during the appointment. The 3 questions are: "How much effort was made to help you understand your health issues?" "How much effort was made to listen to the things that matter most to you about your health issues?" "How much effort was made to include what matters most to you in choosing what to do next?" The participants score each question from 0-9 (Likert scale), with 9 being the highest score. The primary outcome is whether or not the patient gave the top score of 9 on all three questions.

The primary comparison is that of OPEN-High Tech vs. ASK, and it will be performed at the a=0.05 level, as a superiority test. The OPEN-High Tech vs. ASK was chosen as the primary comparison because OPEN-High Tech is a potentially more scalable, cost-effective intervention, with great potential for implementation in real world practices.

The secondary comparisons are of OPEN-High Touch vs. ASK and OPEN-High Tech vs. OPEN-High Touch. The High Tech vs. High Touch comparison will be a non-inferiority comparison with a non-inferiority margin of 5%, performed as a secondary analysis at level a=0.025 one-sided, whereas the Touch vs. ASK comparison will be a separate secondary analysis performed as a superiority comparison.

No overall, 3-arm comparison will be done.

The primary outcome (binary) at the patient level will be compared between arms using mixed-effects logistic regression. This model will include both baseline and follow-up time points. The model will include a term for time point (baseline vs. follow-up), treatment arm, and their interaction. A significant time-by-treatment interaction in a given comparison between arms indicates a difference in intervention effects between arms. This hierarchical clustering model will include a random effect for PCP and a random effect for clinic, in order to account for within-PCP and within-clinic correlations. (Since each participant is evaluated at a single visit, baseline or follow-up, no participant random effect is necessary.) Intent to treat analysis will be employed such that all data will be analyzed based on the intervention arm in which patients are located without regard to whether the intervention was fully carried out on them or not. PCP dropout

will be ignored (i.e., “missing = missing” analysis), with further sensitivity analyses comparing characteristics of the dropout PCP’s with those not dropping out.

4.2 Secondary Outcome and Comparisons: Facilitate

The Facilitation subscale is a validated 5-item measure of patient perceptions of how well the physician facilitated their involvement in decision making. The 5 items are: “ My doctor (1) asked me whether I agree with his/her decisions; (2) gave me a complete explanation for my medical symptoms or treatment; (3) asked me what I believe is causing my medical symptoms; (4) encouraged me to talk about personal concerns related to my medical symptoms; (5) encouraged me to give my opinion about my medical treatment.” The participants score each question from 0-9 with 0 corresponding to “Definitely Disagree” and 9 corresponding to “Definitely Agree.” The primary outcome is whether or not the patient gave a score of 9 for each of the 5 questions.

The primary and secondary comparisons of the secondary outcome are identical to those of the CollaboRATE primary outcome. The levels at which the comparisons will be performed are also the same as those from the primary outcome comparisons. Like the primary outcome, no overall, 3-arm comparison will be done.

The primary outcome (binary) at the patient level will also be compared between arms using mixed-effects logistic regression. This model will include both baseline and follow-up time points. The model will include a term for time point (baseline vs. follow-up), treatment arm, and their interaction. A significant time-by-treatment interaction in a given comparison between arms indicates a difference in intervention effects between arms. This hierarchical clustering model will include a random effect for PCP and a random effect for clinic, in order to account for within-PCP and within-clinic correlations. (Since each participant is evaluated at a single visit, baseline or follow-up, no participant random effect is necessary.) Intent to treat analysis will be employed such that all data will be analyzed based on the intervention arm in which patients are located without regard to whether the intervention was fully carried out on them or not. PCP dropout will be ignored (i.e., “missing = missing” analysis), with further sensitivity analyses comparing characteristics of the dropout PCP’s with those not dropping out.

4.3 Intervention Exposure

	**Variable or Vector of Variables***	**Notes**
OPEN High Touch	Number of Training Sessions Attended	N of Part A, N of Part B
	Used OPEN SmartPhrase or not	‘OPENSmartPhrase’ used
OPEN High Tech	Used OPEN SmartPhrase or not	‘OPENSmartPhrase’ used
	Time in module (PCP, MA/LVN)	Length of time spent per module
	Time in App (PCP, MA/LVN)	Length of total time spent in app
ASK	Poster visibility for corresponding clinic	The number of other posters on the wall in the exam room. (Reliant possibly 0; Sutter N TBD from photos; UCSD N TBD from photos.)

(*) All variables are on the PCP or MA/LVN level (poster visibility applies to all PCP/MA/LVNs within corresponding clinic)

4.4 Additional Secondary Analyses

a) CollaboRATE and Facilitate as Continuous Outcomes

Further analysis of the primary and secondary endpoints will be performed with continuous outcomes of the respective endpoints and compared between arms using linear mixed-effects regression. The continuous outcome will be the cumulative score of the CollaboRATE and Facilitate measures. The models will include both baseline

and follow-up time points. The models will include a term for time point (baseline vs. follow-up), treatment arm, and their interaction. A significant time-by-treatment interaction in a given comparison between arms indicates a difference in intervention effects between arms. These hierarchical clustering models will include a random effect for PCP and a random effect for clinic, in order to account for within-PCP and within-clinic correlations. (Since each participant is evaluated at a single visit, baseline or follow-up, no participant random effect is necessary.) Intent to treat analysis will be employed such that all data will be analyzed based on the intervention arm in which patients are located without regard to whether the intervention was fully carried out on them or not. PCP dropout will be ignored (i.e., "missing = missing" analysis), with further sensitivity analyses comparing characteristics of the dropout PCP's with those not dropping out.

b) Recommend as Net Promoter Score (NPS)

We will analyze the item "likelihood of recommending this care provider to others" as a net promoter score (NPS), with response option 5 coded as "promoter", 4 as "neutral", and 1-3 as detractor".[39] The analysis will use longitudinal ordinal logistic regression, with a time-by-treatment interaction and random effect for PCP, and effect size reported in terms of odds ratios (OR) of higher vs lower NPS; for treatment comparisons the effect size is a ratio of odds ratios (ROR), comparing treatment arms in terms of ORs of higher vs lower NPS, for follow-up vs baseline. P-values will be based on the likelihood ratio test. An additional analysis using longitudinal linear mixed-effects models treating NPS as a numeric variable, with values +1 (promoter), 0 (neutral), and –1 (detractor), will also be included.

c) COVID-Adjusted[a]

Adjusted analyses will also examine the impact of the COVID-19 pandemic on the treatment effects. This will be done by examining treatment effects on primary outcomes in stratified analyses, with strata defined by classifying the visits as occurring up to March 13, 2020 or after this date, when COVID-19 was declared a national emergency.

[a]*The original Protocol had been updated during COVID upon request from PCORI as they needed to assess the viability of the study due to challenges imposed by the pandemic.*

d) Baseline-Adjusted Analysis

Analysis of the primary and secondary endpoints will be performed adjusting for baseline characteristics of participating PCPs, MA/LVNs, and patients (age, gender, race/ethnicity, SVI). This will allow us to measure the effect of the treatment while controlling for characterized differences in participants. The models will include both baseline and follow-up time points. The models will include a term for time point (baseline vs. follow-up), treatment arm, and their interaction. A significant time-by-treatment interaction in a given comparison between arms indicates a difference in intervention effects between arms. These hierarchical clustering models will include a random effect for PCP and a random effect for clinic, in order to account for within-PCP and within-clinic correlations.

e) Moderating Effects of Covariates on Treatment

Analysis of the primary and secondary endpoints will be performed controlling for moderating effects of PCP covariates (e.g. sex, race, length of practice, etc.) on the treatment effect. These will be measured separately by treatment arm and overall with a full model including all treatment arms. The models will include both baseline and follow-up time points. The models will include a term for time point (baseline vs. follow-up), treatment arm, and their interaction. A significant time-by-treatment interaction in a given comparison between arms indicates a difference in intervention effects between arms. These hierarchical clustering models will include a random effect for PCP and a random effect for clinic, in order to account for within-PCP and within-clinic correlations.

f) Planned Sensitivity Analysis

As a sensitivity analysis, a structural equations method (SEM) employing the actual scores given by patients will also be applied. We will also use an alternative specification which treats CollaboRATE and Facilitation as latent variables which are predicted by observed variables reflected by the actual score given by patients. A special case of SEM, known as a Multiple Indicators Multiple Causes (MIMIC) model, will be used to examine the effect of the intervention on latent variables CollaboRATE or Facilitation. Prior to estimating the MIMIC model, we will conduct a confirmatory factor analysis (CFA) to validate the structure of CollaboRATE and Doctor Facilitation. In the pilot study, the one-factor CFA had 3 items loading onto the single factor CollaboRATE, with the Cronbach's alpha=0.96. The one-factor CFA had 5 items loading onto the single factor Facilitation, with the Cronbach's alpha=0.85. This model and the subsequent MIMIC model will account for clustering of patients within physicians and physicians within clinics by permitting the errors to be correlated within clusters. This and the subsequent MIMIC model will be estimated using full information maximum likelihood (FIML) estimation which allows all available data to be used.

Following the CFA, we will estimate the MIMIC model which consists of the measurement model discussed in the previous paragraph and a structural part, where the latent variable CollaboRATE or Facilitation will be predicted by observed variables on intervention groups and patient demographics. The models will control for patient level demographic variables including age, sex, race/ethnicity, and education, as well PCP variables such as age, sex, and race/ethnicity.

g) 3-month Survey

The survey of patients 3 months after the indexed visit is to measure change over time of secondary outcomes, including clinical and utilization outcomes, and adherence to treatment plans. Repeated measures will enable us to determine whether there is a lasting effect of the intervention beyond the initial improvement in communication. In addition, it will allow us to measure whether there are meaningful changes in utilization and clinical outcomes after intervention.

Diabetes

To measure utilization, we will focus on patients with Diabetes. We estimate that 6.3% of our primary care patients have diabetes, and 75% of these patients will not have had their hemoglobin A1c and LDL tested within 6 months. If we can reduce this percentage to 54%, we will have sufficient power to detect the change (power=0.8, alpha=0.05, one-sided test). For this estimate, two-level clustering (at the provider and clinic level) with ICCs of 0.02 was assumed, and approximate sample sizes within each arm were used. Because we expect less than 25% of people with diabetes to have A1c and LDL measurements, we will not have sufficient power to detect changes in A1c and LDL between time periods. However, we will record these and look for trends.

Hypertension

Our main clinical outcome is controlled hypertension. From preliminary data from a random sample of primary care patients, we estimate that approximately 25% of our patients will have hypertension, and of those, 30% will be uncontrolled. Using a one-sided test, we expect to be able to detect an 11 percentage-point decrease (30% to 19%) from visit 1 to 3-month follow-up in the percentage of patients with uncontrolled hypertension (power=0.8, alpha=0.05). Not knowing the number of patients who will have repeat blood pressure measures between the index visit and 3-month follow up, we will examine the data and report clinically outcomes accordingly.

We also plan to measure quality of life using the VR-12 with a standardized scale. Assuming a mean of 50 and an SD of 10, we will have 80% power to detect a difference of 1.7 units, and alpha=0.05, ICC=0.02.

Confidence

The patients' confidence in their ability to follow the treatment plan decided upon in collaboration with their doctor is measured both at the indexed visit in the immediate post-visit survey as well as in the 3-month follow-up survey. This allows for the analysis of this confidence using a longitudinal analysis with only two time points. We will use a general linear model for longitudinal data.

This will allow us to determine whether the Arm a patient is in informs the trajectory of confidence scores over those two time points. The specific test will be whether the coefficient for time point by arm is significant in the model. In this model we will also control for site and doctor to explain some of the variation in response.

Intention

Intention is patient's answer to the question of their intention to follow through with the plan that they and their doctor had made. We will use the average score given on intention to follow through with the plan set forth by a patient and PCP. This average will be used as an outcome in a linear mixed effects model, controlling for both PCP, clinic and site.

h) PCP Outcomes

Additional analysis of PCP outcomes before and after intervention (measured between 11/1/18 and the last date of intervention at each relevant site) will be performed to quantify the effect of intervention. These outcomes consist of baseline characteristics such as the number of patient calls received, the number of patient-initiated Inbasket messages received, and the total minutes spent in Inbasket.

Summary:

Outcome Type	Measure	T1 Immediate post-encounter	T2 3 months post-encounter	T3 6 months post-encounter	Data Source
Patient reported experience with care	CollaboRATE[23]	X			Patient survey
	Facilitation[24]	X			Patient survey
Action plan,<br>patient reported confidence/intention to adhere<br>Adherence	Action plan<br>CONFIDENCE[39]<br>INTENTION[34,35] to adhere<br>Adherence to action plans	X<br>X<br>X	 X<br>X<br>X<br>X		Patient survey
Clinical indicators	VR12<br>Blood pressure<br>A1c<br>LDL	X<br>X<br>X<br>X	X<br>X<br>X<br>X	 X<br>X<br>X	Patient survey<br>EHR<br>EHR<br>EHR
Service use, impact on healthcare system	Patient-initiated calls, e-messages, office visits (televisits[a] included) after indexed visits			X	EHR – structured fields, access log, Physician Efficiency Profile, in the 6 months after the indexed visit.

[a] added after the COVID pandemic.

4.5 Missing Data

Missing data could come in three forms: missing patient reported outcomes, missing PCP reported outcomes, or missing EHR data. Missing data on these outcomes will be due to one of three situations: (1) non-response to patient survey; (2) non-response to PCP survey; and (3) returned incomplete questionnaire, in the event that some respondents leave some items in surveys unanswered.

We plan to analyze the non-responders throughout the course of data collection according to available EHR and administrative data on patients and PCPs. Careful monitoring of missing data will allow us to consider the related changes in interpretability of our estimated effects. To assure maximal participation, we will collect survey data via the web, with financial incentives for completing the surveys. With our survey tracking system, we will identify subjects who are "out of window" for their return of their survey and all respondents who only partially complete a returned survey. In either case, we will attempt to contact them to obtain the information via the patient portal again. After three

attempts, we will treat their survey as incomplete or missing, record it as such, and a judgment will be made as to whether the completed questions provide enough information to impute an overall score. If so, we will employ methods of multiple imputation to fill in the missing data for partial surveys. Comparisons of participants with and without missing data will also be provided.

We do not anticipate that missing survey data will be informative with respect to the treatments (Open High Touch, Open High Tech, ASK), and will have comparative EHR clinical or administrative data for them to determine whether selection bias is relevant to the evaluation of the change in patient reported outcomes. If necessary, we will profile the respondent subgroup and restrict interpretation of patient-reported outcome results to this population. We will prepare an annual report of all missing data including an assessment of bias.

Regarding the possibility of missing EHR-sourced data, while we do not anticipate a significant issue of missingness in outcome measurements, some missing data in baseline and demographic characteristics is likely. We will conduct a by-variable evaluation of missing data in this setting and determine in each case how missing data should be treated in our analyses.

Summary of Changes in Statistical Analysis Plan

To: Gyasi Moscou-Jackson, PhD, MHS, RN
From: Ming Tai-Seale, PhD, MPH, Florin Vaida PhD
RE: **Summary of Changes in Study Design for "OPEN and ASK Study"**
Date: June 15, 2018

We appreciate your approval of the proposed changes in the study design in order to increase statistical power. We describe the changes and the reasons for the changes below.

1. **Hierarchical approach to group comparison, consistent with a 3-arm study design:**
   The <u>primary comparison</u> is that of <u>OPEN-High Tech vs. ASK</u>, and it will be performed at the α=0.05 level, as a superiority test.

   The <u>secondary comparisons</u> are of OPEN-High Touch vs. ASK and OPEN-High Tech vs. OPEN-High Touch. The High Tech vs. High Touch comparison will be a non-inferiority comparison with a non-inferiority margin of 5%, performed as a secondary analysis at level α=0.025 one-sided, whereas the Touch vs. ASK comparison will be a separate secondary analysis performed as a superiority comparison trying to detect a 5% difference (77% vs 72%), at level α=0.05 two-sided. The power of these two additional pairwise comparisons is included in Tables 2 and 3 in the Appendix at the end of this document. The conclusion is that the 10+40 redistribution will provide enough power (.78 to .84) to detect a 5% difference. No overall, 3-arm comparison will be done.

   The OPEN-High Tech vs. ASK was chosen as the primary comparison because OPEN-High Tech is a potentially more scalable intervention, with lower costs and great potential for implementation in real world practices.

2. **Increasing follow-up sample size and reducing baseline sample size:**
   Instead of recruiting 25 patients at baseline and 25 patients at follow-up for each PCP, we recruit instead 10 patients at baseline and 40 patients at follow-up. The proposed change will recruit the same number of patients per PCP (50), but with a greater weight placed on follow-up.

   The two changes outlined above are justified by the findings from the pilot study (Tai-Seale et al 2016). Additional analyses of the pilot study data revealed that the estimated within-PCP intra-class correlation (ICC) between baseline and follow-up outcomes was negligible (ICC=0.000004 for *CollaboRATE*=9, ICC=0.00000005 for *Facilitate*=9). In the absence of within-PCP ICC, the baseline samples do not help reduce variability, making the statistical analysis just as effective as the direct comparison of the follow-up time points. In other words, the baseline samples have no statistical value if ICC is again negligible. This is confirmed in our statistical simulations.

However, if in our study it turns out that the within-PCP ICC is positive (but small), the baseline samples will improve power. For this reason, we propose a middle way: instead of distributing the (baseline, follow-up) patients as (25, 25) as in the original design, or (0, 50) as optimal under within-PCP ICC=0 assumption, we propose to use 10 baseline + 40 follow-up patients. This will further increase the power of the study comparisons, equivalent to an increase in sample size by 60% compared to the (25, 25) original design.

3. **Effect size to be detected in the primary analysis is 5% points.**
The pilot study showed a difference of 8% on our primary endpoint (CollaboRATE=9) between High Touch and Usual care (74.7% vs 66.7%) but of approximately 3% between High Touch and ASK (74.7% vs 72.0%), see Table 2 in Tai-Seale et al. (2016). Upon review, the 10% difference in the original proposal between High Tech and ASK, and between High Touch and ASK may be optimistic, even though not impossible, given that the confidence interval of findings from the pilot study included 10% and the potentially greater diversity in clinical practice patterns across 3 health systems. A 5% anticipated difference may be moderately realistic, while a 3% anticipated difference is conservative. Our calculations show that with the proposed changes in study design we have enough power to detect a difference of 5% between High Tech and ASK.

As a further justification for the 5% point difference, our observations of physician performance evaluations associated with patient satisfaction scores in real world practices suggest that sometimes even a 1% difference could determine whether a physician would receive a performance reward or not. If a patient satisfaction metric is set at 80%, someone with a score of 79% will not receive a bonus pay, whereas someone with a 84% score would. The bonus pay could be 5% of one's salary and that is a non-trivial amount.

Tai-Seale has contributed to the research literature that suggests that patients' choice of physicians is influenced by report cards on patients' experience with physician practices.[1] We have also heard from some of the health system stakeholders on our study that they are eager to know even a 1% difference in a particular patient reported experience measure, i.e., the intent to recommend the physician they saw to family and friends. Our study will include this intent to recommend measure and other patient reported experience measures. We are confident that our study will produce salient information that will be meaningful to patients, physicians, and health care systems in decision making.

Appendix: Power calculations

The tables below show the power of the study to detect a difference in the proportion of respondents assigning *CollaboRATE* = 9 between the study arms, as follows: Table 1: Primary comparison, High Tech vs. ASK, superiority testing; Table 2: Secondary comparison, High Tech vs. High Touch, non-inferiority testing; Table 3: Additional secondary comparison, High Touch vs. ASK, superiority testing. In each case, five scenarios were considered: 1) study design as originally proposed (1-way ANOVA with Bonferroni-corrected post-hoc comparisons, 25+25 participants per PCP, difference of differences analysis); 2) comparison done at α=0.05, 25+25 participants per PCP, difference of differences analysis); 3) comparison done at α=0.05, 25+25 participants per PCP, compare follow-up time points only – this is in effect equivalent to a 0+25 participants per PCP; and primary comparison done at α=0.05, 10 baseline+40 follow-up participants per PCP, compared using 4) baseline and follow-up data, and 5) follow-up data only – the latter is in effect equivalent to a 0+40 participants per PCP.

Within each type of comparison, three scenarios for within-site ICC were considered, ICC_{Site}=0 (optimistic), ICC_{Site}=0.001 (realistic) and ICC_{Site}=0.002 (conservative). In all simulations we assume 7 sites per arm, and an average 5 PCP's per site, with a site-to-site coefficient of variation of 0.3 for the number of PCP's. Each PCP recruits 50 patients, for a total of 1,750 patients for each arm.

The within-PCP ICC is assumed ICC_{PCP}=0, as found in the pilot study (Tai-Seale et al., 2016). Due to this independence of patient ratings for the same physician, the "difference of differences" analysis achieves the same power as the follow-up only analyses (scenarios 2 vs. 3, and 4 vs. 5).

The power calculations were done using a custom statistical simulation program in R for all scenarios.

Table 1. Statistical power for the primary comparison of detecting differences of positive patient ratings between High Tech and ASK groups of 10%, 5%, and 3%, under five different scenarios. The scenarios differ in the type of analysis (DoD = difference of differences, adjusting for baseline; Fup = comparison of follow-up ratings only) and distribution of patients at

baseline and follow-up (25+25 = 25 baseline + 25 follow-up; 10+40 = 10 baseline + 40 follow-up). Each arm recruits 7 sites, with 5 PCP per site on average (CV=0.3) and 50 patients per PCP. The within-PCP ICC=0. Within-site ICC=0-0.002.

Primary comparison: High Tech vs. ASK	Power to detect **10%** difference (70% vs 80%)			Power to detect **5%** difference (72% vs 77%)			Power to detect **3%** difference (72% vs 75%)		
Study Design ICC_{PCP}=0.000	ICC_{Site} =0.000	ICC_{Site} =0.001	ICC_{Site} =0.002	ICC_{Site} =0.000	ICC_{Site} =0.001	ICC_{Site} =0.002	ICC_{Site} =0.000	ICC_{Site} =0.001	ICC_{Site} =0.002
1. 25+25 DoD α=0.05/3	>0.99	0.99	0.98	0.49	0.45	0.43	0.18	0.17	0.17
2. 25+25 DoD α=0.05	>0.99	>0.99	>0.99	0.66	0.63	0.60	0.32	0.31	0.30
3. 25+25 Fup α=0.05	>0.99	>0.99	>0.99	0.66	0.63	0.60	0.32	0.31	0.30
4. 10+40 DoD α=0.05	1.0	1.0	1.0	0.84	0.81	0.78	0.43	0.40	0.37
5. 10+40 Fup α=0.05	1.0	1.0	1.0	0.84	0.81	0.78	0.43	0.40	0.37

Table 2. Statistical power for the secondary comparison of non-inferiority evaluating the proportion of positive patient ratings between High Tech and High Touch groups of 10%, 5%, and 3%, under five different scenarios. The scenarios differ in the type of analysis (DoD = difference of differences, adjusting for baseline; Fup = comparison of follow-up ratings only) and distribution of patients at baseline and follow-up (25+25 = 25 baseline + 25 follow-up; 10+40 = 10 baseline + 40 follow-up). Each arm recruits 7 sites, with 5 PCP per site on average (CV=0.3) and 50 patients per PCP. The within-PCP ICC=0. Within-site ICC=0-0.002.

Primary comparison: High Tech vs. ASK	Power to detect **10%** non-inferiority margin (alternative 70% vs 80%)			Power to detect **5%** non-inferiority margin (alternative 72% vs 77%)			Power to detect **3%** non-inferiority margin (alternative 72% vs 75%)		
Study Design ICC_{PCP}=0.000	ICC_{Site} =0.000	ICC_{Site} =0.001	ICC_{Site} =0.002	ICC_{Site} =0.000	ICC_{Site} =0.001	ICC_{Site} =0.002	ICC_{Site} =0.000	ICC_{Site} =0.001	ICC_{Site} =0.002
1. 25+25 DoD α=0.025/3	>0.99	0.99	0.98	0.49	0.46	0.44	0.18	0.17	0.17
2. 25+25 DoD α=0.025(1)	>0.99	>0.99	>0.99	0.66	0.64	0.61	0.32	0.32	0.31
3. 25+25 Fup α=0.025(1)	>0.99	>0.99	>0.99	0.66	0.64	0.61	0.32	0.32	0.31
4. 10+40 DoD α=0.025(1)	1.0	1.0	1.0	0.84	0.82	0.79	0.43	0.41	0.38
5. 10+40 Fup α=0.025(1)	1.0	1.0	1.0	0.84	0.82	0.79	0.43	0.41	0.38

(1) Note: All tests are done one-sided, with the alternative showing that the High Tech arm is not inferior to the High Touch arm.

Table 3. Statistical power for the additional secondary comparison of detecting differences of positive patient ratings between High Touch and ASK groups of 10%, 5%, and 3%, under five different scenarios. The scenarios differ in the type of analysis (DoD = difference of differences, adjusting for baseline; Fup = comparison of follow-up ratings only) and distribution of patients at baseline and follow-up (25+25 = 25 baseline + 25 follow-up; 10+40 = 10 baseline + 40 follow-up). Each arm recruits 7 sites, with 5 PCP per site on average (CV=0.3) and 50 patients per PCP. The within-PCP ICC=0. Within-site ICC=0-0.002.

Primary comparison: High Tech vs. ASK	Power to detect **10%** difference (70% vs 80%)			Power to detect **5%** difference (72% vs 77%)			Power to detect **3%** difference (72% vs 75%)		
Study Design ICC_{PCP}=0.000	ICC_{Site} =0.000	ICC_{Site} =0.001	ICC_{Site} =0.002	ICC_{Site} =0.000	ICC_{Site} =0.001	ICC_{Site} =0.002	ICC_{Site} =0.000	ICC_{Site} =0.001	ICC_{Site} =0.002
1. 25+25 DoD α=0.05/3	>0.99	0.99	0.98	0.49	0.45	0.43	0.18	0.17	0.17
2. 25+25 DoD α=0.05	>0.99	>0.99	>0.99	0.66	0.63	0.60	0.32	0.31	0.30
3. 25+25 Fup α=0.05	>0.99	>0.99	>0.99	0.66	0.63	0.60	0.32	0.31	0.30
4. 10+40 DoD α=0.05	1.0	1.0	1.0	0.84	0.81	0.78	0.43	0.40	0.37
5. 10+40 Fup α=0.05	1.0	1.0	1.0	0.84	0.81	0.78	0.43	0.40	0.37

Supplement 1 Reference

1. Tai-Seale M, Elwyn G, Wilson CJ, Stults C, Dillon EC, Li M, Chuang J, Meehan A, Frosch DL. Enhancing shared decision making through carefully designed interventions that target patient and provider behavior. *Health Affairs* 2016; 35:605-612.
