# Supplementary material for: Patient-Clinician Communication Interventions Across Multiple Primary Care Sites: A Cluster Randomized Clinical Trial
Source: JAMA Health Forum. 2024 Dec 13;5(12):e244436. doi: 10.1001/jamahealthforum.2024.4436 (PMC11645648; doi:10.1001/jamahealthforum.2024.4436)
Supplement: Supplement 3. — eTable 1. Key Aspects of 3 Interventions eFigure. ASK Arm Poster eTable 2. Patient Characteristics by Treatment Arm in Baseline and Post-Intervention Phases eTable 3. Unadjusted Comparisons of CollaboRATE Outcomes Between Treatment Arms eTable 4. Comparison of CollaboRATE Between Treatment Arms, Adjusting for Health System and Patient and Clinician Characteristics eTable 5. Comparisons of Net Promoter Scores (NPS) by Treatment Arm, Adjusting for Covariates eTable 6. Comparison of Patient Confidence by Treatment Arm, Adjusting for Health System and for Patient and Clinician Characteristics [file jamahealthforum-e244436-s003.pdf]

Supplemental Online Content

Tai-Seale M, Cheung M, Vaida F, et al. Patient-clinician communication interventions across multiple primary care sites: a cluster randomized clinical trial. *JAMA Health Forum.* 2024;5(12):e244436. doi:10.1001/jamahealthforum.2024.4436

eTable 1. Key Aspects of 3 Interventions

eFigure. ASK Arm Poster

eTable 2. Patient Characteristics by Treatment Arm in Baseline and Post-Intervention Phases

eTable 3. Unadjusted Comparisons of CollaboRATE Outcomes Between Treatment Arms

eTable 4. Comparison of CollaboRATE Between Treatment Arms, Adjusting for Health System and Patient and Clinician Characteristics

eTable 5. Comparisons of Net Promoter Scores (NPS) by Treatment Arm, Adjusting for Covariates

eTable 6. Comparison of Patient Confidence by Treatment Arm, Adjusting for Health System and for Patient and Clinician Characteristics

This supplemental material has been provided by the authors to give readers additional information about their work.

© 2024 Tai-Seale M et al. *JAMA Health Forum*.

eTable 1. Key Aspects of 3 Interventions

<table>
<tr><th></th><th>High-Touch</th><th>High-Tech</th><th>ASK</th></tr>
<tr><td rowspan="3">Patient</td><td colspan="2">EHR Patient Portal-Based Appointment Reminder to Patient
• Link to single item questionnaire: “What do you most want to discuss at your upcoming visit?”
• Invitation to participate in study
• Link to online informed consent and enrollment portal</td><td>• Invitation to participate in study
• Link to online informed consent and enrollment portal</td></tr>
<tr><td colspan="2">• Link to animated video for patient to watch before visit- encourages patients to:
− Share concerns and indicate which is most important
− Teachback next steps to clinician</td><td>Patient sees poster with 3 ASK questions in the exam room (See eFigure in the Supplement)</td></tr>
<tr><td colspan="3">Post-Visit Questionnaire Sent to Patient
• Items assessing patient’s perception of the encounter
• Items assessing patient’s understanding of next steps and intent to complete next steps</td></tr>
<tr><td>Medical Assistant (MA)</td><td colspan="2">MA Engages Patient at Visit
• Reviews most important agenda item, annotates if needed
• Ensures prominence of item for clinician review</td><td>MA Performs Standard Rooming
May/may not highlight patient’s most important issue</td></tr>
<tr><td>Clinician</td><td>In–Person Standardized Patient Instructor (SPI) for Clinician
Visit 1: 30-minute session with SPI for Introduction, Instruction, and Role Play to:
- Understand the patient’s top concern
- Acknowledge Patient Agenda
- Negotiate Joint Agenda
- Shared decision making
- Teachback from Patient
- Incorporate next steps into After Visit Summary

Clinician records actual “practice patient” encounter – gives to SPI

Visit 2: 30-minute session with SPI for feedback on practice patient, further instruction on skills and role play</td><td>Mobile App Standardized Patient Instructor (SPI) Training for Clinician
Multiple brief mobile virtual coaching sessions to teach and practice:
- Understand the patient’s top concern
- Acknowledge Patient Agenda
- Negotiate Joint Agenda
- Shared decision making
- Teachback from Patient
- Incorporate next steps into After Visit Summary</td><td>Clinician sees poster with 3 ASK questions in exam room (See eFigure in the Supplement)</td></tr>
<tr><td rowspan="2">Research Team</td><td colspan="3">Electronic Data Extraction
EHR data extracted: number and timing of post-visit telephone calls, emails and follow-up visits</td></tr>
<tr><td colspan="3">In-depth EHR Review
Review of subsample of high utilization patients’ EHR to determine reasons for calls, emails, visits, and link between utilization and most important concern.</td></tr>
</table>

© 2024 Tai-Seale M et al. *JAMA Health Forum*.

eFigure. ASK Arm Poster

During your appointment, there may be choices to make about your healthcare

Durante su cita, es posible que tenga que decidir entre opciones acerca de su cuidado de la salud.

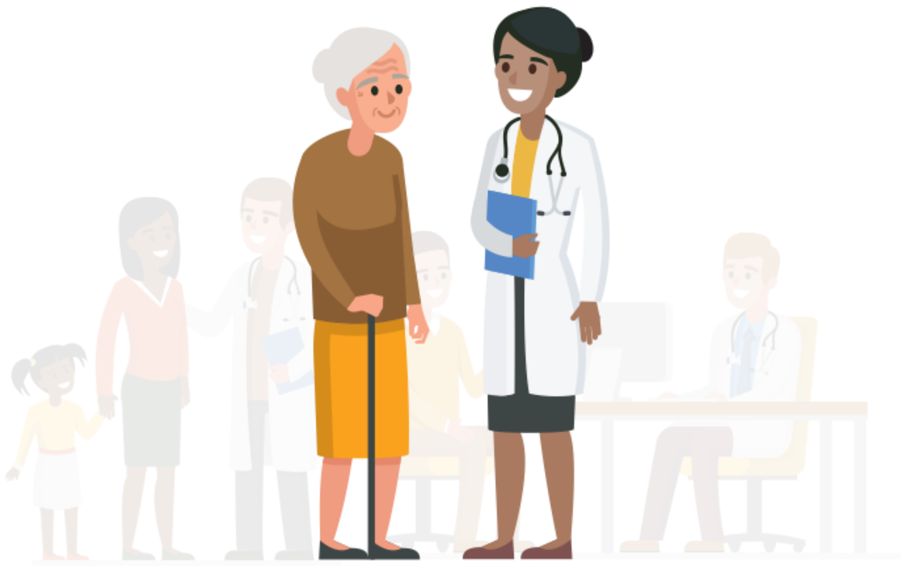

As you make a plan for next steps, be sure to get the answers **to three important questions:**

Mientras planifica sus próximos pasos, asegúrese de que le respondan a estas **tres preguntas importantes:**

1 What are my options?
¿Cuáles son mis opciones?

2 What are the possible benefits and risks of each option?
¿Cuáles son los posibles beneficios y riesgos de cada opción?

3 How likely are each of the benefits and risks to happen to me?
¿Qué probabilidades hay de que esos beneficios y riesgos me afecten?

UC San Diego Health

Open and Ask

© 2024 Tai-Seale M et al. *JAMA Health Forum*.

eTable 2. Patient characteristics by treatment arm in baseline and post-intervention phases

	Baseline Phase				Post-Intervention Phase			
	**ASK (N=465)**	**Tech (N=381)**	**Touch (N=319)**	**P-value**	**ASK (N=1,432)**	**Tech (N=1,129)**	**Touch (N=1,126)**	**P-value**
Age, Median (IQR) year	56.0 (41, 66.1)	52.0 (40, 64.7)	52.0 (40.9, 63)		58.0 (42, 68)	53.0 (39, 65)	52.0 (36, 64)	
**Sex, No. (%)**								
Female	279 (60.0 %)	262 (68.8 %)	194 (60.8 %)	0.0205	858 (59.9 %)	761 (67.4 %)	732 (65.0 %)	<0.001
Male	186 (40.0 %)	119 (31.2 %)	125 (39.2 %)		541 (37.8 %)	366 (32.4 %)	336 (29.8 %)	
Other or missing					33 (2.3 %)	2 (0.2 %)	58 (5.2 %)	
**Race**								
American Indian or Alaska Native	1 (0.2 %)	1 (0.3 %)	1 (0.3 %)	0.497	6 (0.4 %)	4 (0.4 %)	7 (0.6 %)	0.0015
Asian	56 (12.0 %)	63 (16.5%)	49 (15.4 %)		123 (8.6 %)	123 (10.9 %)	144 (12.8 %)	
Black or African American	11 (2.4 %)	13 (3.4 %)	6 (1.9 %)		26 (1.8 %)	36 (3.2 %)	19 (1.7 %)	
More than 1 race					33 (2.3 %)	29 (2.6 %)	35 (3.1 %)	
Native Hawaiian or other Pacific Islander	7 (1.5 %)	7 (1.8 %)	4 (1.3 %)		3 (0.2 %)	9 (0.8 %)	5 (0.4 %)	
White	318 (68.4 %)	238 (62.5 %)	197 (61.8 %)		1219 (85.1 %)	895 (79.3 %)	903 (80.2 %)	
Missing	72 (15.5%)	59 (15.5 %)	62 (19.4 %)		22 (1.5 %)	33 (2.9 %)	13 (1.2 %)	
**Ethnicity**								
Hispanic	24 (5.2 %)	32 (8.4 %)	12 (3.8 %)	<0.001	105 (7.3 %)	119 (10.5 %)	97 (8.6 %)	0.017
Non-Hispanic	278 (59.8 %)	256 (67.2 %)	185 (58.0 %)		1314 (91.8 %)	1001 (88.7 %)	1020 (90.6 %)	
Missing	163 (35.1 %)	93 (24.4 %)	122 (38.2%)		13 (0.9 %)	9 (0.8 %)	9 (0.8 %)	

© 2024 Tai-Seale M et al. *JAMA Health Forum*.

eTable 2. Continued

	Baseline Phase				Post-Intervention Phase			
	ASK (N=465)	Tech (N=381)	Touch (N=319)	P-value	ASK (N=1,432)	Tech (N=1,129)	Touch (N=1,126)	P-value
**Education**								
8th grade or less					3 (0.2 %)	0 (0.0 %)	2 (0.2 %)	0.0025
Some high school, but did not graduate					4 (0.3 %)	7 (0.6 %)	10 (0.9 %)	
High school graduate or GED					112 (7.8 %)	55 (4.9 %)	61 (5.4 %)	
Some college or 2-year degree					329 (23.0 %)	232 (20.5 %)	227 (20.2 %)	
4-year college graduate					349 (24.4 %)	316 (28.0 %)	335 (29.8 %)	
More than 4-year college degree					634 (44.3 %)	517 (45.8 %)	490 (43.5 %)	
Missing	465 (100%)	381 (100%)	319 (100%)		1 (0.1 %)	2 (0.2 %)	1 (0.1 %)	

Abbreviation: SD, standard deviation
[a] Comparisons use one-way ANOVA F-test for continuous variables and Pearson's chi-squared test for categorical variables.

© 2024 Tai-Seale M et al. *JAMA Health Forum*.

eTable 3. Unadjusted comparisons of CollaboRATE outcomes between treatment arms[a]

	CollaboRATE (N=4,819)		
	ROR[b]	95% CI	P-value
High Tech vs ASK	0.906	(0.711, 1.153)	0.421
High Touch vs ASK	0.918	(0.723, 1.165)	0.480
High Tech vs High Touch[c]	0.987	(0.768, 1.269)	0.023[c]

[a]The analysis uses longitudinal mixed-effects logistic regression, including random intercepts for PCP.
[b]The treatment effects are expressed as ratios of odds ratios (ROR) for post-intervention vs. baseline phase, compared between treatment arms. P-values are from the Wald test.
[c]The test for the non-inferiority comparison of OPEN High Tech against OPEN High Touch is one-sided performed at the α=0.025 level, with a pre-specified non-inferiority limit of 0.765 for CollaboRATE.

© 2024 Tai-Seale M et al. *JAMA Health Forum*.

eTable 4. Comparison of CollaboRATE between treatment arms, adjusting for health system and patient and clinician characteristics[a]

	CollaboRATE (N=2,066)			
	**OR**	**95% CI**	**P-value**	**Average Marginal Effect (95% CI)**
**Arm comparisons, ROR[b] (and 95% CI)**				
High Tech vs ASK	1.013	(0.700, 1.464)	0.95	0.002 (-0.067, 0.072)
High Touch vs ASK	1.089	(0.749, 1.583)	0.66	0.016 (-0.054, 0.085)
High Tech vs High Touch[c]	0.930	(0.642, 1.346)	0.15 [a]	-0.013 (-0.082, 0.055)
**Covariates, OR (and 95% CI)**				
Patient Age (per year)	1.009	(1.002, 1.016)	0.01	0.002 (0.000, 0.003)
Patient Sex: Non-Female or missing (ref = Female)	0.680	(0.538, 0.859)	0.002	-0.074 (-0.119, -0.029)
Patient Race: Non-White (ref = White)	0.877	(0.667, 1.153)	0.35	-0.025 (-0.078, 0.028)
Patient SVI	1.146	(0.724, 1.813)	0.56	0.025 (-0.057, 0.107)
Patient Confidence in Ability to Take Care of Health	1.817	(1.644, 2.000)	< 0.001	0.101 (0.087, 0.114)
Patient Encounter Type: Televisit (ref = In-Person)	0.597	(0.397, 0.899)	0.01	-0.103 (-0.188, -0.018)
Patient Encounter Type: Visit Type Unknown (ref = In-Person)	0.834	(0.639, 1.089)	0.18	-0.034 (-0.084, 0.016)
Patient Visit Reason: Acute (ref = Non-Acute)	0.809	(0.635, 1.030)	0.09	-0.041 (-0.087, 0.006)
Patient Index Visit on/after COVID-19 Emergency Declaration	1.031	(0.798, 1.333)	0.81	0.006 (-0.042, 0.054)
Clinician Sex: Non-Female or missing (ref = Female)	1.090	(0.810, 1.469)	0.57	0.016 (-0.039, 0.072)
Clinician Race: Non-White (ref = White)	0.689	(0.519, 0.915)	0.01	-0.071 (-0.126, -0.016)
Clinician Specialty: Non-Family Medicine (ref = Family Medicine)	0.870	(0.632, 1.196)	0.39	-0.026 (-0.086, 0.034)
Clinician Time Since Residency (per year)	1.007	(0.993, 1.022)	0.33	0.001 (-0.001, 0.004)
HS2 (ref = HS1)	0.596	(0.417, 0.852)	0.004	-0.097 (-0.167, -0.028)
HS3 (ref = HS1)	0.717	(0.490, 1.048)	0.086	-0.062 (-0.134, 0.010)

Abbreviations: CI, Confidence Interval; HS1, Health System 1; HS2, Health System 2; HS3, Health System 3; OR, Odds Ratio; Ref, Reference; SVI, Social Vulnerability Index
[a]The analysis uses longitudinal mixed-effects logistic regression, including random intercepts for clinician.
[b]The treatment effects are expressed as ratios of odds ratios (ROR) for post-intervention vs. baseline phase, compared between treatment arms.
[c]The test for the non-inferiority comparison of OPEN High Tech against OPEN High Touch is one-sided performed at the α = 0.025 level, with pre-specified non-inferiority limits of 0.765 for CollaboRATE.

© 2024 Tai-Seale M et al. *JAMA Health Forum*.

eTable 5. Comparisons of Net Promoter Scores (NPS) by treatment arm, adjusting for covariates[a, b]

(N = 2,077)	OR[b,c]	95% CI	P-value	Average Marginal Effect (95% CI)[d]	
				Promoter	Detractor
High Tech vs ASK	0.809	(0.486, 1.348)	0.415	-0.024 (-0.098, 0.054)	0.007 (-0.018, 0.032)
High Touch vs ASK	1.398	(0.816, 2.393)	0.222	0.032 (-0.045, 0.103)	-0.009 (-0.032, 0.014)
High Tech vs High Touch	0.579	(0.342, 0.978)	0.041[c]	-0.056 (-0.118, 0.019)	0.017 (-0.006, 0.038)
Patient Age (years)	1.017	(1.008, 1.026)	< 0.001	0.002 (0, 0.003)	-0.001 (-0.001, 0)
Patient Sex: Non-Female or missing (ref = Female)	1.089	(0.799, 1.483)	0.590	0.001 (-0.011, 0.085)	-0.006 (-0.094, 0.018)
Patient Race: Non-White (ref = White)	0.957	(0.671, 1.365)	0.809	0.001 (-0.011, 0.085)	-0.006 (-0.095, 0.018)
Patient SVI (per 0.1 units)	1.010	(0.951, 1.072)	0.754	0.001 (-0.008, 0.009)	0 (-0.003, 0.003)
Patient Confidence in Ability to Take Care of Health	1.854	(1.669, 2.058)	< 0.001	0.055 (0.042, 0.069)	-0.016 (-0.023, -0.011)
Patient Encounter Type: Televisit (ref = In-Person)	0.608	(0.365, 1.015)	0.057	0.025 (-0.036, 0.12)	-0.026 (-0.101, 0.035)
Patient Encounter Type: Visit Type Unknown (ref = In-Person)	0.837	(0.582, 1.204)	0.339	0.026 (-0.036, 0.118)	-0.025 (-0.098, 0.033)
Patient Visit Reason: Acute (ref = Non-Acute)	0.670	(0.494, 0.909)	0.010	0.026 (-0.036, 0.118)	-0.025 (-0.098, 0.034)
Patient Index Visit on/after COVID-19 Emergency Declaration	0.932	(0.662, 1.314)	0.689	0.026 (-0.036, 0.117)	-0.025 (-0.096, 0.033)
Clinician Sex: Non-Female or missing (ref = Female)	0.909	(0.591, 1.399)	0.665	0.026 (-0.036, 0.117)	-0.025 (-0.097, 0.033)
Clinician Race: Non-White (ref = White)	0.796	(0.526, 1.206)	0.282	0.026 (-0.037, 0.116)	-0.025 (-0.097, 0.033)
Clinician Specialty: Non-Family Medicine (ref = Family medicine)	0.624	(0.392, 0.993)	0.047	0.026 (-0.037, 0.118)	-0.025 (-0.098, 0.033)
Clinician Time Since Residency (years)	1.002	(0.981, 1.023)	0.873	0 (-0.003, 0.003)	0 (-0.001, 0.001)
HS2 (ref = HS1)	0.605	(0.350, 1.045)	0.072	0 (0, 0.013)	0 (-0.016, 0)
HS3 (ref = HS1)	0.566	(0.335, 0.958)	0.034	0 (0, 0.013)	0 (-0.016, 0)

Abbreviations: CI, Confidence Interval; HS1, Health System 1; HS2, Health System 2; HS3, Health System 3; OR, Odds Ratio; MD, Mean Difference; Ref, Reference

[a]The analysis uses longitudinal mixed-effects ordinal logistic regression (MEOLR) and linear mixed-effects regression (LMER) models, including a random intercept for clinicians. The ordinal outcome is NPS (promoter > neutral > detractor).

[b]ROR, odds ratios of higher vs lower NPS levels from ordinal logistic regression. For treatment arm comparisons these are ratios of odds ratios for post-intervention compared to baseline phase. P-values are from the likelihood ratio test.

[c]The comparisons of treatment arms are expressed in terms of ratios of odds ratios (ROR) for post-intervention vs. baseline phase, compared between treatment arms.

[d]Confidence intervals for average marginal effects calculated by bootstrap with 10000 samples.

© 2024 Tai-Seale M et al. *JAMA Health Forum*.

eTable 6. Comparison of patient confidence [a] by treatment arm, adjusting for health system and for patient and clinician characteristics

	Post Index vs Baseline Visit (N=2,083)[b]			3-Month Post Index Visit (N=1,679)[c]		
	**MD**	**95% CI**	**P-value**	**MD**	**95% CI**	**P-value**
High Tech vs ASK	-0.025	(-0.177, 0.127)	0.748	-0.059	(-0.235, 0.117)	0.512
High Touch vs ASK	0.023	(-0.130, 0.177)	0.766	0.117	(-0.061, 0.295)	0.205
High Tech vs High Touch	-0.048	(-0.192, 0.096)	0.510	-0.176	(-0.341, -0.011)	0.037
Patient Age (years)	0.007	(0.003, 0.010)	< 0.001	0.003	(-0.001, 0.007)	0.119
Patient Sex: Non-Female or missing (ref = Female)	-0.122	(-0.232, -0.011)	0.031	-0.189	(-0.325, -0.052)	0.007
Patient Race: Non-White (ref = White)	0.034	(-0.096, 0.164)	0.607	-0.102	(-0.267, 0.063)	0.227
Patient SVI	-0.065	(-0.278, 0.147)	0.547	-0.086	(-0.345, 0.173)	0.517
Patient Encounter Type: Televisit (ref = In-Person)	-0.029	(-0.224, 0.167)	0.775	0.127	(-0.111, 0.365)	0.296
Patient Encounter Type: Visit Type Unknown (ref = In-Person)	-0.014	(-0.135, 0.107)	0.823	0.106	(-0.037, 0.249)	0.147
Patient Visit Reason: Acute (ref = Non-Acute)	-0.138	(-0.251, -0.025)	0.017	-0.056	(-0.196, 0.084)	0.431
Patient Index Visit on/after COVID-19 Emergency Declaration	0.051	(-0.068, 0.169)	0.401	0.061	(-0.077, 0.199)	0.387
Clinician Sex: Non-Female or missing (ref = Female)	0.014	(-0.112, 0.141)	0.825	0.002	(-0.151, 0.156)	0.976
Clinician Race: Non-White (ref = White)	-0.004	(-0.122, 0.114)	0.947	-0.088	(-0.230, 0.055)	0.236
Clinician Specialty: Non-Family Medicine (ref = Family Medicine)	-0.041	(-0.176, 0.093)	0.548	0.049	(-0.115, 0.213)	0.562
Clinician Time Since Residency (years)	0.002	(-0.004, 0.008)	0.477	0.000	(-0.007, 0.008)	0.918
HS2 (ref = HS1)	-0.32	(-0.487, -0.152)	< 0.001	-0.144	(-0.337, 0.050)	0.149
HS3 (ref = HS1)	-0.052	(-0.200, 0.095)	0.49	-0.153	(-0.329, 0.023)	0.095

Abbreviations: CI, Confidence Interval; HS1, Health System 1; HS2, Health System 2; HS3, Health System 3; MD, Mean Difference; Ref, Reference; SVI, Social Vulnerability Index

[a]"Overall, how confident are you about your ability to take good care of your health?"

[b]The analysis uses longitudinal mixed−effects linear regression model, including a random intercept for clinicians. Baseline means are assumed equal for the three treatment arms, adjusted for covariates. The comparisons of treatment arms are expressed in terms of differences in differences, i.e., mean change for post-intervention vs. baseline phase, compared between treatment arms. Covariate effects are expressed as linear mean difference estimates (MD) between groups.

[c]The analysis uses mixed−effects linear regression model, including a random intercept for clinicians. The treatment effects are expressed as linear mean difference estimates (MD) compared between treatment arms. Covariate effects are expressed as linear mean difference estimates (MD) between groups.

© 2024 Tai-Seale M et al. *JAMA Health Forum*.
