# Supplementary material for: Patient-Clinician Communication Interventions Across Multiple Primary Care Sites: A Cluster Randomized Clinical Trial
Source: JAMA Health Forum. 2024 Dec 13;5(12):e244436. doi: 10.1001/jamahealthforum.2024.4436 (PMC11645648; doi:10.1001/jamahealthforum.2024.4436)
Supplement: Supplement 4. — Data Sharing Statement [file jamahealthforum-e244436-s004.pdf]

Data Sharing Statement

Tai-Seale. Patient-Clinician Communication Interventions Across Multiple Primary Care Sites. *JAMA Health Forum*. Published December 13, 2024. doi:10.1001/jamahealthforum.2024.4436

Data
Additional Information: Trial registry: clinicaltrials.gov ClinicalTrials.gov ID: NCT03385512
URL: https://clinicaltrials.gov/study/NCT03385512
Data available: No
